# Homochirality of β‐Peptides: A Significant Biomimetic Property of Unnatural Systems

**DOI:** 10.1002/open.201700078

**Published:** 2017-07-20

**Authors:** István M. Mándity, Imane Nekkaa, Gábor Paragi, Ferenc Fülöp

**Affiliations:** ^1^ Institute of Pharmaceutical Chemistry University of Szeged Eötvös u. 6 6720 Szeged Hungary; ^2^ MTA-SZTE Supramolecular and Nanostructured Materials Research Group Dóm tér 8 6720 Szeged Hungary; ^3^ Research Group of Stereochemistry of the Hungarian Academy of Sciences Dóm tér 8 6720 Szeged Hungary

**Keywords:** chirality, diastereoselectivity, foldamer, homochiralty, β-peptide

## Abstract

Homochirality, an interesting phenomenon of life, is mainly an unresolved problem and was thought to be a property of living matter. Herein, we show that artificial β‐peptides have the tendency toward homochiral diastereoselective chain elongation. Chain‐length‐dependent stereochemical discrimination was investigated in the synthesis of foldamers with various side chains and secondary structures. It was found that there is a strong tendency toward the synthesis of homochiral oligomers. The size of the side chain drastically influenced the selectivity of the stereodiscriminative chain‐elongation reaction. It is noteworthy that water as the co‐solvent increases the selectivity. Such behavior is a novel fundamental biomimetic property of foldamers with a potential of future industrial application.

Homochirality is an inherent property of vital polymers, for example, peptides, proteins, RNA, DNA, as well as oligo‐ and polysaccharides.^1^ It is still not clear whether homochirality is a result of an autocatalytic procedure^2^ or if it originates from extraterrestrial optically active non‐racemic mixtures.^3^ Numerous investigations have been carried out by various research groups to investigate the origin and role of this phenomenon.1c, 4 In the case of peptides, mainly the stereochemical discrimination in their synthesis has been investigated. For example, a racemate of activated amino acids was reacted with a given enantiomerically pure amino acid or peptide chain. The results reveal a strong tendency for the formation of homochiral sequences.^5^ This effect is even more dominant when water is utilized as a co‐solvent.5a In the case of self‐templated peptide fragment coupling, again, homochiral compounds are formed.^6^ On the other hand, when dipeptides were created, heterochiral sequences appeared.^7^ In the case of peptides containing strongly structure‐promoting α,α‐disubstituted amino acid, the formation of heterochiral sequences is favored when large and bulky side chains are utilized.^8^ However, for smaller side chains, the synthesis of homochiral peptides is preferred.^9^ For similar sequences, helicity alone can govern the direction of enantioselectivity. For example, *P*‐helices favor the incorporation of l‐amino acids, whereas *M*‐helices prefer the coupling of the *d*‐enantiomer.^10^

Foldamers are artificial self‐organizing biomimetic polymers.^11^ These systems have a strong tendency to form highly stable and versatile secondary structures, for example, helices, strands, turns, and so on.11g, 12 Foldamers have numerous biomedical activities.^13^ They are antibacterial amphiphills,^14^ cell and membrane penetrating peptides,^15^ *anti*‐Alzheimer compounds,^16^ and effectively modulate protein–protein interactions.^17^ Based on this fact, they are nowadays considered as proteomimetics.13a Some of the most thoroughly investigated foldamers are the β‐peptides,11d–11f, 18 which possess additional biomimetic properties akin to natural α‐peptides including hierarchical self‐organization,^19^ conformational polymorphism, and real folding reactions.^20^

Herein, we show that stereochemical discrimination towards the homochiral oligomers manifests for β‐peptides too, akin to the biological homochirality of natural polymers. The phenomenon can be observed for various β‐peptides with different secondary structures, side‐chain shape, and chain length.

β‐Peptide foldamers containing various cyclic side chains and possessing different secondary structures were selected for the examination of diastereo‐discriminative peptide coupling with chain lengths varying from trimer to hexamer. As a reference, α‐l‐leucine homooligopeptides were selected. The investigated structures are shown in Scheme 1.

**Scheme 1:**
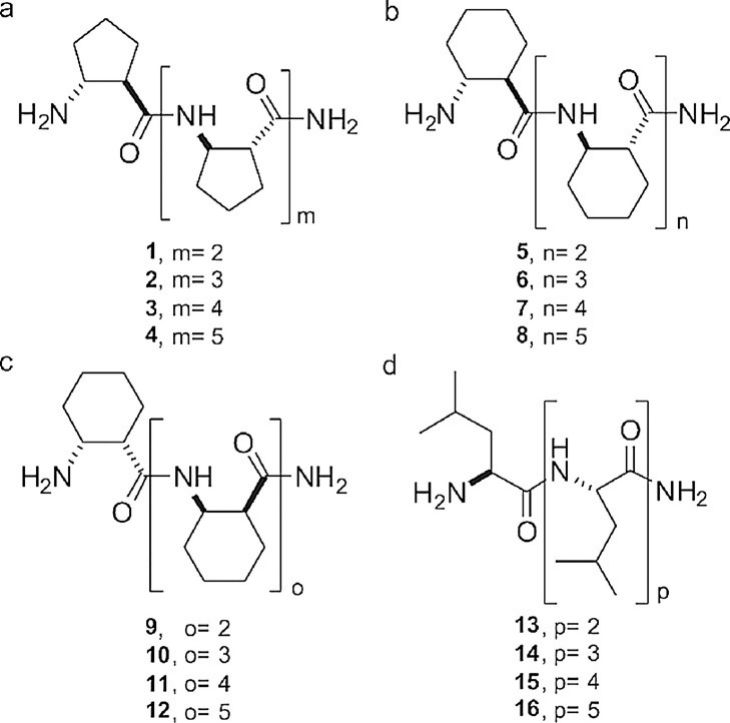
The investigated structures possessing five‐ and six‐membered side chains and *cis* or *trans* relative configuration.

Oligomers **1**–**4** are composed of [1*R*,2*R*]‐*trans*‐2‐aminocyclopentanecarboxylic acid ([1*R*,2*R*]‐*trans*‐ACPC) building blocks and they are known to form H12 helices.11b As a next step, for compounds **5**–**8**, the five‐membered cyclic side chains were changed for a six‐membered cyclohexane ring, while retaining the same stereochemistry. These oligomers were constructed from [1*R*,2*R*]‐*trans*‐2‐aminocyclohexanecarboxylic acid ([1*R*,2*R*]‐ACHC) residues.11a The formed secondary structure is a H14 helix. To investigate the effect of a strand structure, oligomers created from [1*S*,2*R*]‐*cis*‐ACHC were also assembled (β‐peptides **9**–**12**). Homochiral homooligomers made from *cis*‐β‐amino acids are known to form strand structures.^19^, ^21^ This fact is further supported by a stereochemical patterning approach, which declares that β‐peptides possessing different stereochemistry on the two sides of an amide bond form a strand structure.12b

As control structures, homooligomeric α‐peptides composed of l‐Leu were synthesized too (**13**–**16**). The isobutyl side chain was selected in accordance with the β‐amino acids described above, as only β‐peptides comprising aliphatic side chains were tested.

The peptides were assembled by utilizing highly efficient continuous‐flow solid‐phase peptide synthesis technology developed in our laboratory.^22^ The technology allows the construction of various peptides by using very low, generally 1.5‐fold, amino‐acid equivalents. The peptides were elongated in the instrument, and the products were cleaved and purified through regular reversed‐phase HPLC methodology.

The stereochemical discrimination properties of the β‐peptides were tested by means of a solution‐phase peptide‐coupling technique. The purified peptides were dissolved in a 2:1 mixture of dichloromethane (DCM) and *N*,*N*‐dimethylformamide (DMF). Subsequently, 10 equivalents of the *tert*‐butyloxycarbonyl (Boc)‐protected racemic amino acid was coupled by using *N*,*N′*‐diisopropylcarbodiimide (DIC) and hydroxybenzotriazole (HOBt) coupling agents. The reaction time was 48 h. Importantly, the effect of water on the stereochemical discrimination was also investigated, as it is known to be a crucial factor.5a, 6 Thus, the reactions described above were performed in the solvent system DCM/DMF/H_2_O 2:1:1 with a reaction time of 48 h. The solvents were removed in vacuo and Boc deprotection was carried out by TFA treatment. Finally, the product was lyophilized and the raw material was analyzed by using HPLC–MS. The complete procedure is shown in Scheme 2 with the example of oligomers built from *trans*‐ACPC. The homochiral chain‐elongated reference compounds were always synthetized independently and the heterochiral diastereomers were deduced from the HPLC–MS chromatogram.

**Scheme 2:**
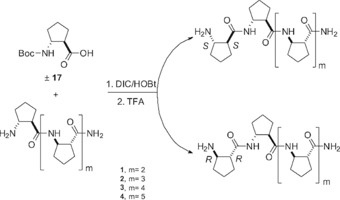
The chain elongation of a β‐peptide with a Boc‐protected racemic amino acid yielding two diastereomers shown by the example of the *trans*‐ACPC residue.

Based on the area integrals of the homochiral and heterochiral peptides, the diastereomeric excess (*DE*) of the reaction was calculated for all peptides in both the presence and the absence of water. The *DE* values are shown in Figure 1 in a chain‐length‐dependent manner.

**Figure 1:**
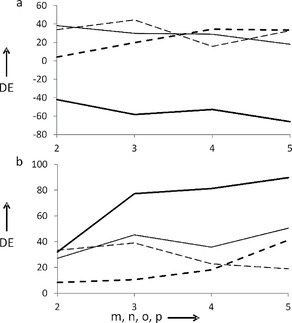
*DE* values obtained for **1**–**4** (thin solid line), **5**–**8** (thick solid line), **9**–**12** (thin dashed line), and **13**–**16** (thick dashed line) in the absence (a) and in the presence of water (b) as a function of chain length.

In the absence of water, a strong tendency towards the homochiral structures was observable for peptides containing the [1*R*,2*R*]‐*trans*‐ACPC unit (**1**–**4**). A slight decrease in selectivity was found as a function of chain length. The situation is completely different for the oligomers composed of [1*R*,2*R*]‐*trans*‐ACHC (**5**–**8**), which contain a more bulky side chain. The selectivity changed, the formation of the heterochiral product became favored, and a nice chain‐length‐dependent correlation was found. A change in selectivity towards the heterochiral sequences in a stereo‐discriminative chain elongation was observed for peptides composed of α,α‐disubstituted amino acids with large bulky side chains.^9^ A simple change in the side chain caused a drastic difference in the stereochemical discrimination properties of β‐peptides. To investigate the effect of *cis* relative configuration, compounds containing [1*S*,2*R*]‐*cis*‐ACHC (**9**–**12**) were also studied. Again, a definite tendency was observed towards the homochiral oligomers. The chain length did not significantly alter the *DE* value. As a reference, α‐peptides composed of l‐Leu were investigated. The diastereoselectivity showed a clear chain‐length‐dependent manner and the homochiral product was favored. Importantly, the chain elongation of the trimer l‐Leu showed only very minor diastereo‐differentiation.

In general, the presence of water enhanced the selectivity toward the formation of homochiral homooligomers. In the case of peptides built of [1*R*,2*R*]‐*trans*‐ACPC, *DE* values increased compared to those formed in non‐aqueous experiments and a clear chain‐length‐dependent increase could also be observed. The most dramatic difference compared to the experiment performed without water can be observed for oligomers comprising [1*R*,2*R*]‐*trans*‐ACHC. The selectivity changed to the opposite direction and homochiral compounds were profoundly formed during the coupling of the Boc‐protected racemic *trans*‐ACHC. In this case, a more dominant chain‐length‐dependent fashion can be observed and, importantly, the diastereoselective chain elongation of the hexamer reached a *DE* of almost 90 %. The effect of *cis* relative configuration was also investigated in the presence of water. The results indicate again the profound formation of the homochiral oligomer. Interestingly, *DE* values decreased to some extent as a function of chain length. The reference l‐Leu oligomers preferred the incorporation of the homochiral amino acids in a chain‐length‐dependent manner. Nonetheless, water, in general, increased the *DE* values compared to the results of the water‐free experiments.

To understand the effect of water in the diastereoselective chain‐elongation reaction, the effect of water on the foldameric systems itself should be considered. Foldameric systems are known to show secondary structure‐dependent self‐association with water as the solvent.^19^, ^20^ Helical structures self‐associate into vesicles, whereas strand structures form nano‐sized fibrils. It has been reported that the self‐association of peptides is a crucial factor behind biological homochirality.5a, 6 Consequently, we turned to investigate the effect of self‐association of β‐peptides on the stereochemical discrimination by means of theoretical calculations. Oligomer **7**, containing *trans*‐ACHC, has been selected as an example, as it showed the most dramatic water‐dependent diastereo‐discrimination.

Having a draft picture about the reaction barriers or possible steric hindrances, DFT calculations were carried out at the OLYP/tz2p level of theory.^23^ This method can provide acceptable geometries or reaction barriers for organic compounds.^24^ Transition states were calculated for the systems composed of Boc‐protected [1*R*,2*R*]‐ and [1*S*,2*S*]‐*trans*‐ACHC hydroxybenzotriazole ester and oligomer **6**. The structure of the latter was previously optimized in the form of a H14 helix. The reason for selecting β‐peptide **6** is that it forms a helical structure with the shortest chain length,11a which is the optimum structure for quantum chemical calculations with acceptable calculation times. Solvent effects were taken into account by means of the COSMO solvent model, utilizing the permittivity of chloroform and water.^25^ The calculated relative barrier energies are shown in Table 1.

**Table 1:** Relative barrier energies (in kcal mol^−1^) for the transition state of the chain elongation **6** in water and chloroform.

Solvent	Homochiral chain elongation	Heterochiral chain elongation
chloroform	2.4	1.0
water	4.8	0.0

The reaction barrier of the heterochiral construct was found to be lower than those for the homochiral one in both solvents. Consequently, the simplified model system cannot reproduce the changes in the trends caused by the absence or presence of water.

However, investigating the 3D structure of the homo‐ and heterochirally elongated compounds, self‐association might increase geometrical strain. To examine the effect of self‐association on the stereochemical discrimination in the chain elongation of β‐peptides, further theoretical calculations were carried out and first a membrane segment was constructed and optimized by utilizing the MMFF94^26^ force field. The assembly is shown in Figure 2 a. For this purpose, oligomer **7** was selected, as it has been reported to form solely a H14 helix.^20^

**Figure 2:**
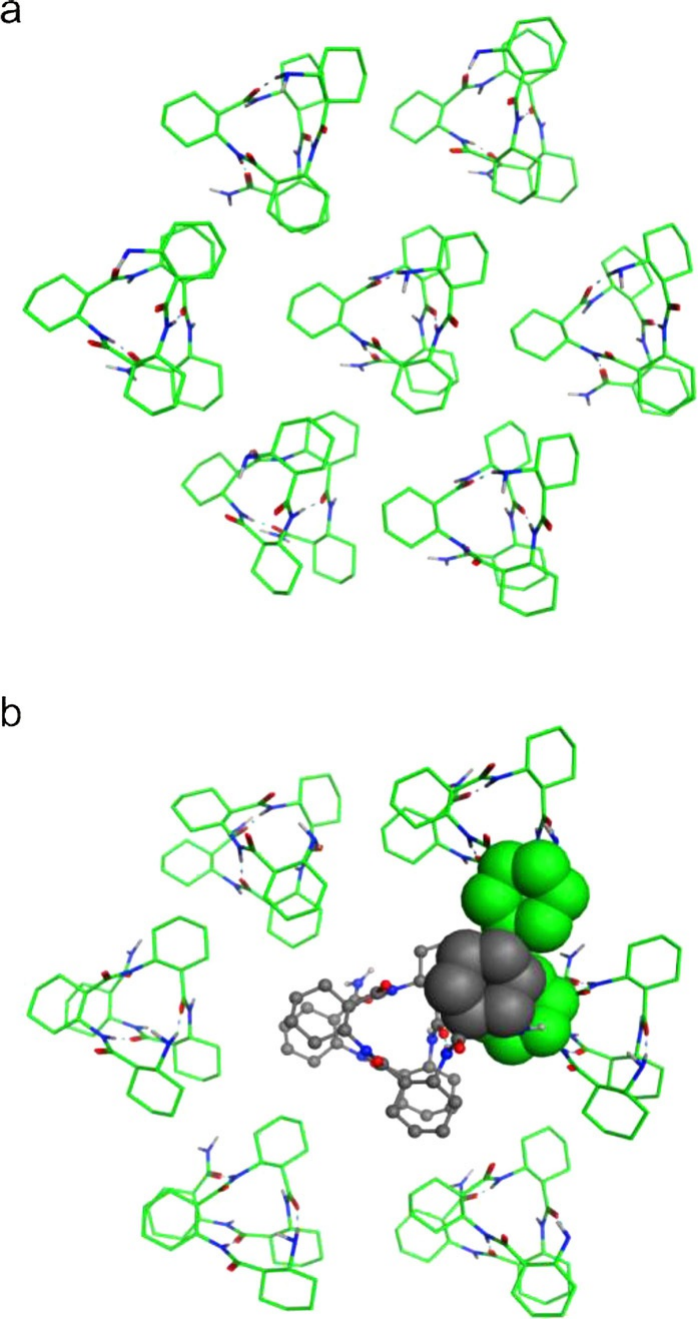
a) Molecular model for the vesicular membrane segment formed of **7** (top view). b) The incorporation of the heterochirally chain‐elongated construct to the membrane (grey‐colored ball‐and‐stick representation). The clashes of the side chains are depicted by the overlapping of the space filling model of the involved residues.

Our effort to perform similar density functional investigations and calculate the height of the barrier was not successful, because of the size of the systems. However, hetero‐ and homochirally elongated oligomers were merged into the membrane. For the heterochiral constructs, a clear collision was found between the helices of oligomer **7** building the membrane segment and the investigated compound shown in Figure 2 b. This interaction hinders and blocks the heterochiral chain elongation, and, consequently, the formation of homochiral oligomers is favored.

We can conclude that the biological homochirality as a property is also occurring with unnatural compounds, like β‐peptide foldamers. The phenomenon was investigated by means of diastereoselective amino‐acid coupling. β‐Peptide oligomers composed of either *cis* or *trans* alicyclic β‐amino acids showed a tendency towards the homochiral constructs. The oligomers composed of [1*R*,2*R*]‐*trans*‐ACHC residues showed an interesting property. In non‐aqueous solvent systems, these compounds favored the formation of the heterochiral construct; whereas, in the presence of water, the opposite homochiral oligomers were preferred. Theoretical calculations indicated that water plays a crucial role in this phenomenon through the induction of self‐association. In all cases, water enhanced the diasetereoselectivity towards the homochiral oligomers. This property was observed for the l‐Leu‐containing reference α‐peptides, further underlining the key role of water in the induction of biological homochirality. It should be noted that a relationship between the chain length and diastereoselectivity of the chain‐elongation reaction might be established in the presence of water. In the case of oligomers composed of *trans*‐ACPC, *trans*‐ACHC, or l‐Leu residues, the longer chain length provided higher *DE* values in the presence of water. For the strand‐forming compounds containing *cis*‐ACHC residues, in contrast, the chain length did not drastically influence diastereoselectivity. The results gained in this fundamental study might provide the basis for industrial synthesis of β‐peptides.

## Experimental Section

### Peptide Synthesis

Homooligomer foldamers **1**–**16** were synthesized by using a standard solid‐phase technique utilizing fluorenylmethyloxycarbonyl (Fmoc) chemistry. The peptide chains were elongated on TentaGel R RAM resin (0.19 mmol g^−1^) and the syntheses were carried out manually on a 0.1 mmol scale. Couplings were performed with HATU/DIPEA without difficulties. The formed peptide sequences were cleaved from the resin with 95 % trifluoroacetic acid (TFA) and 5 % H_2_O at room temperature for 3 h. TFA was then removed and the resulting free peptides were solubilized in aqueous acetic acid (10 %), filtered, and lyophilized. The crude peptides were investigated by using HPLC–MS.

### Diastereodiscriminative Coupling Reactions

In an illustrative procedure, solutions of peptides **1**–**16** (0.01 mmol) were prepared separately with HOBt (0.12 mmol) and DIC (0.12 mmol) in CH_2_Cl_2_/DMF (2:1) in the absence or in the presence of water (2:1:1). Then Boc‐protected racemic amino acids (0.1 mmol) were added. The mixtures were stirred for 48 h, CH_2_Cl_2_ was removed by evaporation, and water was subsequently added to the residue by liophilization. The product was treated with 95 % TFA and 5 % water to remove Boc protecting groups. The solutions were then stirred for 30 min, TFA was removed in vacuo, the residue was diluted with water, and then lyophilized. Samples were analyzed by using HPLC–MS.

## Conflict of interest

*The authors declare no conflict of interest*.

## Supporting information

As a service to our authors and readers, this journal provides supporting information supplied by the authors. Such materials are peer reviewed and may be re‐organized for online delivery, but are not copy‐edited or typeset. Technical support issues arising from supporting information (other than missing files) should be addressed to the authors.

Supplementary
