# Supplementary material for: Homochirality of β‐Peptides: A Significant Biomimetic Property of Unnatural Systems
Source: ChemistryOpen. 2017 Jul 20;6(4):492–6. doi: 10.1002/open.201700078 (PMC5542748; doi:10.1002/open.201700078)
Supplement: Supplementary file 1 — Supplementary [file OPEN-6-492-s001.pdf]

ChemistryOPEN

Supporting Information

© 2017 The Authors. Published by Wiley-VCH Verlag GmbH & Co. KGaA, Weinheim

Homochirality of β-Peptides: A Significant Biomimetic Property of Unnatural Systems

István M. Mándity,[a] Imane Nekkaa,[a] Gábor Paragi,[b] and Ferenc Fülöp*[a, c]

open_201700078_sm_miscellaneous_information.pdf

SUPPORTING INFORMATION

TABLE OF CONTENTS

Experimental section **S5-S6**

Table S1. HPLC-MS chromatogram and mass spectrum data for the diastereoselective chain elongation of **1-4** in the absence and the presence of water. **S7**

Table S2. HPLC-MS chromatogram and mass spectrum data for the diastereoselective chain elongation of **5-8** in the absence and the presence of water. **S7**

Table S3. HPLC-MS chromatogram and mass spectrum data for the diastereoselective chain elongation of **9-12** in the absence and the presence of water. **S8**

Table S4. HPLC-MS chromatogram and mass spectrum data for the diastereoselective chain elongation of **13-16** in the absence and the presence of water. **S8**

Figure S1. HPLC-MS chromatogram and mass spectrum of **1**. **S9**

Figure S2. HPLC-MS chromatogram and mass spectrum of **2**. **S10**

Figure S3. HPLC-MS chromatogram and mass spectrum of **3**. **S11**

Figure S4. HPLC-MS chromatogram and mass spectrum of **4**. **S12**

Figure S5. HPLC-MS chromatogram and mass spectrum for the diastereoselective chain elongation of **1** in the absence of water. **S13**

Figure S6. HPLC-MS chromatogram and mass spectrum for the diastereoselective chain elongation of **1** in the presence of water. **S14**

Figure S7. HPLC-MS chromatogram and mass spectrum for the diastereoselective chain elongation of **2** in the absence of water. **S15**

Figure S8. HPLC-MS chromatogram and mass spectrum for the diastereoselective chain elongation of **2** in the presence of water. **S16**

Figure S9. HPLC-MS chromatogram and mass spectrum for the diastereoselective chain elongation of **3** in the absence of water. **S17**

Figure S10. HPLC-MS chromatogram and mass spectrum for the diastereoselective chain elongation of **3** in the presence of water. **S18**

Figure S11. HPLC-MS chromatogram and mass spectrum for the diastereoselective chain elongation of **4** in the absence of water. **S19**

Figure S12. HPLC-MS chromatogram and mass spectrum for the diastereoselective chain elongation of **4** in the presence of water. **S20**

Figure S13. HPLC-MS chromatogram and mass spectrum of **5**. **S21**

Figure S14. HPLC-MS chromatogram and mass spectrum of **6**. **S22**

Figure S15. HPLC-MS chromatogram and mass spectrum of **7**. **S23**

Figure S16. HPLC-MS chromatogram and mass spectrum of **8**. **S24**

Figure S17. HPLC-MS chromatogram and mass spectrum for the diastereoselective chain elongation of **5** in the absence of water. **S25**

Figure S18. HPLC-MS chromatogram and mass spectrum for the diastereoselective chain elongation of **5** in the presence of water. **S26**

Figure S19. HPLC-MS chromatogram and mass spectrum for the diastereoselective chain elongation of **6** in the absence of water. **S27**

Figure S20. HPLC-MS chromatogram and mass spectrum for the diastereoselective chain elongation of **6** in the presence of water. **S28**

Figure S21. HPLC-MS chromatogram and mass spectrum for the diastereoselective chain elongation of **7** in the absence of water. **S29**

Figure S22. HPLC-MS chromatogram and mass spectrum for the diastereoselective chain elongation of **7** in the presence of water. **S30**

Figure S23. HPLC-MS chromatogram and mass spectrum for the diastereoselective chain elongation of **8** in the absence of water. **S31**

Figure S24. HPLC-MS chromatogram and mass spectrum for the diastereoselective chain elongation of **8** in the presence of water. **S32**

Figure S25. HPLC-MS chromatogram and mass spectrum of **9**. **S33**

Figure S26. HPLC-MS chromatogram and mass spectrum of **10**. **S34**

Figure S27. HPLC-MS chromatogram and mass spectrum of **11**. **S35**

Figure S28. HPLC-MS chromatogram and mass spectrum of **12**. **S36**

Figure S29. HPLC-MS chromatogram and mass spectrum for the diastereoselective chain elongation of **9** in the absence of water. **S37**

Figure S30. HPLC-MS chromatogram and mass spectrum for the diastereoselective chain elongation of **9** in the presence of water. **S38**

Figure S31. HPLC-MS chromatogram and mass spectrum for the diastereoselective chain elongation of **10** in the absence of water. **S39**

Figure S32. HPLC-MS chromatogram and mass spectrum for the diastereoselective chain elongation of **10** in the presence of water. **S40**

Figure S33. HPLC-MS chromatogram and mass spectrum for the diastereoselective chain elongation of **11** in the absence of water. **S41**

Figure S34. HPLC-MS chromatogram and mass spectrum for the diastereoselective chain elongation of **11** in the presence of water. **S42**

Figure S35. HPLC-MS chromatogram and mass spectrum for the diastereoselective chain elongation of **12** in the absence of water. **S43**

Figure S36. HPLC-MS chromatogram and mass spectrum for the diastereoselective chain elongation of **12** in the presence of water. **S44**

Figure S37. HPLC-MS chromatogram and mass spectrum of **13**. **S45**

Figure S38. HPLC-MS chromatogram and mass spectrum of **14**. **S46**

Figure S39. HPLC-MS chromatogram and mass spectrum of **15**. **S47**

Figure S40. HPLC-MS chromatogram and mass spectrum of **16**. **S48**

Figure S41. HPLC-MS chromatogram and mass spectrum for the diastereoselective chain elongation of **13** in the absence of water. **S49**

Figure S42. HPLC-MS chromatogram and mass spectrum for the diastereoselective chain elongation of **13** in the presence of water. **S50**

Figure S43. HPLC-MS chromatogram and mass spectrum for the diastereoselective chain elongation of **14** in the absence of water. **S51**

Figure S44. HPLC-MS chromatogram and mass spectrum for the diastereoselective chain elongation of **14** in the presence of water. **S52**

Figure S45. HPLC-MS chromatogram and mass spectrum for the diastereoselective chain elongation of **15** in the absence of water. **S53**

Figure S46. HPLC-MS chromatogram and mass spectrum for the diastereoselective chain elongation of **15** in the presence of water. **S54**

Figure S47. HPLC-MS chromatogram and mass spectrum for the diastereoselective chain elongation of **16** in the absence of water. **S55**

Figure S48. HPLC-MS chromatogram and mass spectrum for the diastereoselective chain elongation of **16** in the presence of water. **S56**

Table S5-12 Optimized cartesian coordinates (in Å) and ADF total bonding energies (in kcal/mol) of the optimized minimum or transition state geometries calculated at OLYP/TZ2P level of theory in chloroform or water solvent. **S57**

References **S66**

Experimental Section

Peptide Synthesis: Homooligomer foldamers **1–16** were synthesized by using a standard solid-phase technique involving 9H-fluoren-9-ylmethoxycarbonyl (Fmoc) chemistry with chain-lengths of the oligomers varying between 3-6 units. The peptide chains were elongated on TentaGel R RAM resin (0.19 mmol.g⁻1) and the syntheses were carried out manually on a 0.1 mmol scale. Couplings were performed with HATU/DIPEA {HATU = [2-(7-aza-1H-benzotriazol-1-yl]- 1,1,3,3-tetramethyluronium hexafluorophosphate, DIPEA = N,Ndiisopropylethylamine} without difficulties. The formed peptide sequences were cleaved from the resin with 95% trifluoroacetic acid (TFA) and 5% H_2O at room temperature for 3 h. TFA was then removed and the resulting free peptides were solubilized in aqueous AcOH (10%), filtered and lyophilized. The crude peptides were investigated by HPLC-MS.

Diastereodiscriminative coupling reactions: In an illustrative procedure, solutions of peptides **1-16** (0.01 mmol) were prepared separately with HOBt {HOBt = 1-hydroxybenzotriazole} (0.12 mmol) and DIC {DIC = N,N′-Diisopropylcarbodiimide} (0.12mmol) in CH_2Cl_2/DMF (2:1) in the absence or in the presence of water (2:1:1). Then Boc protected racemic amino acids (0.1 mmol) were added. The mixtures were stirred for 48 h, CH_2Cl_2 was removed by evaporation, water was added to the residue followed by liophilization. The product was treated with 95% trifluoroacetic acid (TFA) and 5% water to remove Boc protecting groups. The solutions were then stirred for 30 min, TFA was removed in vacuo, the residue was diluted with water and then lyophilized. Samples were analyzed by HPLC-MS.

HPLC-MS: HPLC-MS measurements were performed with a Phenomenex 3.6u XB-C18 column (250×4.60 mm). The solvent system was consisted of AcOH (0.1%) in water (A), AcOH (0.1%) in ACN (B); gradient: 5%–80% B over 35 min, at a flow rate of 1 mL min^{-1}. Chromatograms and spectra were recorded in positive ionization mode. The following retention times and molecular weights were determined: **a**: {**1**, m=2, RT= 6.06 , m/z = 351 $[M + H]^+$; **2**, m=3, RT= 8.07, m/z = 462 $[M + H]^+$; **3**, m=4, RT= 10.51, m/z = 573 $[M + H]^+$; **4**, m=5, RT= 12.79, m/z = 684$[M + H]^+$}; **b**: {**5**, n=2, RT= 7.14, m/z =393 $[M + H]^+$; **6**, n=3, RT=10.33 , m/z = 518 $[M + H]^+$; **7**, n=4, RT=13.27, m/z = 643 $[M + H]^+$; **8**, n=5, RT=15.08 , m/z = 768 $[M + H]^+$}; **c**: {**9**, o=2, RT= 7.27, m/z = 393 $[M + H]^+$; **10**, o=3, RT= 9.63, m/z =518 $[M + H]^+$;**11**, o=4, RT= 11.4, m/z = 643 $[M + H]^+$; **12**, o=5, RT= 13.03, m/z = 768 $[M + H]^+$ }; **d**: {**13**, p=2, RT= 9.60, m/z = 357 $[M + H]^+$;**14**,

p=3, RT= 12.73, m/z = 470 [M + H]$^+$;**15**, p=4, RT=15.13, m/z = 583 [M + H]$^+$;**16**, p=5, RT= 17.67, m/z = 696 [M + H]$^+$ }.

DFT computations: All calculations were performed with the Amsterdam Density Functional program suit (ADF) developed by E.J. Barends *et al* [1] applying the OLYP functional in combination with the TZ2P basis set. The latter means that molecular orbitals were expanded in a large uncontracted set of Slater-type orbitals (STOs) containing diffuse functions (no Gaussian functions are involved).[1e] The basis set is of triple-ζ quality for all atoms and has been augmented with two sets of polarization functions. The 1s core shells of carbon, nitrogen and oxygen were treated by the frozen-core approximation.[2] An auxiliary set of s, p, d, f and g STOs was used to fit the molecular density and to represent the Coulomb and exchange potentials accurately in each self-consistent field cycle.[1b, 1f]

The OLYP functional is based on the exchange functional (OPTX) developed by Handy and Cohen[3] and the well-known Lee-Young-Parr correlation expression.[4] It was found that the overall performance of the OLYP functional is fairly good in combination with large basis set comparing to other popular (e.g. B3LYP) functionals.[5]

Solvent effects have been estimated using the conductor-like screening model[6] (COSMO) for both solvents (water and chloroform) as implemented in the ADF program.[7]

Transition states were determined by a stepwise process: First, the independent constituents (boc-protected [1R,2R] or [1S,2S]-*trans*-ACHC hydroxybenzotriazole ester as the new unit and the four-unit-long [1R,2R]-*trans*-ACHC-NH2 foldamer) were optimized in the selected solvent. Then, the new unit was moved toward the oligomer with the linear transit method to have an estimate for the transition state optimization. Throughout the linear transit the distance was decreased gradually between the C atom from the ester group of the new unit and the N of the amino group from the oligomer. The total energy of the system shows a single barrier shape as the function of the distance, and taking the geometry nearest to the top of the curve, transition state optimization was performed. Reaction barriers were calculated as the energy difference between the transition state of the complex and the sum of the total energies of the independently optimized constituents. Each transition state or minimum search was augmented with frequency calculation to verify the transition state (1 negative frequency) or minimum (no negative frequency) character of the result.

Table S1. HPLC-MS chromatogram and mass spectrum data for the diastereoselective chain elongation of **1-4** in the absence and the presence of water.

m	RT_{REF} (min)	***Absence of H_2O***				***Presence of H_2O***			
		RT_1 (min)<br>Area	RT_2 (min)<br>Area	**DS [%]**	**DE [%]**	RT_1 (min)<br>Area	RT_2 (min)<br>Area	**DS [%]**	**DE [%]**
**1**	8.06	6.29<br>63896	6.90<br>141264	**68.85**	**37.71**	6.28<br>137772	6.90<br>240538	**63.58**	**27.16**
**2**	10.51	9.88<br>18550	10.55<br>35132	**65.44**	**30.89**	9.87<br>13730	10.55<br>36529	**72.68**	**45.36**
**3**	12.79	12.55<br>2149	12.92<br>3836	**64.10**	**28.18**	12.48<br>2768	12.85<br>5876	**67.98**	**35.96**
**4**	-	14.80<br>3574	15.54<br>5139	**59.00**	**17.96**	14.78<br>882	15.57<br>2568	**74.43**	**48.87**

Table S2. HPLC-MS chromatogram and mass spectrum data for the diastereoselective chain elongation of **5-8** in the absence and the presence of water.

n	RT_{REF} (min)	***Absence of H_2O***				***Presence of H_2O***			
		RT_1 (min)<br>Area	RT_2 (min)<br>Area	**DS [%]**	**DE [%]**	RT_1 (min)<br>Area	RT_2 (min)<br>Area	**DS [%]**	**DE [%]**
**1**	10.34	9.09<br>6650	10.47<br>2587	**28.01**	**-43.98**	8.80<br>3629	10.18<br>6950	**65.7**	**31.99**
**2**	13.27	11.53<br>798	13.83<br>210	**20.57**	**-58.84**	12.06<br>3449	13.21<br>31578	**90.15**	**80.30**
**3**	15.08	13.72<br>38735	15.81<br>12370	**24.20**	**-51.58**	13.57<br>12363	15.10<br>119382	**90.62**	**81.23**
**4**	-	15.83<br>213512	18.50<br>43859	**17.04**	**-66.00**	15.68<br>102576	17.58<br>1881492	**94.83**	**89.66**

Table S3. HPLC-MS chromatogram and mass spectrum data for the diastereoselective chain elongation of **9-12** in the absence and the presence of water.

**o**	RT_{REF} (min)	***Absence of*** H_2O				***Presence of*** H_2O			
		RT_1 (min) Area	RT_2 (min) Area	**DS** [%]	**DE** [%]	RT_1 (min) Area	RT_2 (min) Area	**DS** [%]	**DE** [%]
**1**	9.62	9.56<br>416	10.98<br>840	**66.87**	**33.75**	9.59<br>824	10.98<br>1692	**67.25**	**34.50**
**2**	11.4	11.38<br>2761	12.42<br>7175	**72.21**	**44.42**	11.39<br>955	12.41<br>2176	**69.5**	**39.00**
**3**	13.03	13.01<br>1180	13.96<br>1618	**57.82**	**15.65**	12.99<br>4531	13.97<br>7214	**61.42**	**22.84**
**4**	-	14.33<br>5478	15.13<br>10803	**66.35**	**32.70**	14.33<br>7941	15.15<br>11646	**59.5**	**18.92**

Table S4. HPLC-MS chromatogram and mass spectrum data for the diastereoselective chain elongation of **13-16** in the absence and the presence of water.

**p**	RT_{REF} (min)	***Absence of*** H_2O				***Presence of*** H_2O			
		RT_1 (min) Area	RT_2 (min) Area	**DS** [%]	**DE** [%]	RT_1 (min) Area	RT_2 (min) Area	**DS** [%]	**DE** [%]
**1**	12.73	12.77<br>33632	14.25<br>31005	**52.03**	**4.06**	12.77<br>17754	14.25<br>14980	**54.23**	**8.47**
**2**	15.13	15.21<br>49182	16.76<br>32841	**59.96**	**19.92**	15.22<br>39695	16.77<br>32100	**55.3**	**10.57**
**3**	17.58	17.60<br>135370	18.88<br>69925	**65.93**	**31.87**	17.61<br>44677	18.90<br>30970	**59.06**	**18.11**
**4**	-	20.63<br>132070	22.27<br>62204	**67.98**	**35.96**	20.64<br>116070	22.33<br>48297	**70.62**	**41.23**

Figure S1. HPLC-MS chromatogram and mass spectrum of **1**.

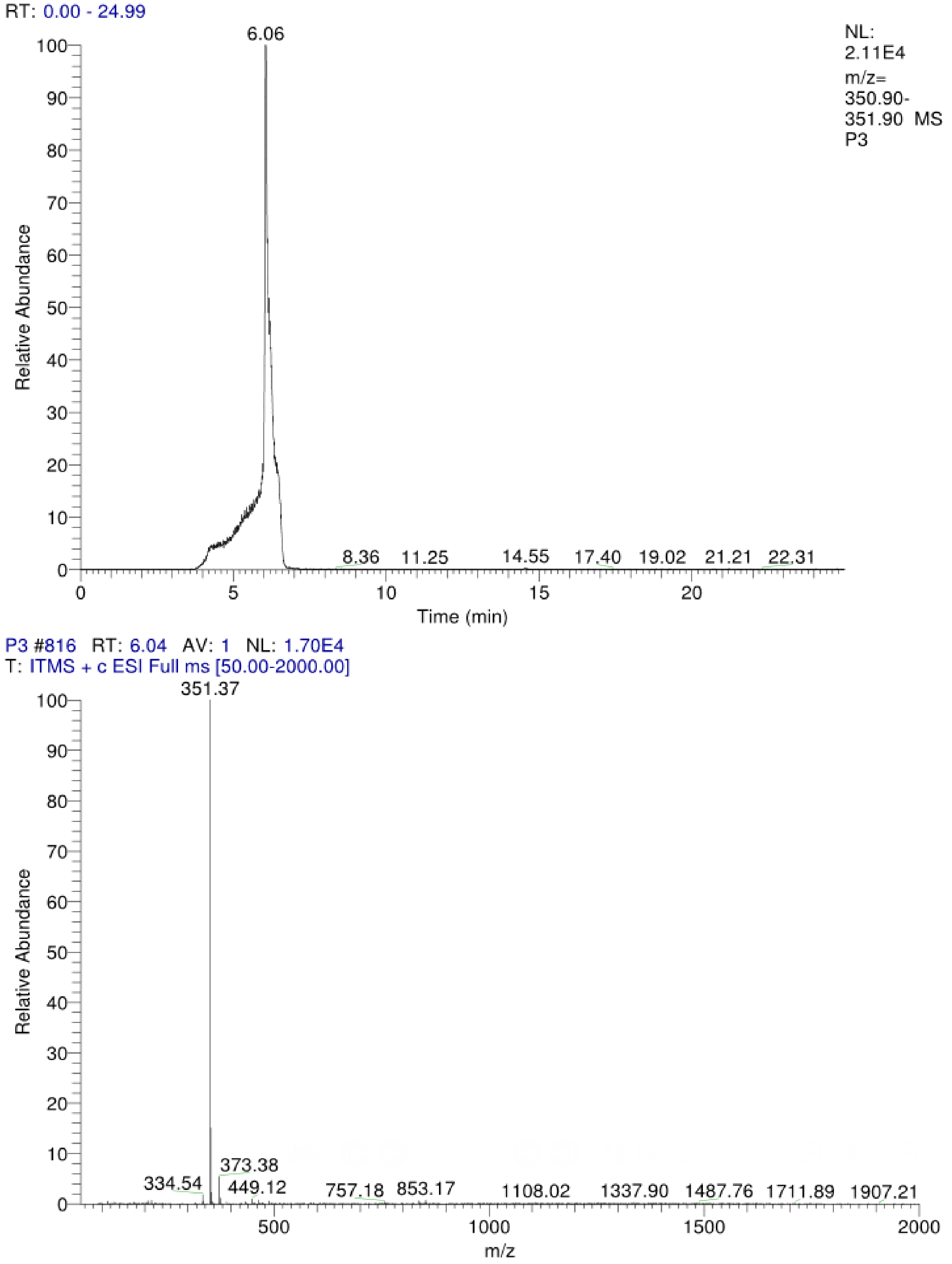

Figure S2. HPLC-MS chromatogram and mass spectrum of **2**.

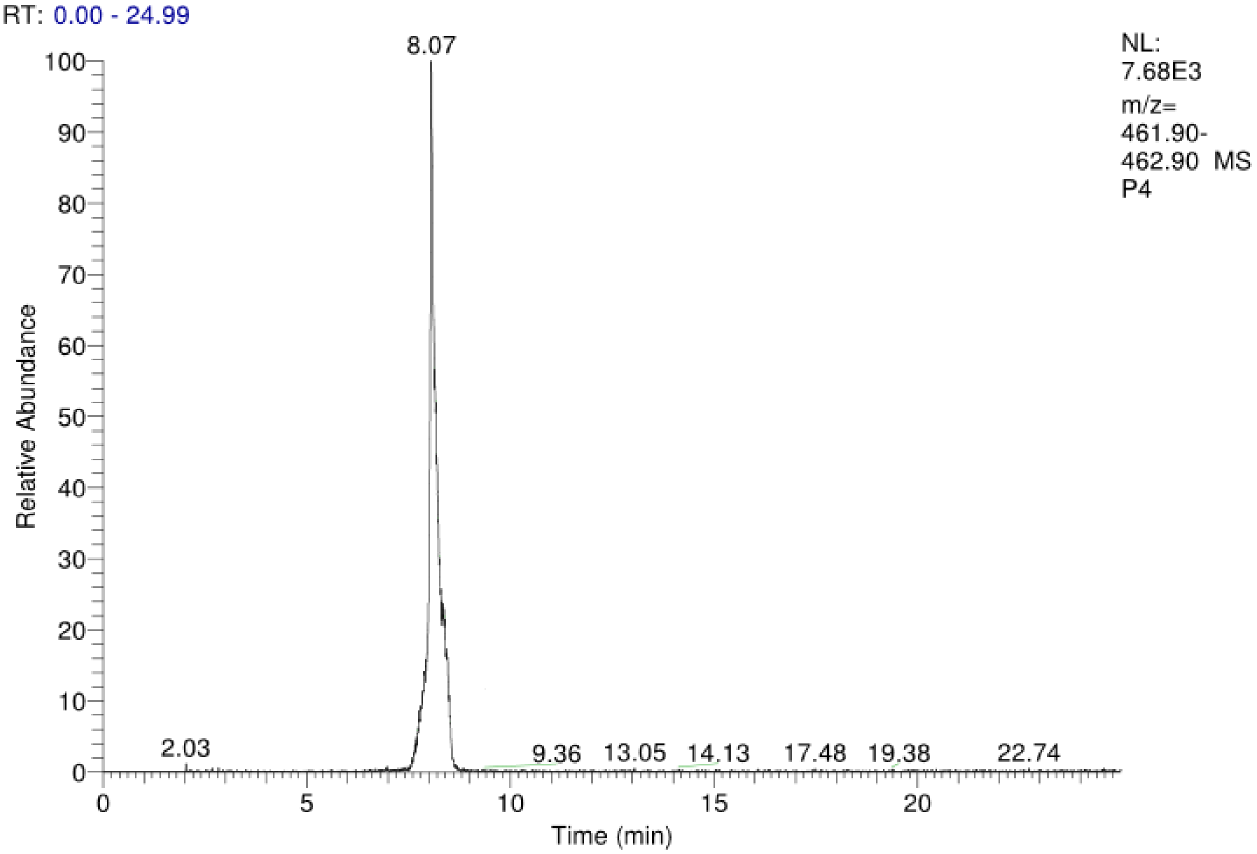

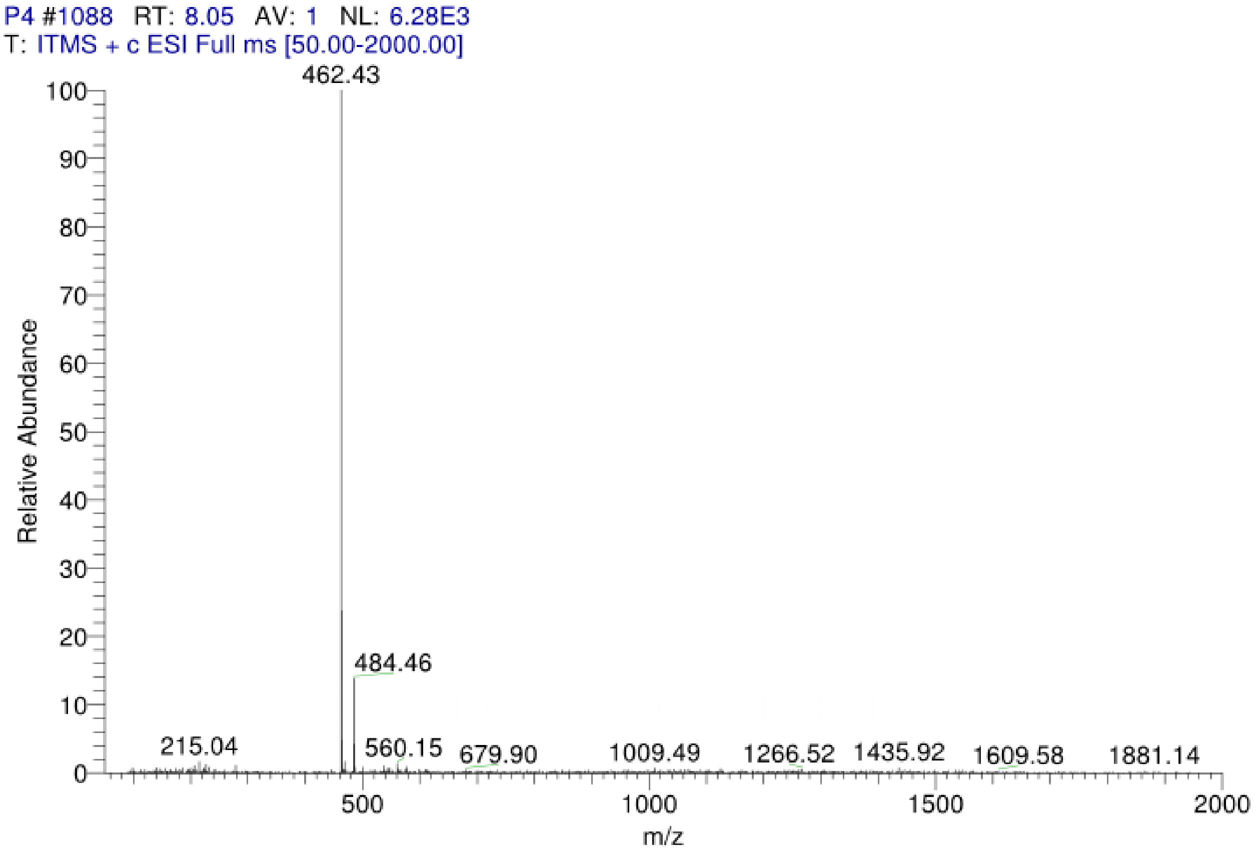

Figure S3. HPLC-MS chromatogram and mass spectrum of **3**.

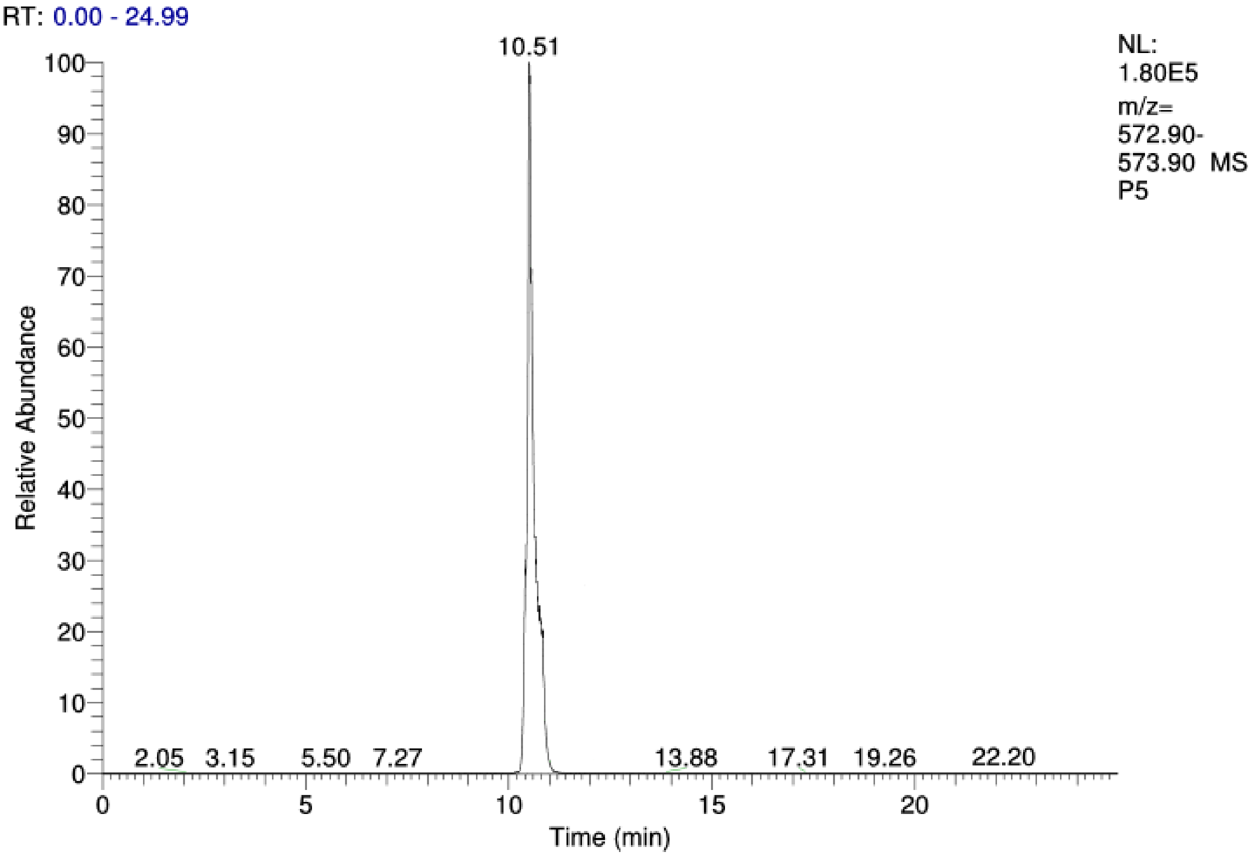

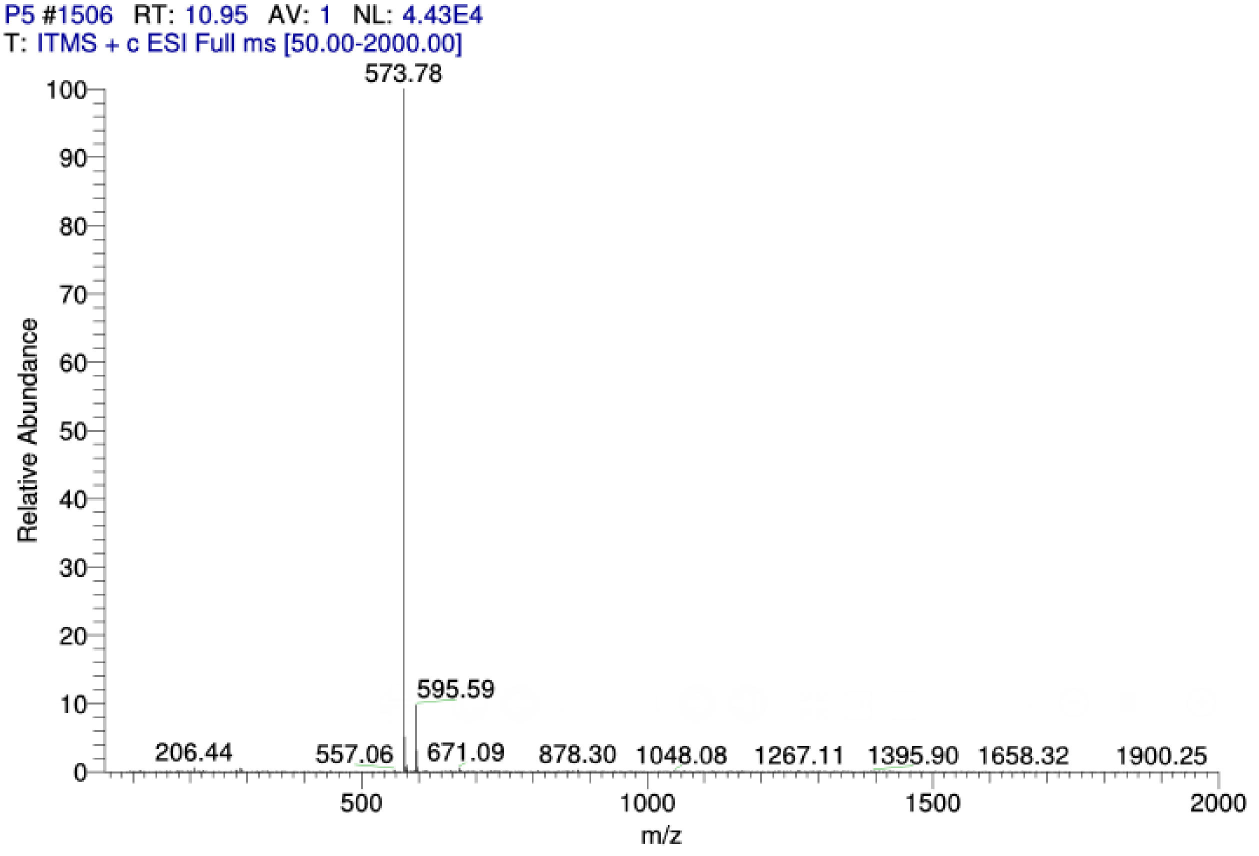

Figure S4. HPLC-MS chromatogram and mass spectrum of **4**.

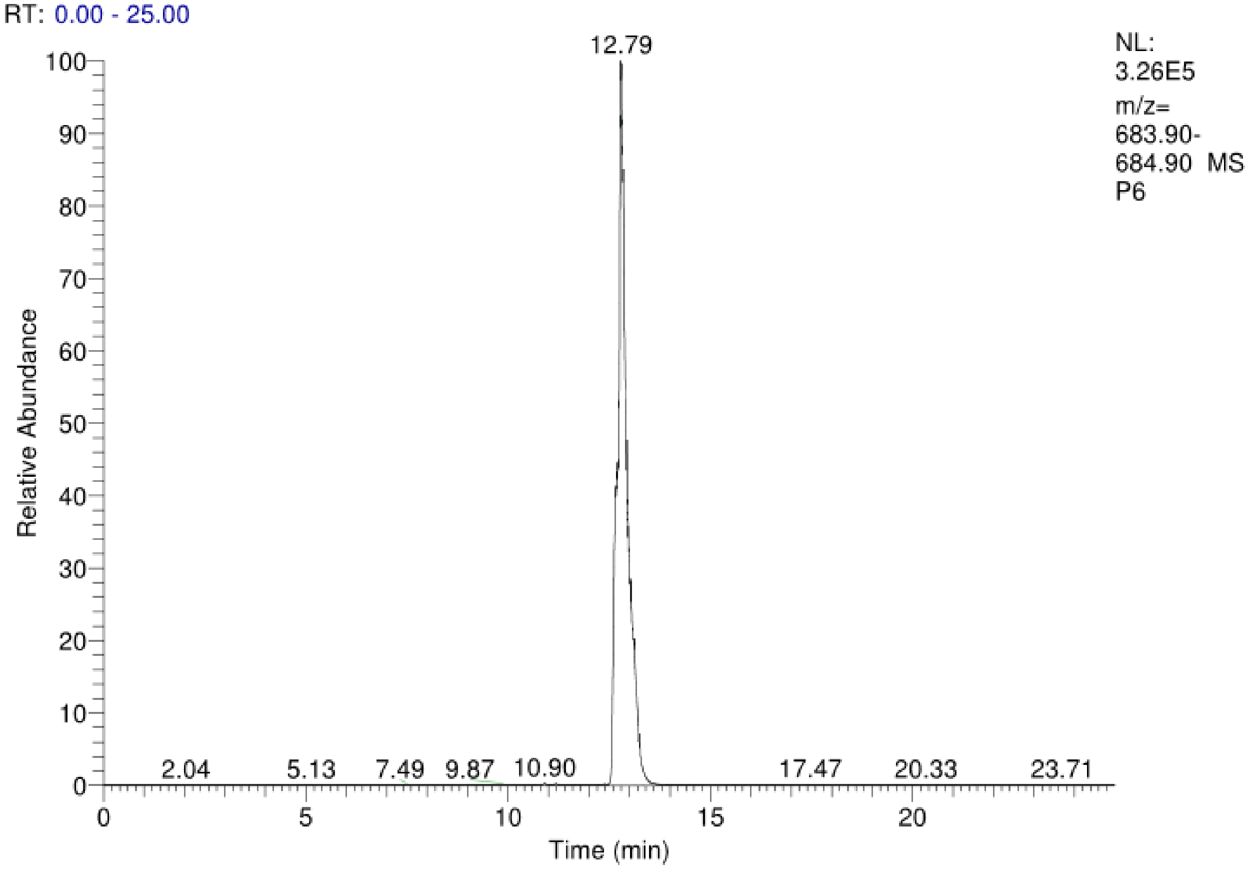

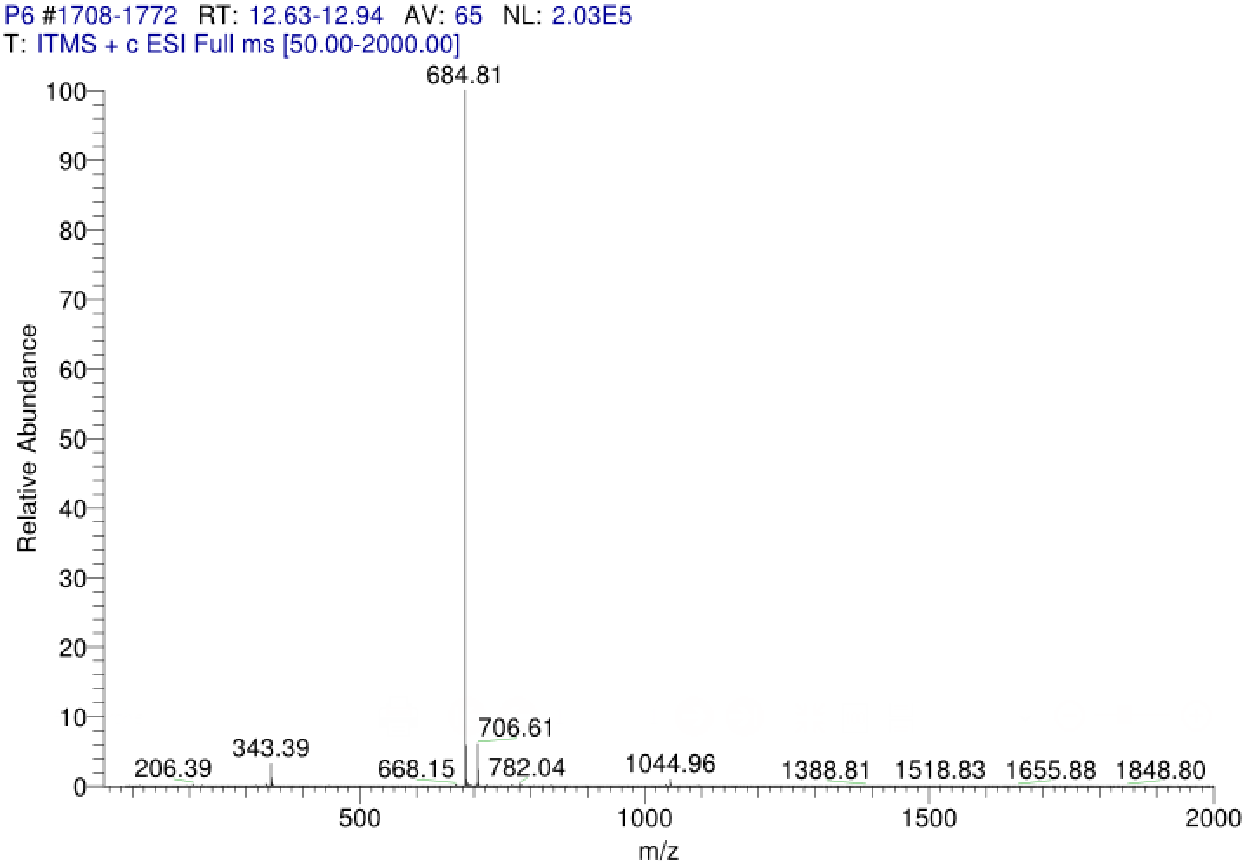

Figure S5. HPLC-MS chromatogram and mass spectrum for the diastereoselective chain elongation of **1** in the absence of water.

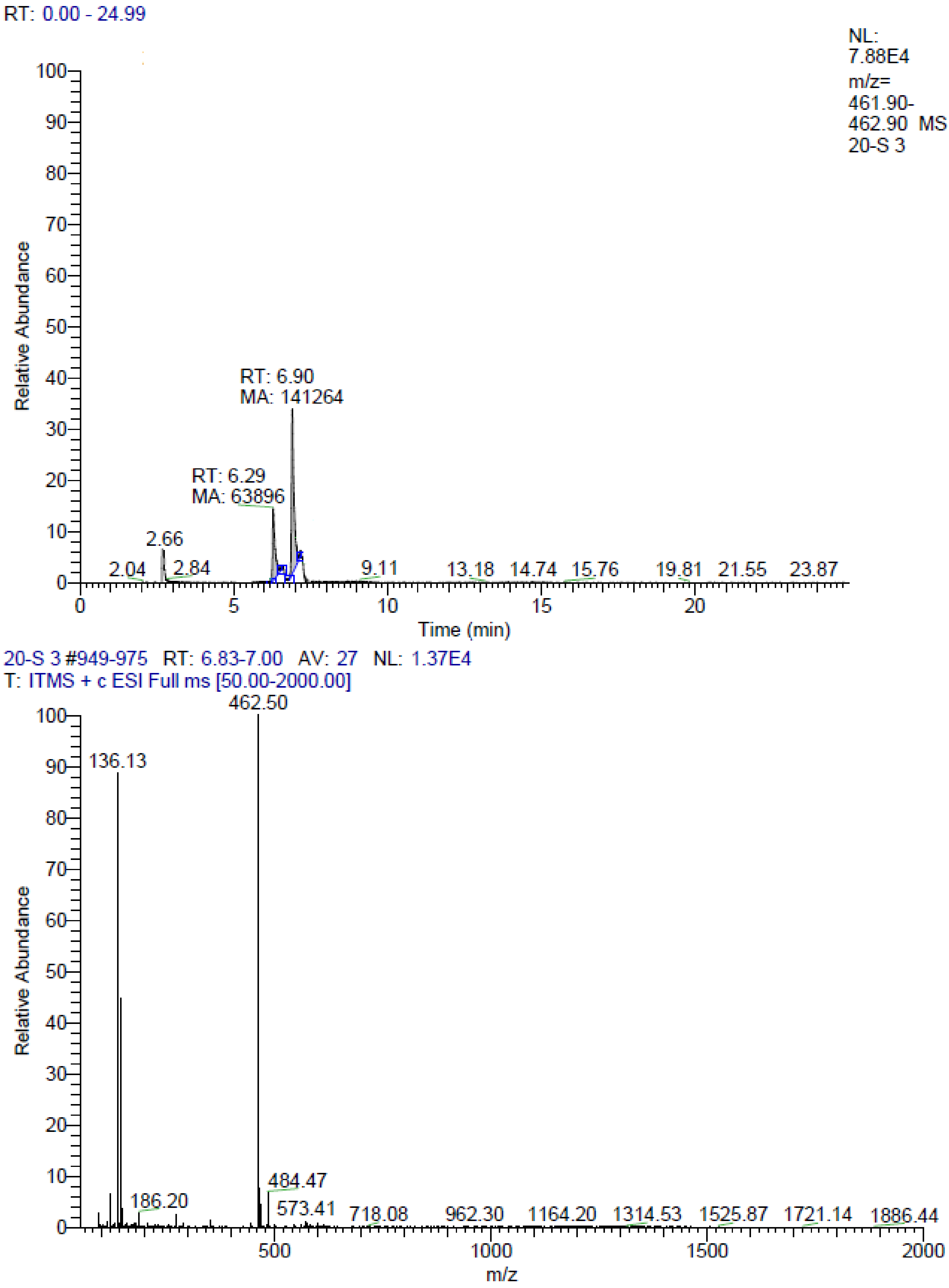

Figure S6. HPLC-MS chromatogram and mass spectrum for the diastereoselective chain elongation of **1** in the presence of water.

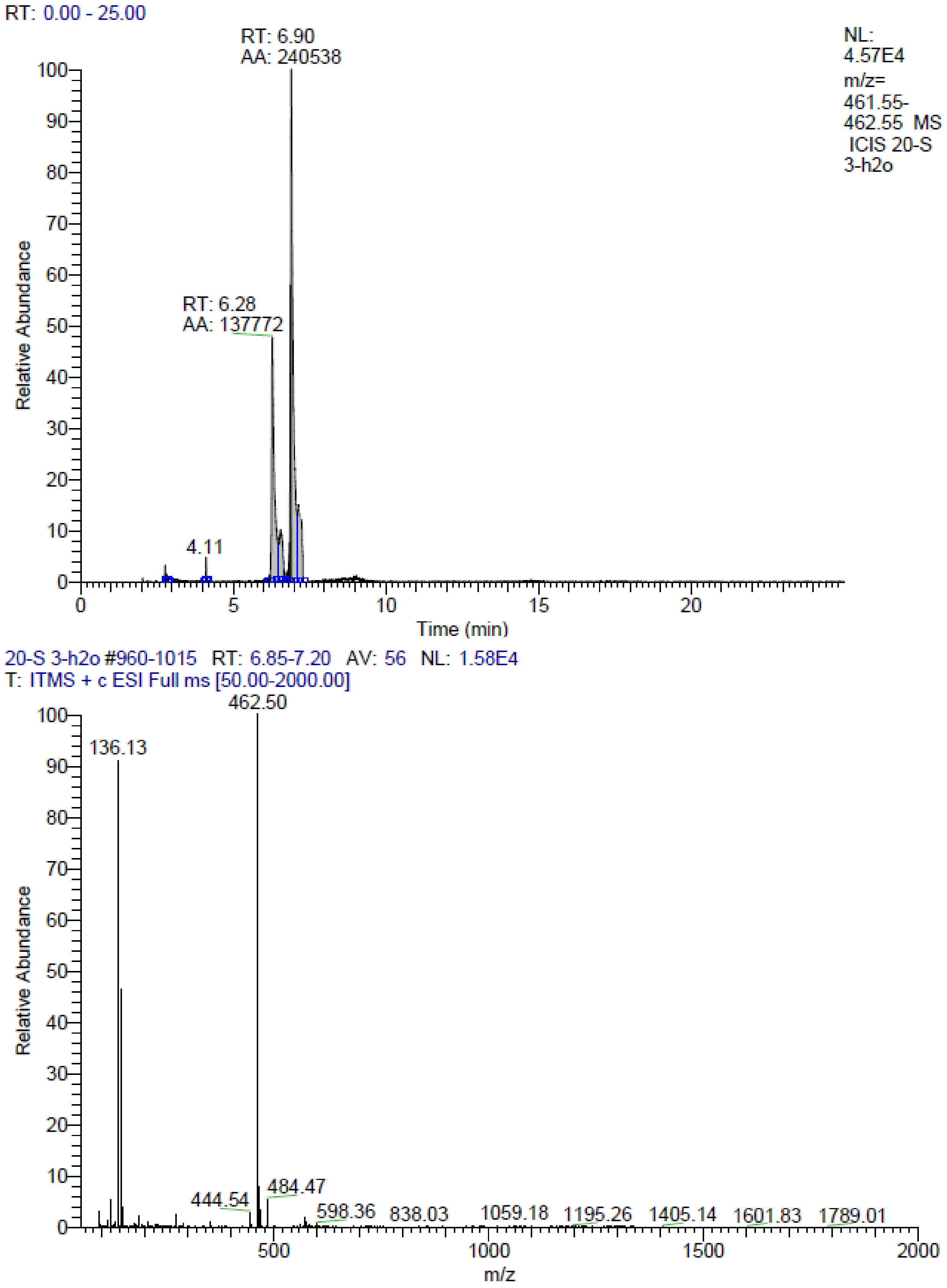

Figure S7. HPLC-MS chromatogram and mass spectrum for the diastereoselective chain elongation of **2** in the absence of water.

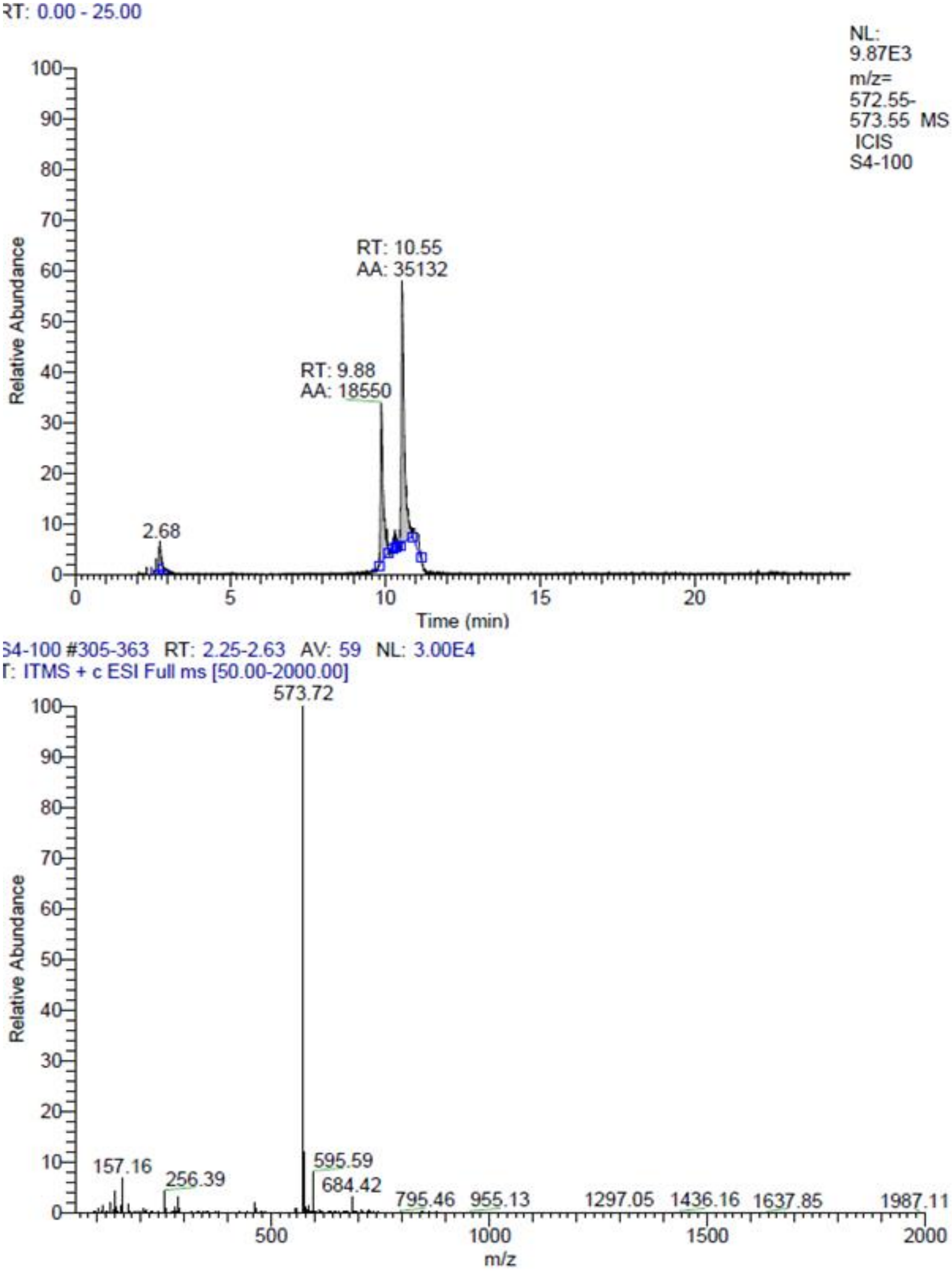

Figure S8. HPLC-MS chromatogram and mass spectrum for the diastereoselective chain elongation of **2** in the presence of water.

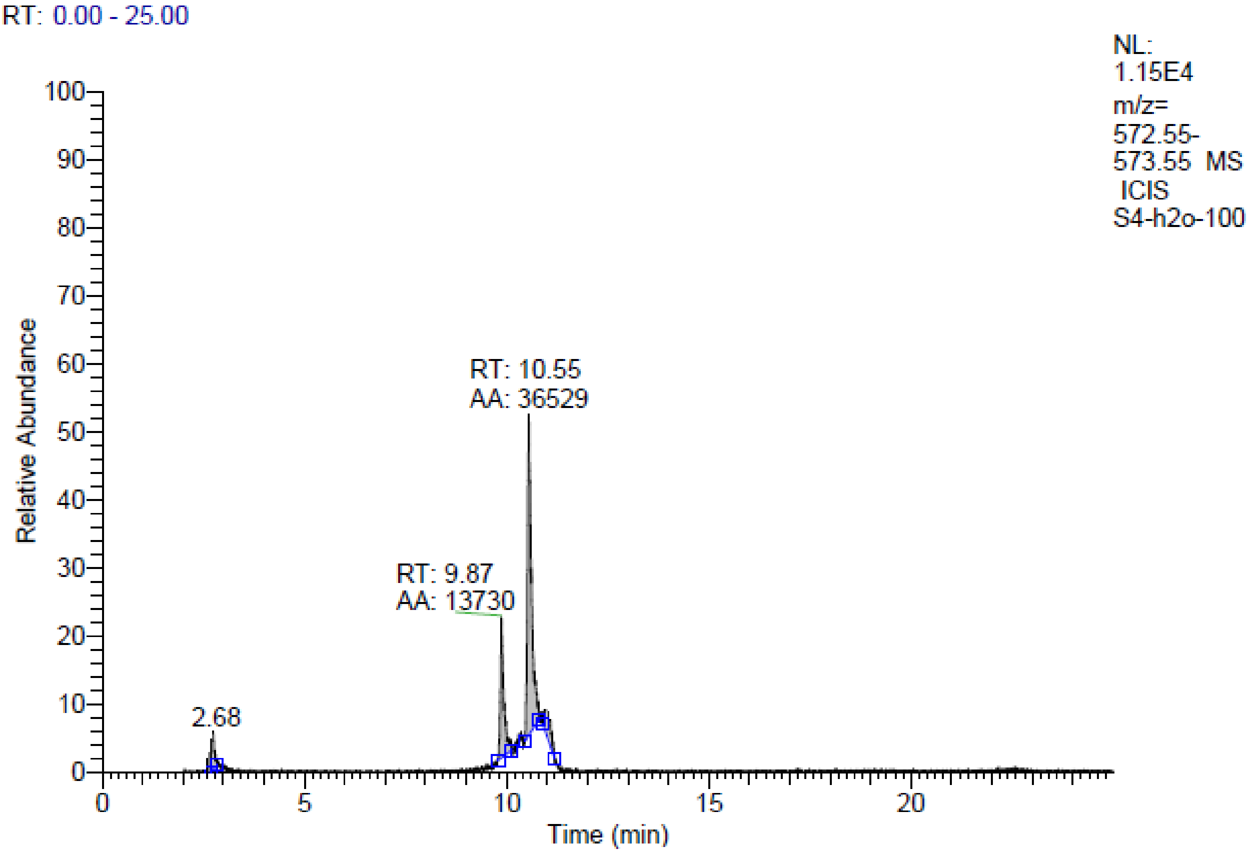

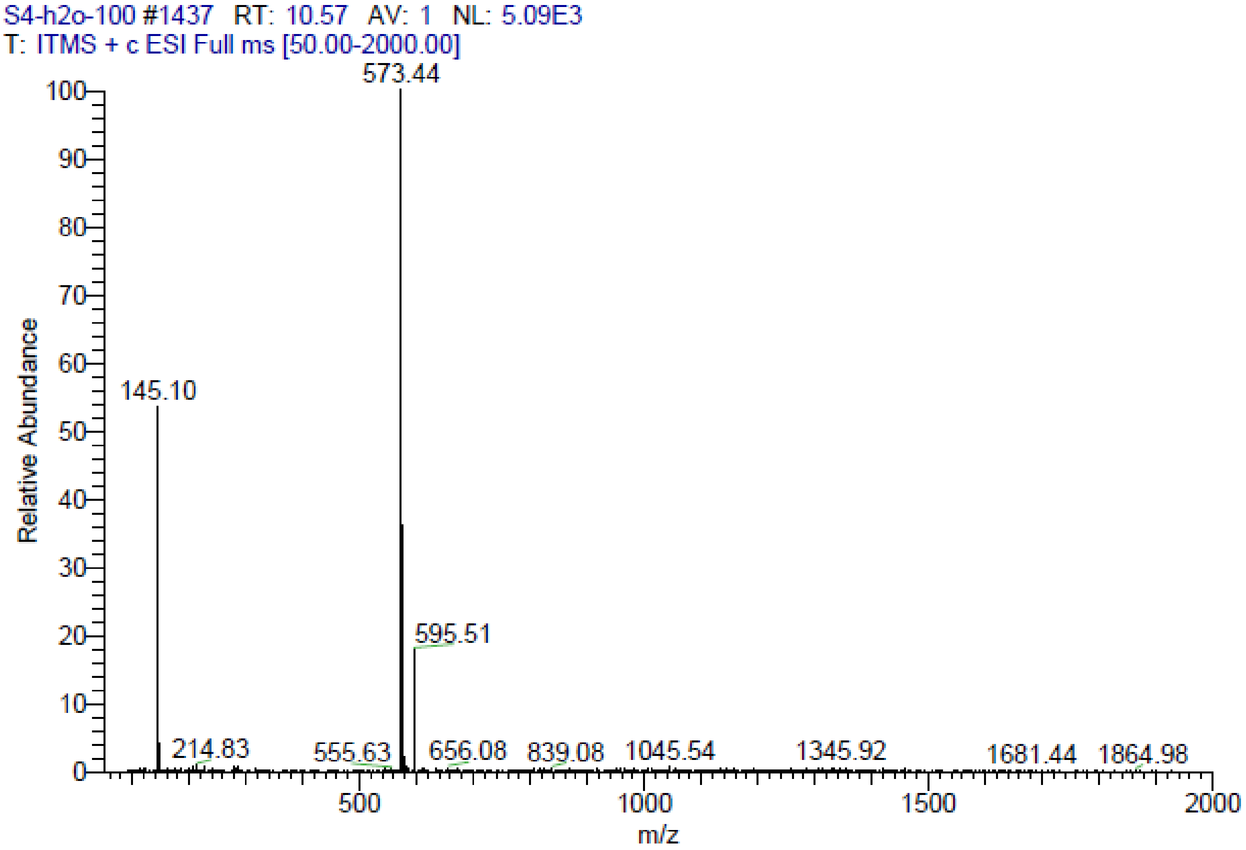

Figure S9. HPLC-MS chromatogram and mass spectrum for the diastereoselective chain elongation of **3** in the absence of water.

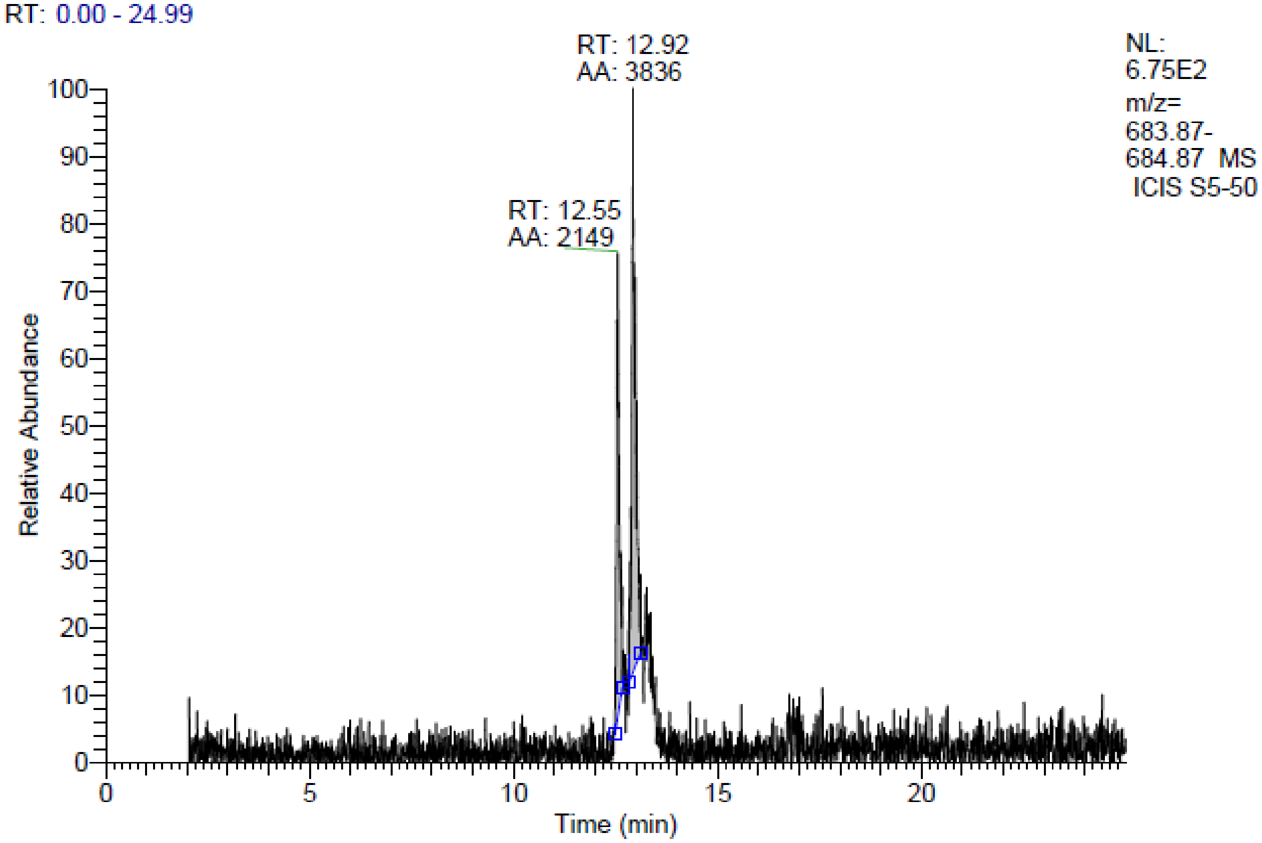

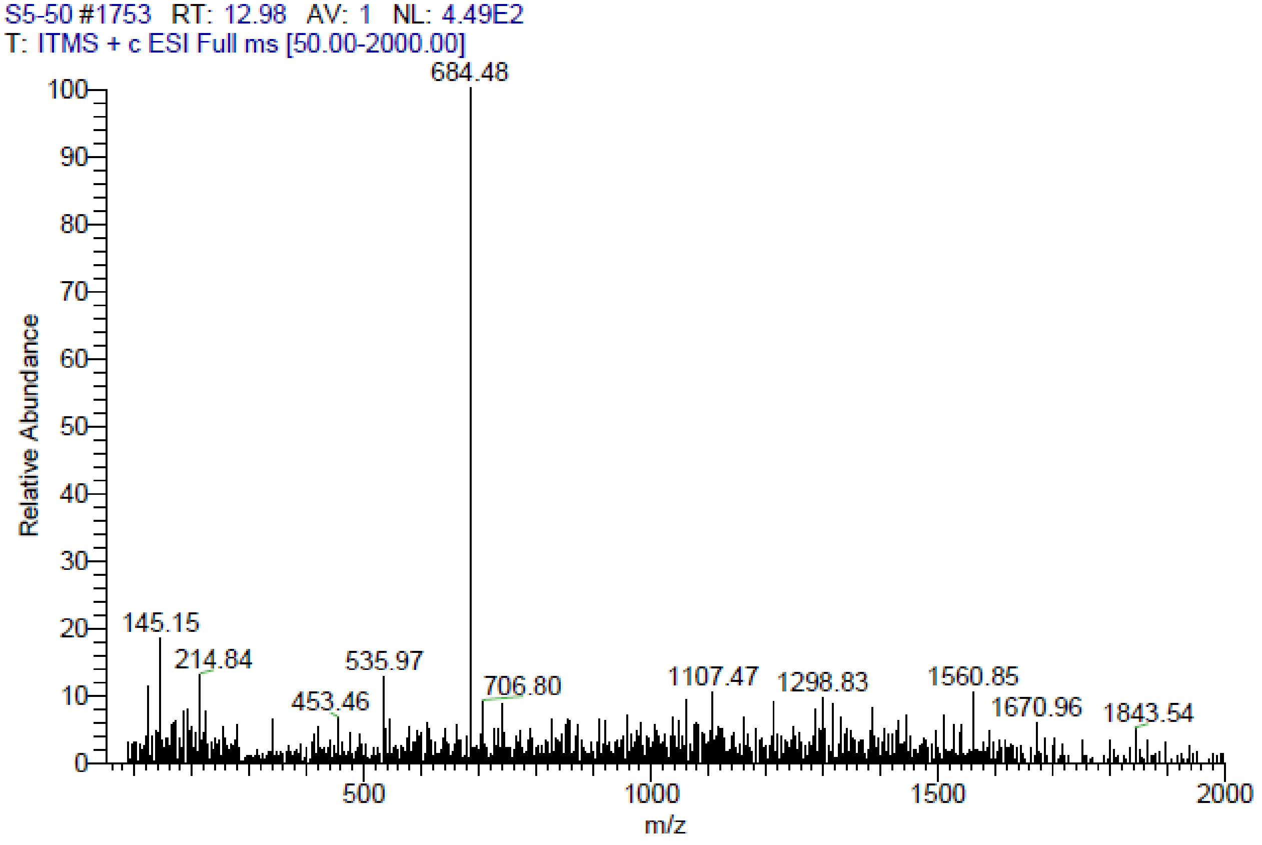

Figure S10. HPLC-MS chromatogram and mass spectrum for the diastereoselective chain elongation of **3** in the presence of water.

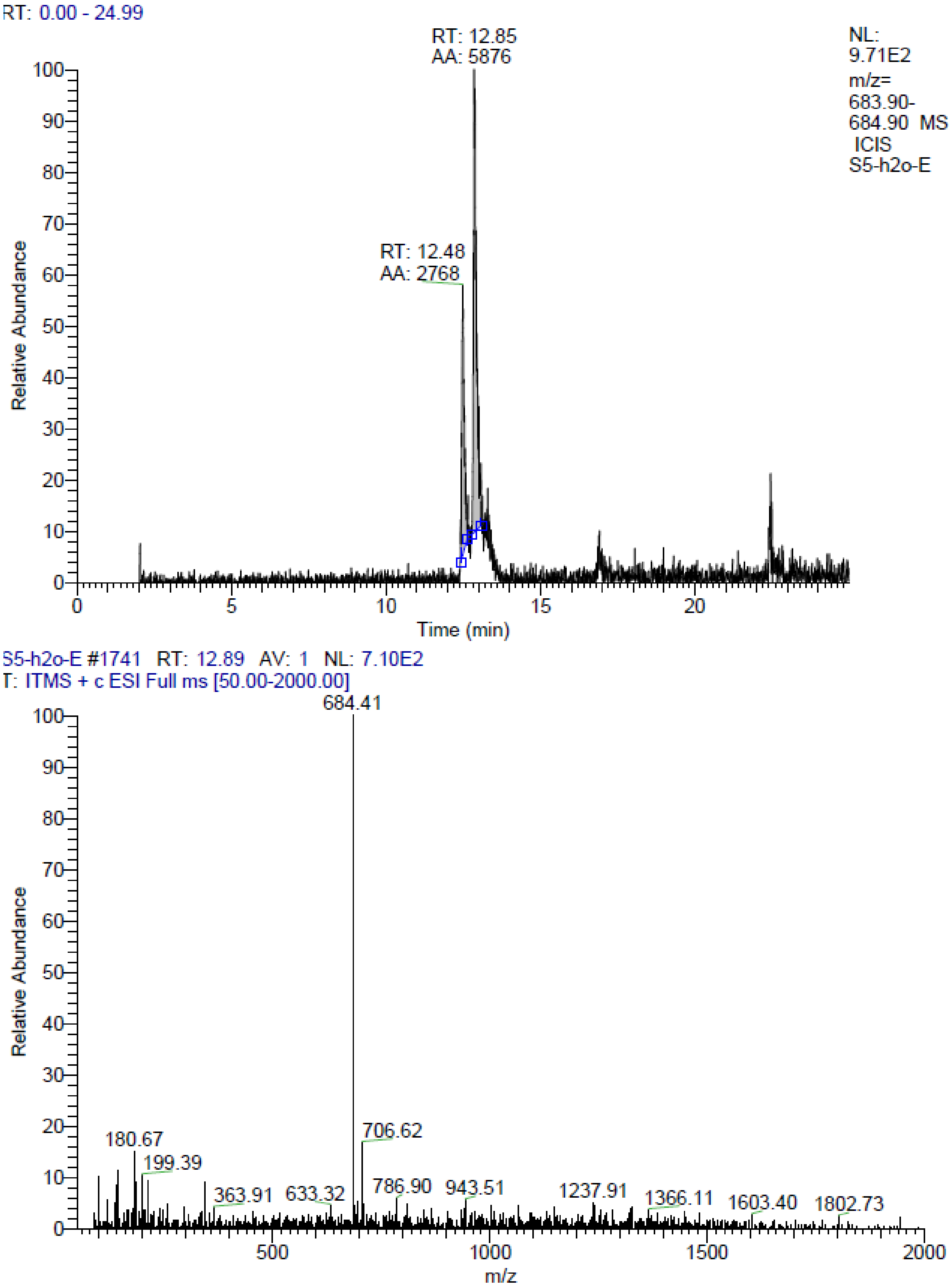

Figure S11. HPLC-MS chromatogram and mass spectrum for the diastereoselective chain elongation of **4** in the absence of water.

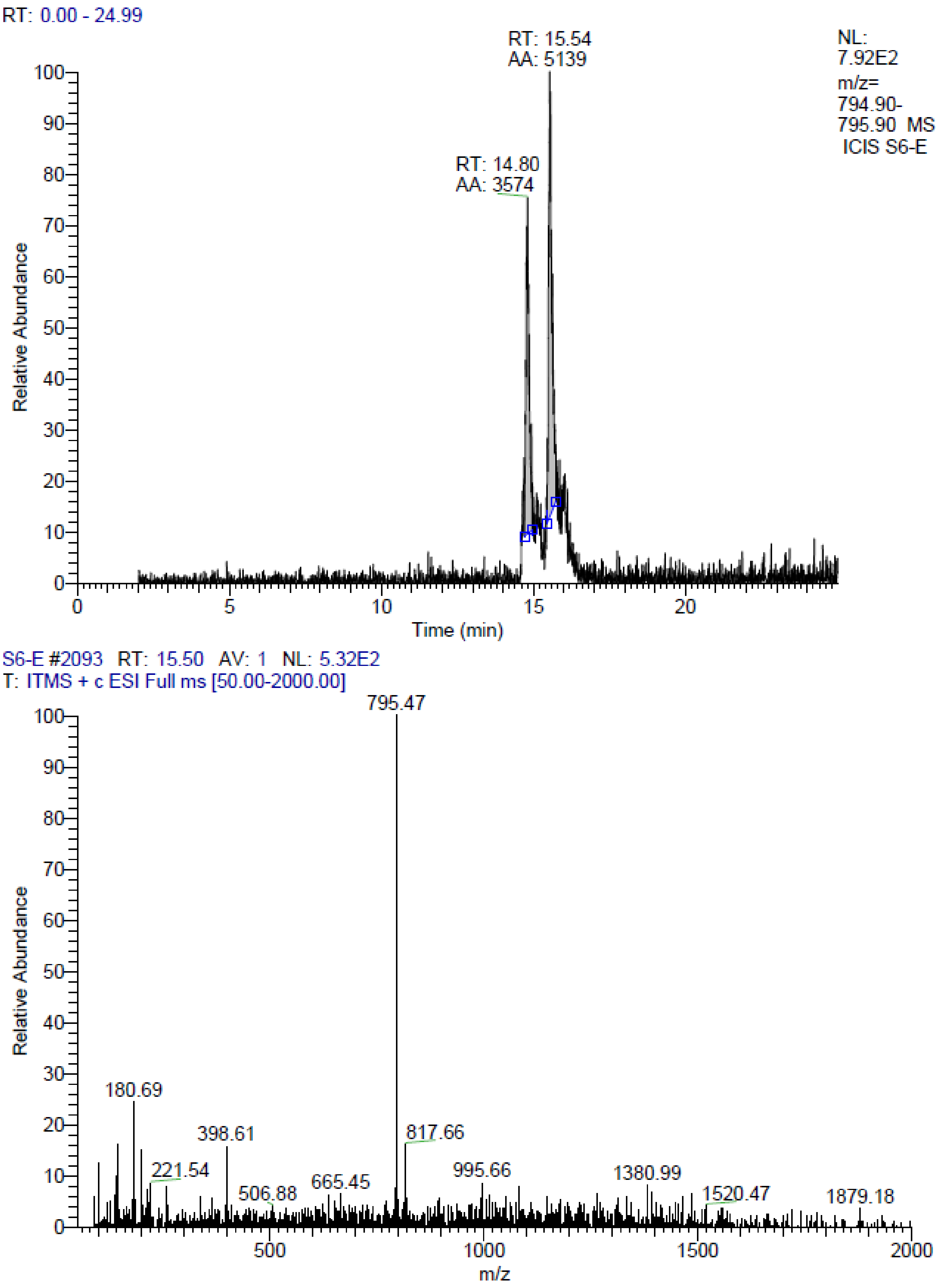

Figure S12. HPLC-MS chromatogram and mass spectrum for the diastereoselective chain elongation of **4** in the presence of water.

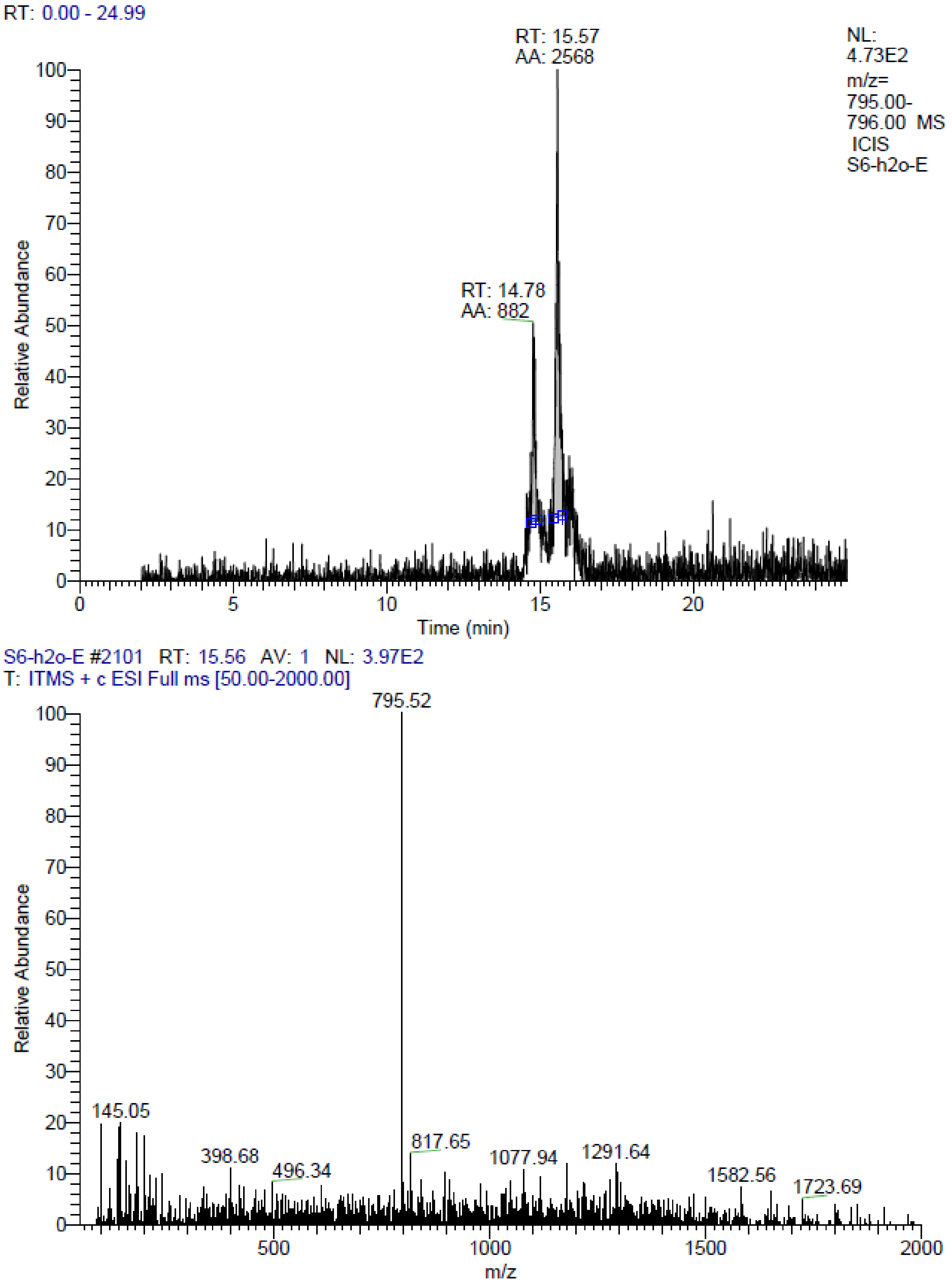

Figure S13. HPLC-MS chromatogram and mass spectrum of **5**.

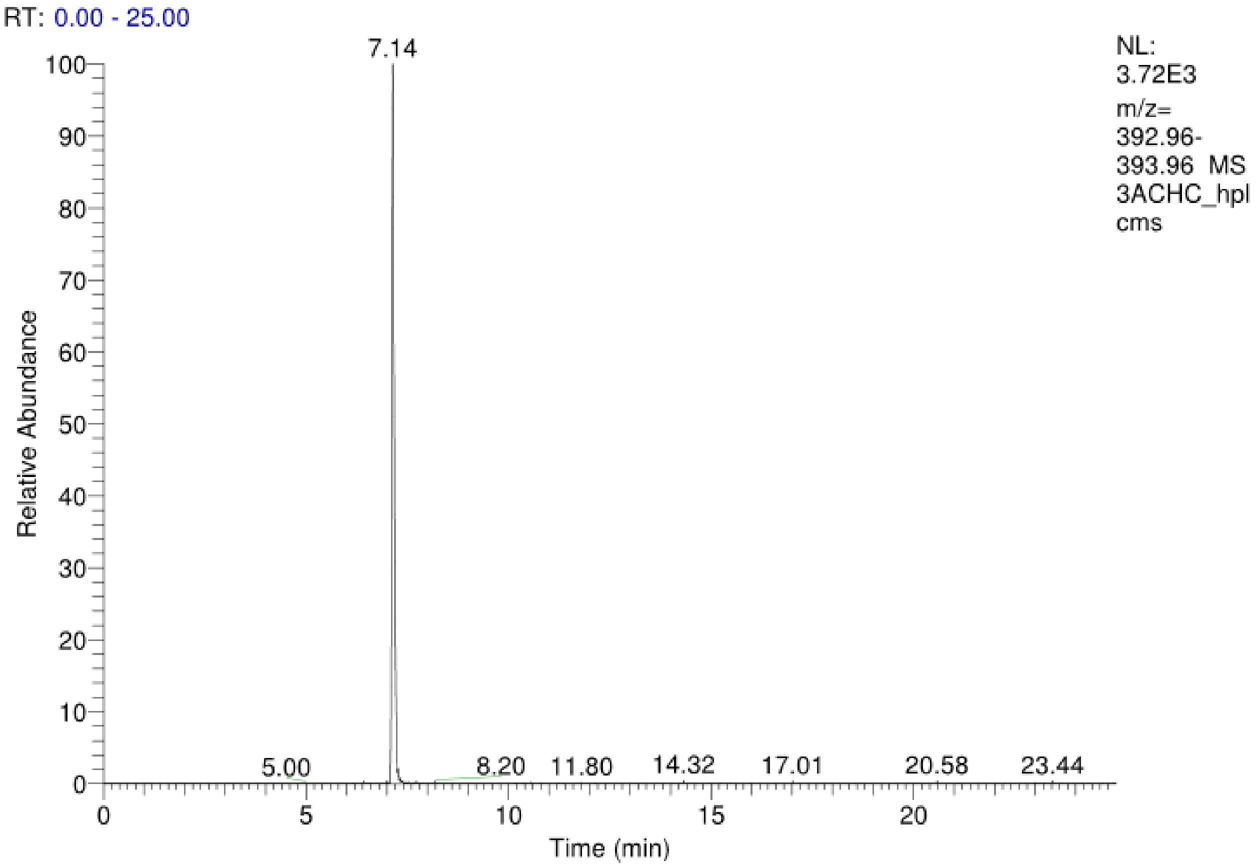

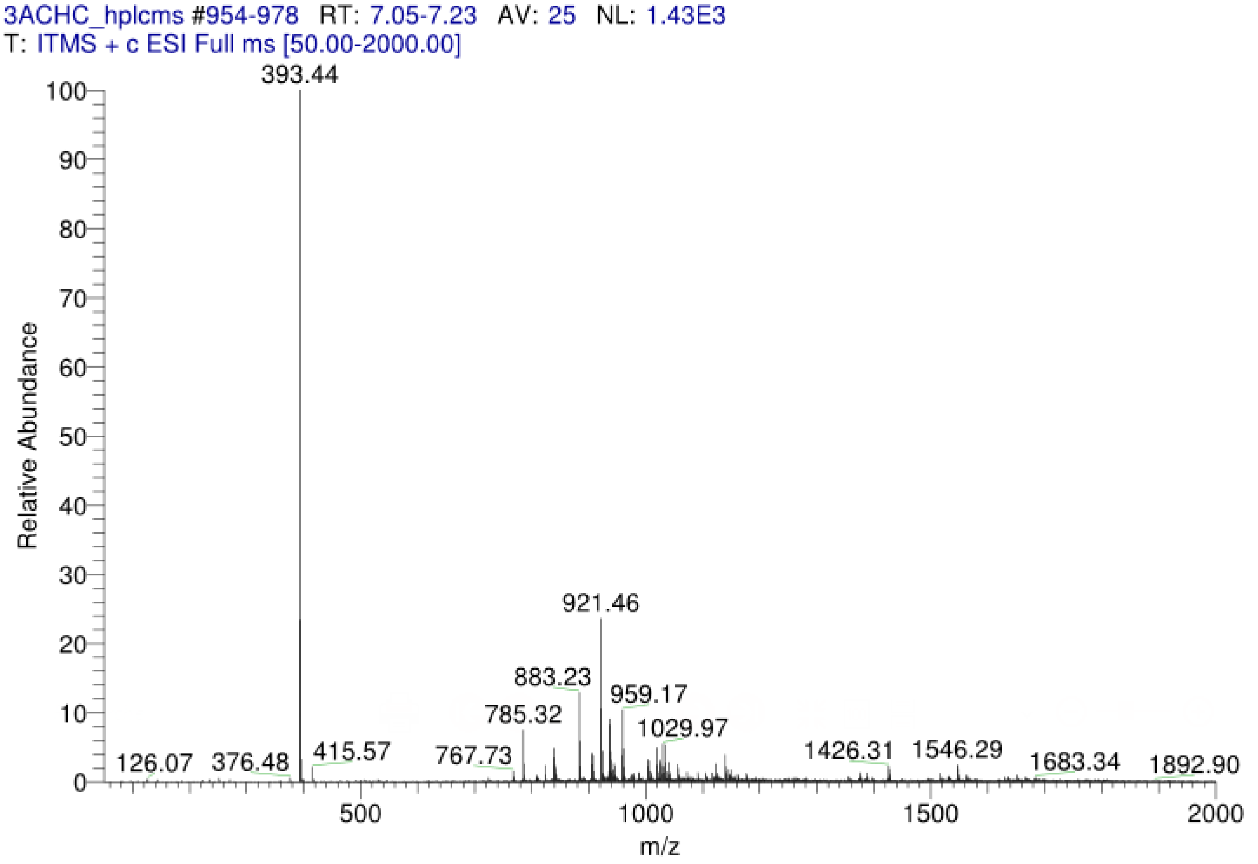

Figure S14. HPLC-MS chromatogram and mass spectrum of **6**.

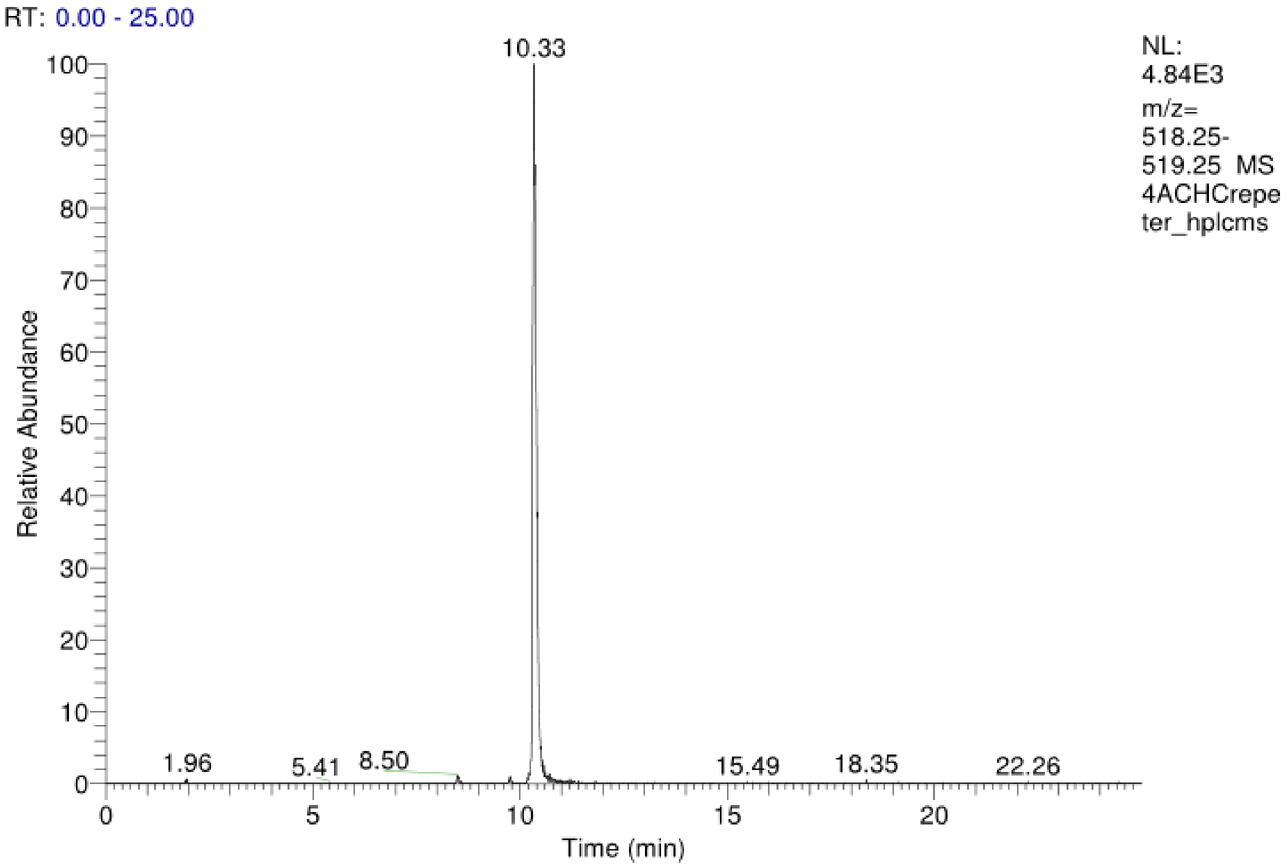

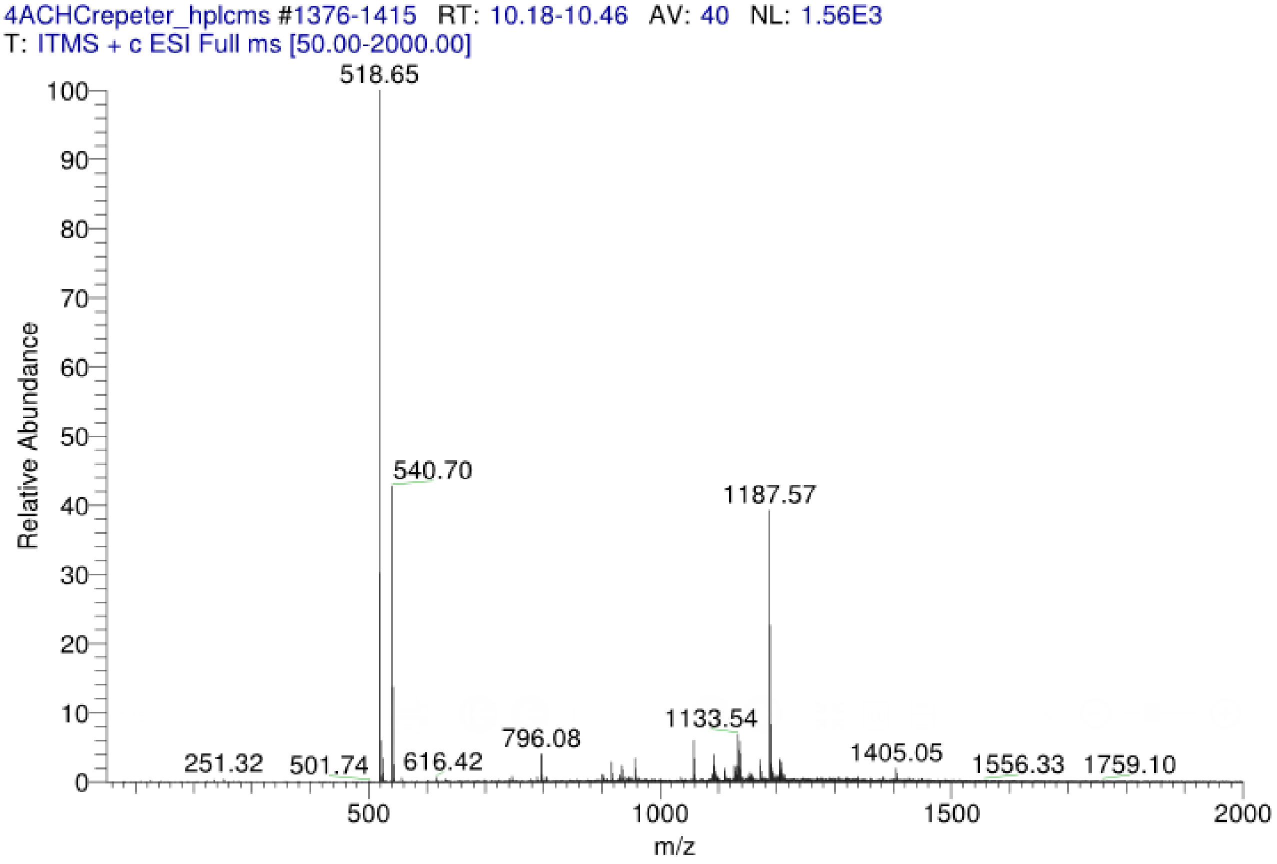

Figure S15. HPLC-MS chromatogram and mass spectrum of **7**.

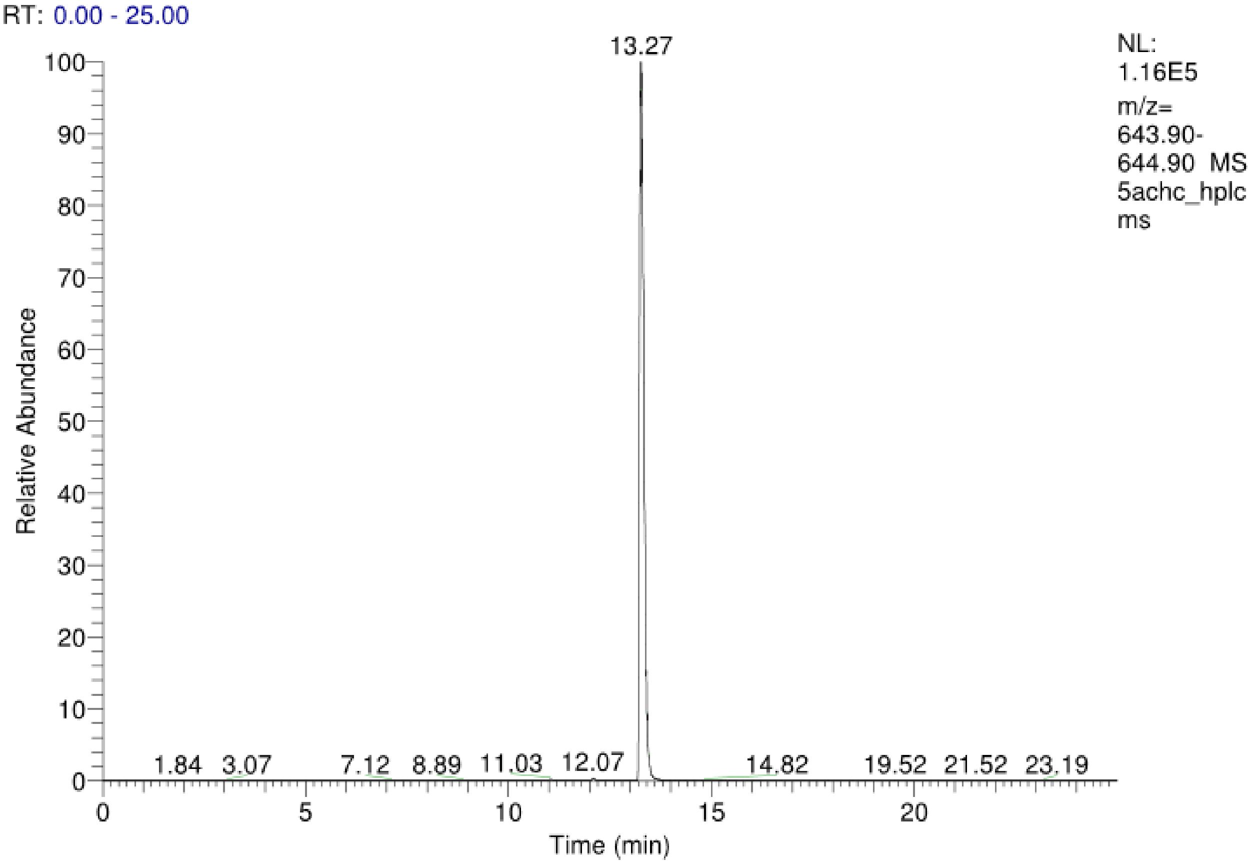

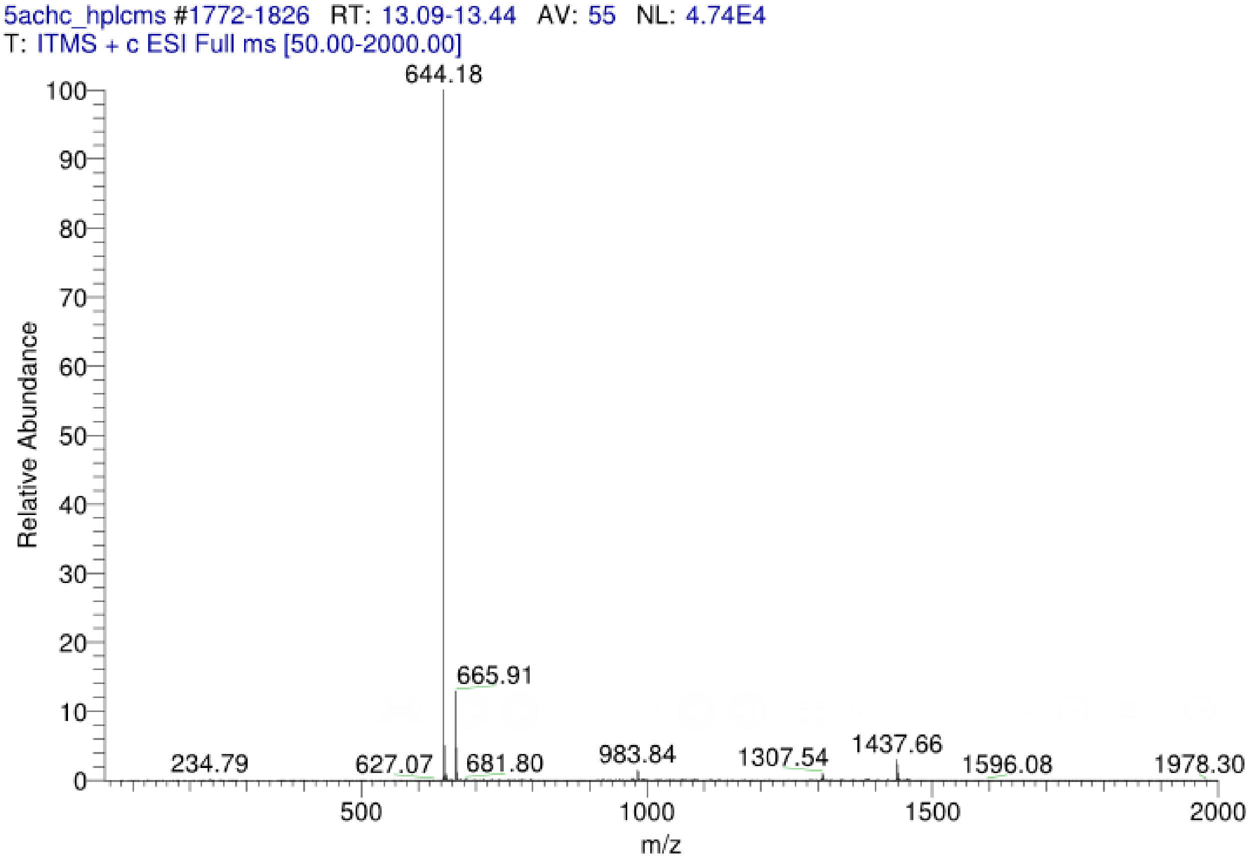

Figure S16. HPLC-MS chromatogram and mass spectrum of **8**.

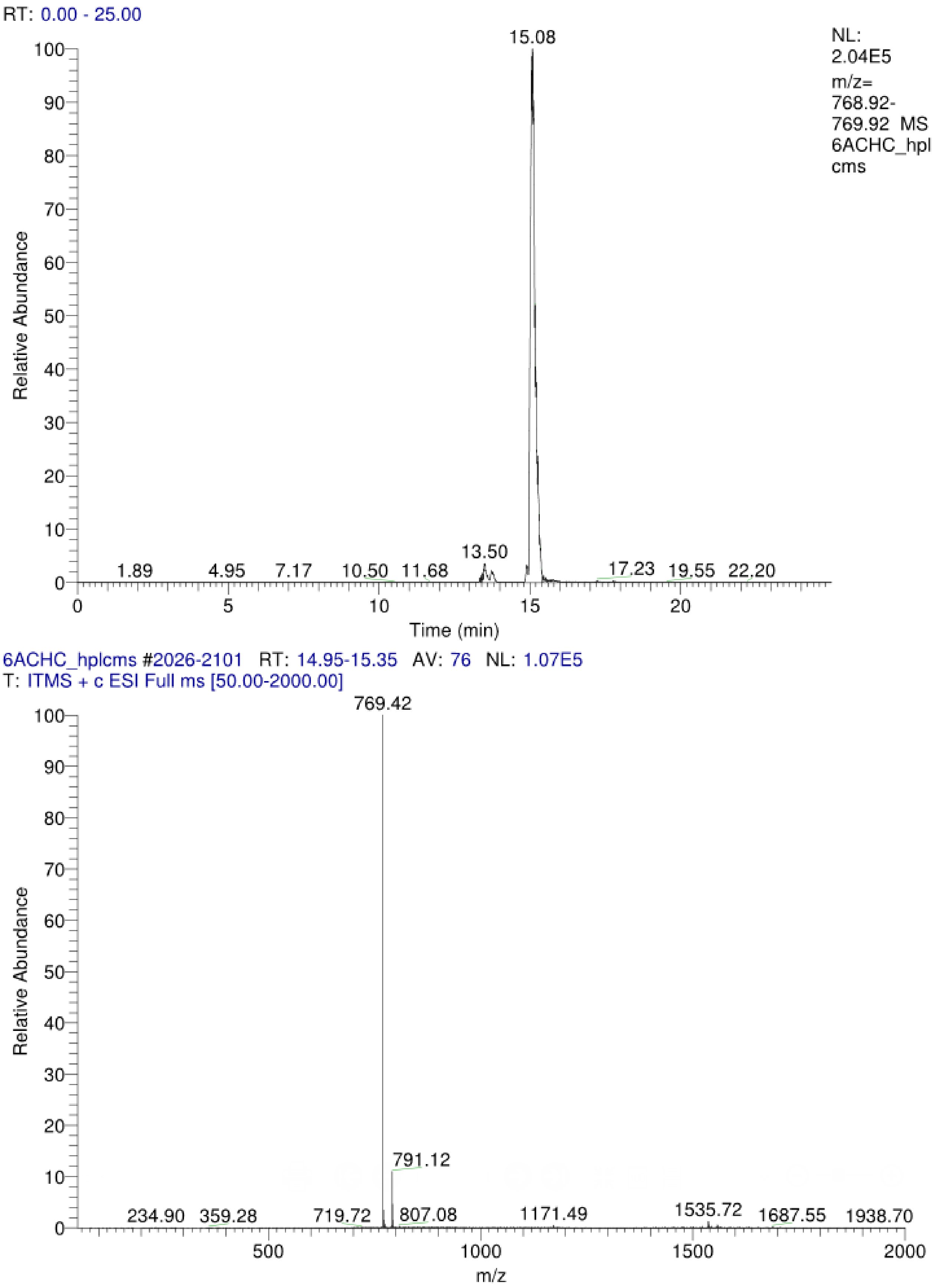

Figure S17. HPLC-MS chromatogram and mass spectrum for the diastereoselective chain elongation of **5** in the absence of water.

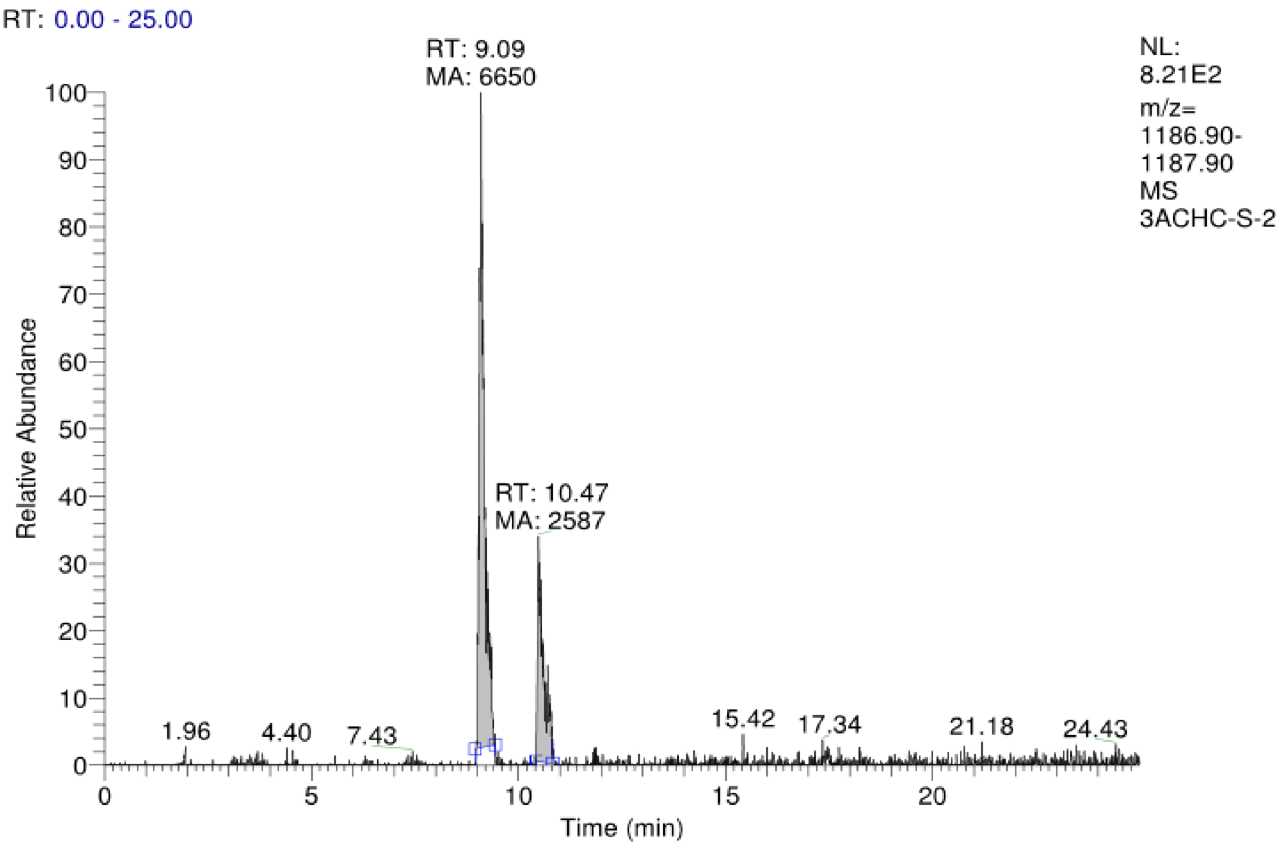

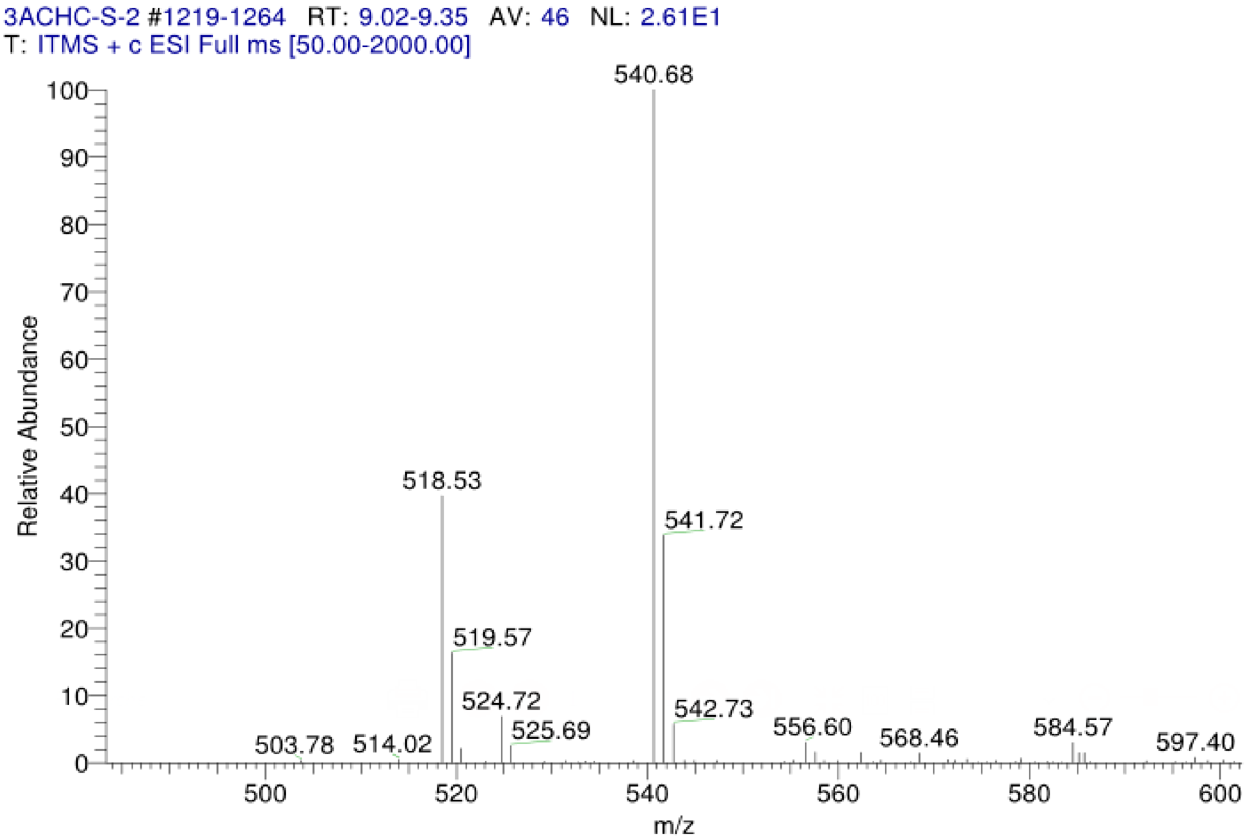

Figure S18. HPLC-MS chromatogram and mass spectrum for the diastereoselective chain elongation of **5** in the presence of water.

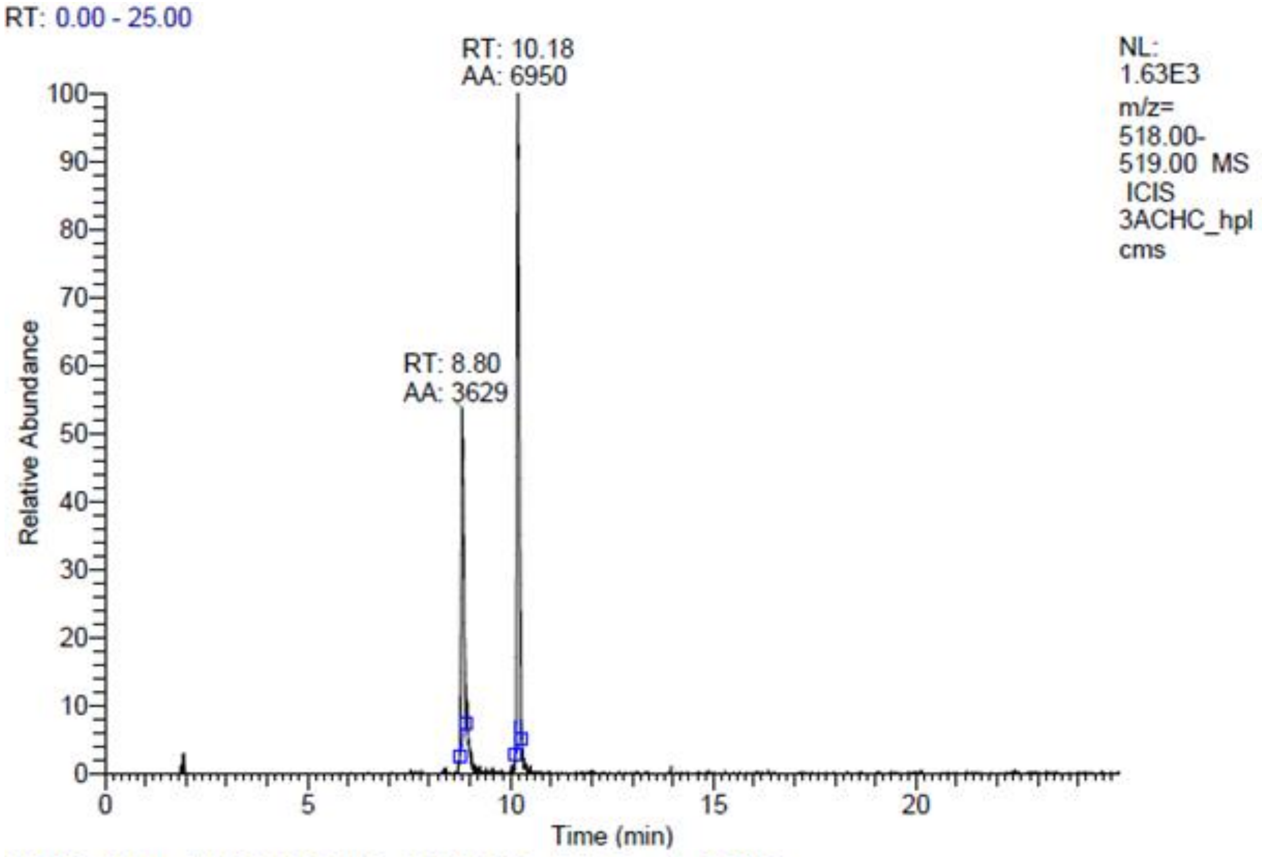

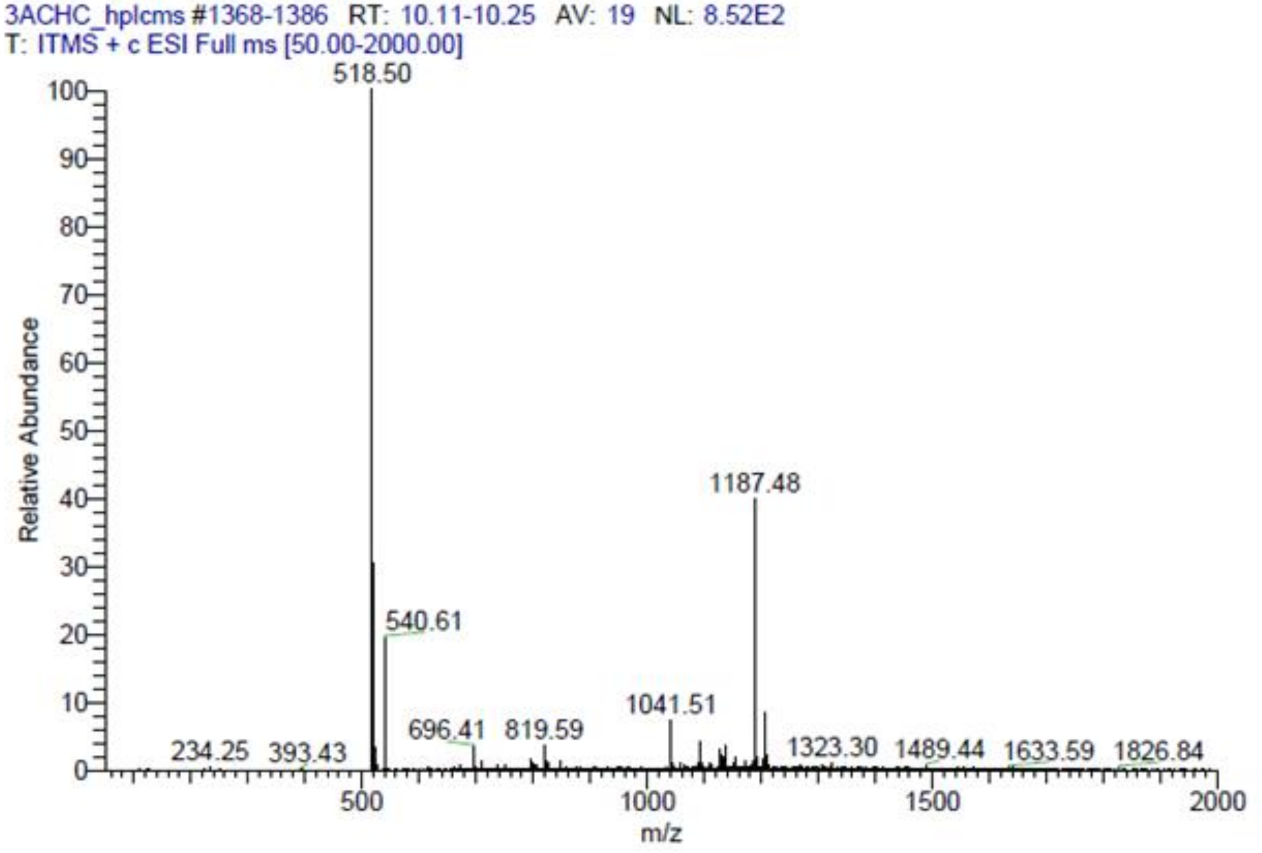

Figure S19. HPLC-MS chromatogram and mass spectrum for the diastereoselective chain elongation of **6** in the absence of water.

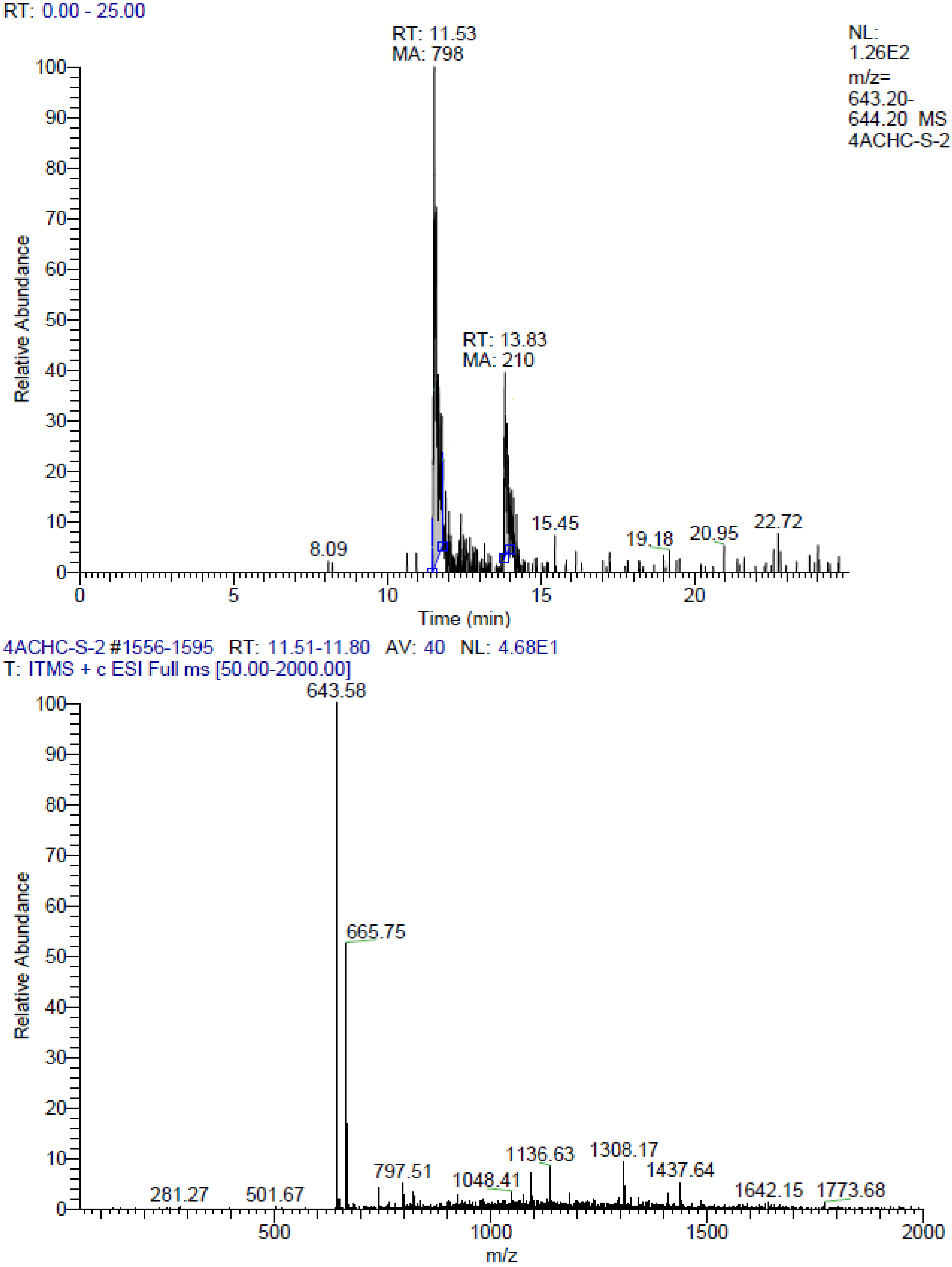

Figure S20. HPLC-MS chromatogram and mass spectrum for the diastereoselective chain elongation of **6** in the presence of water.

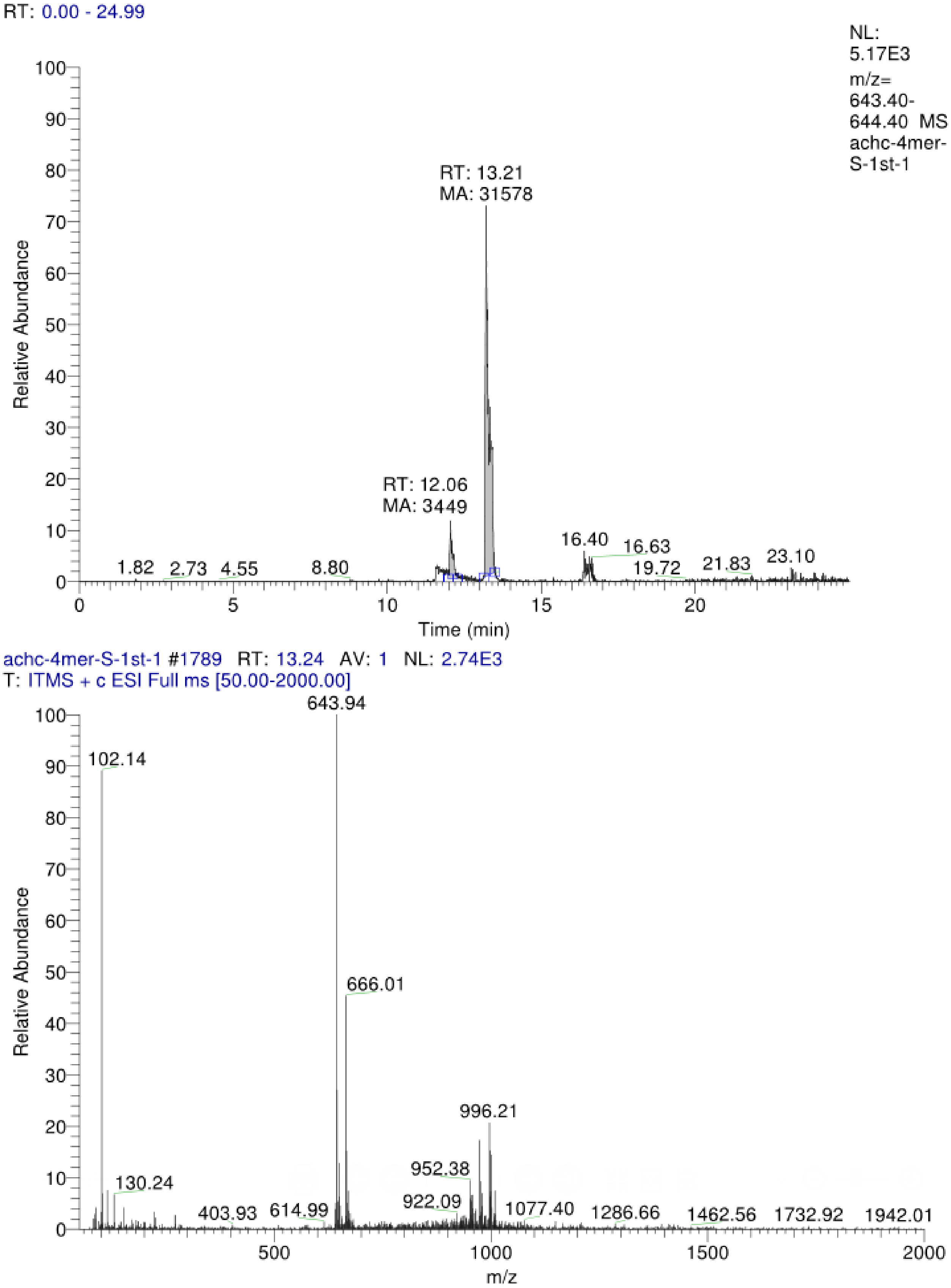

Figure S21. HPLC-MS chromatogram and mass spectrum for the diastereoselective chain elongation of **7** in the absence of water.

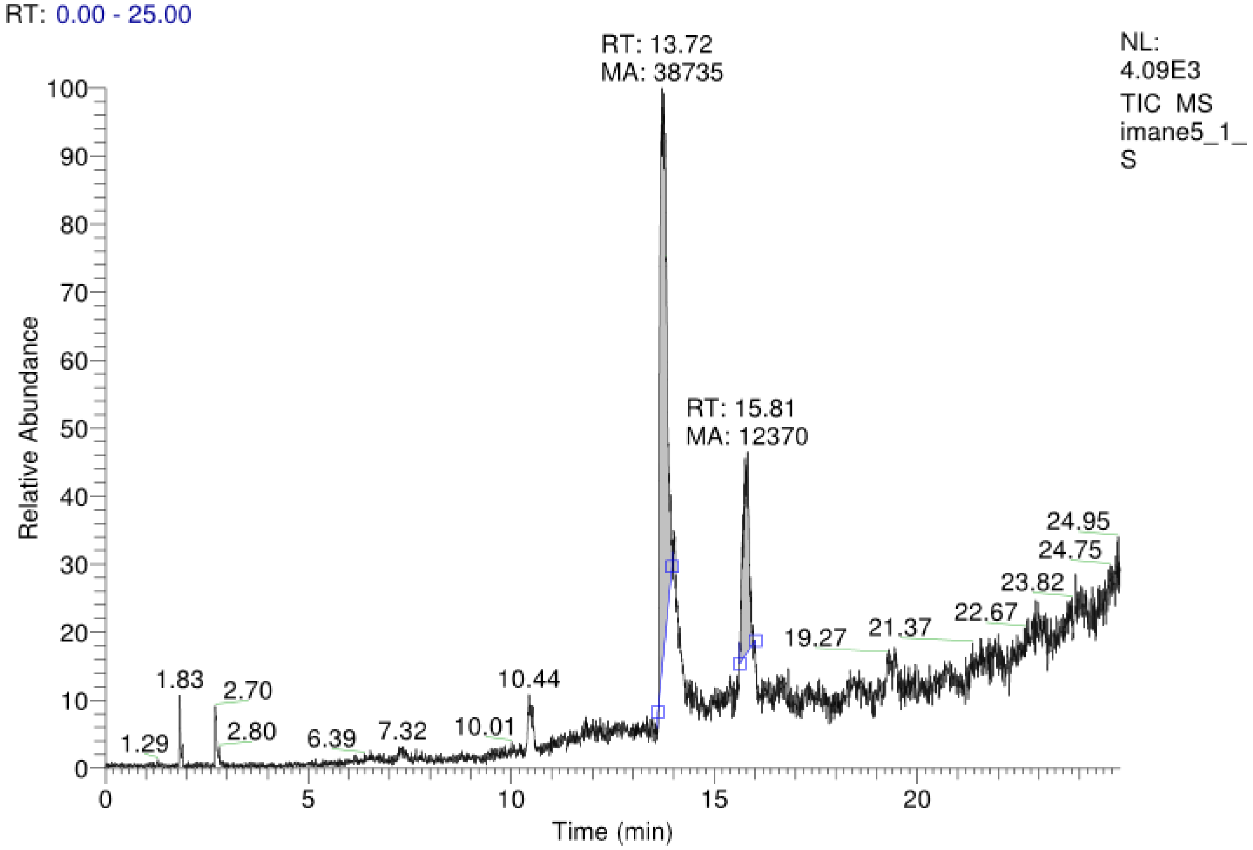

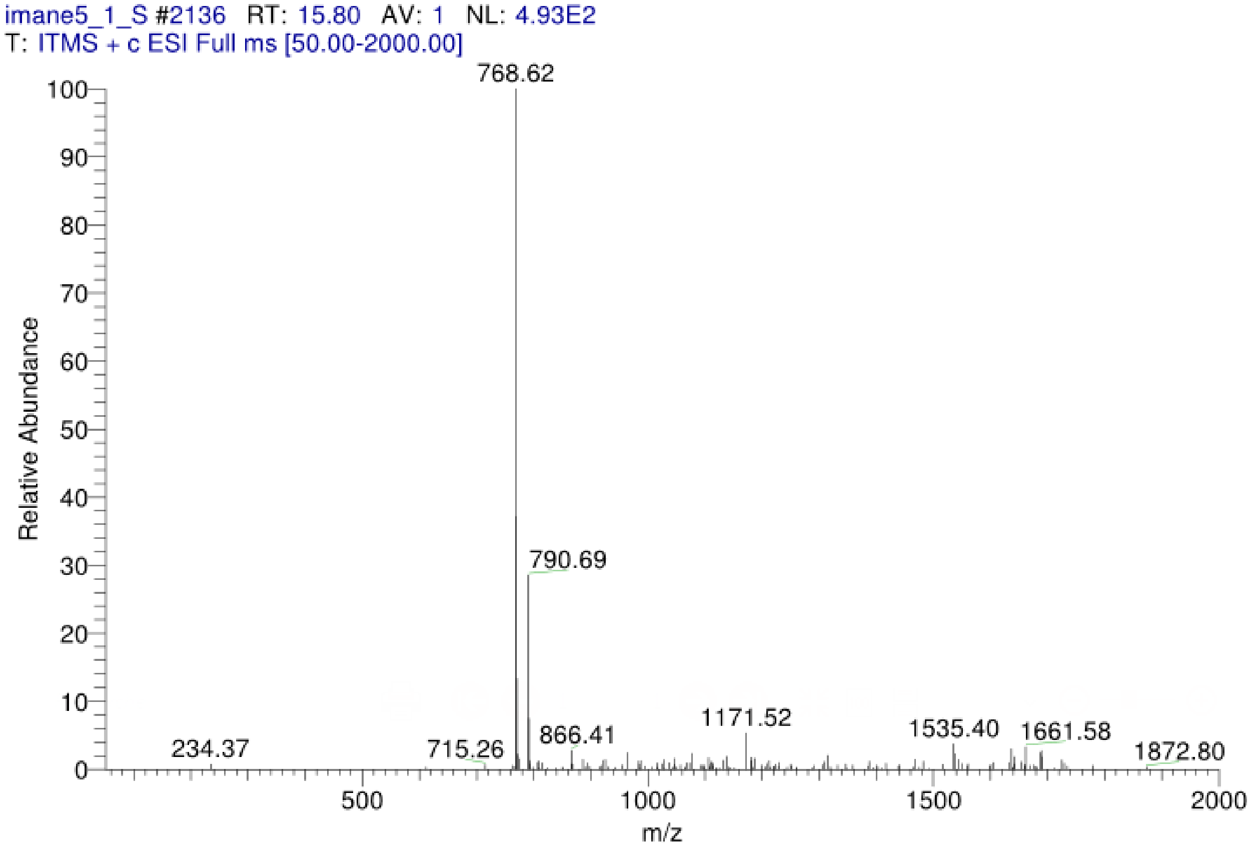

Figure S22. HPLC-MS chromatogram and mass spectrum for the diastereoselective chain elongation of **7** in the presence of water.

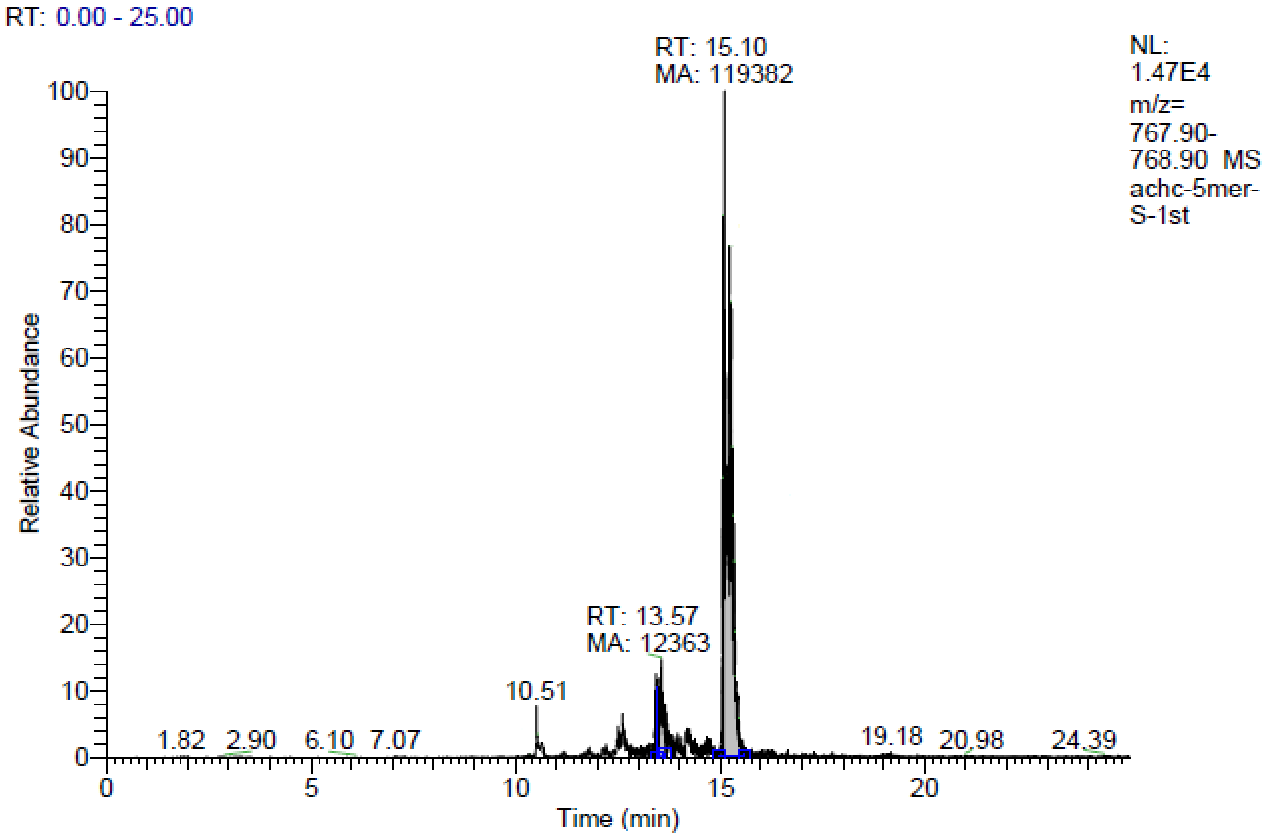

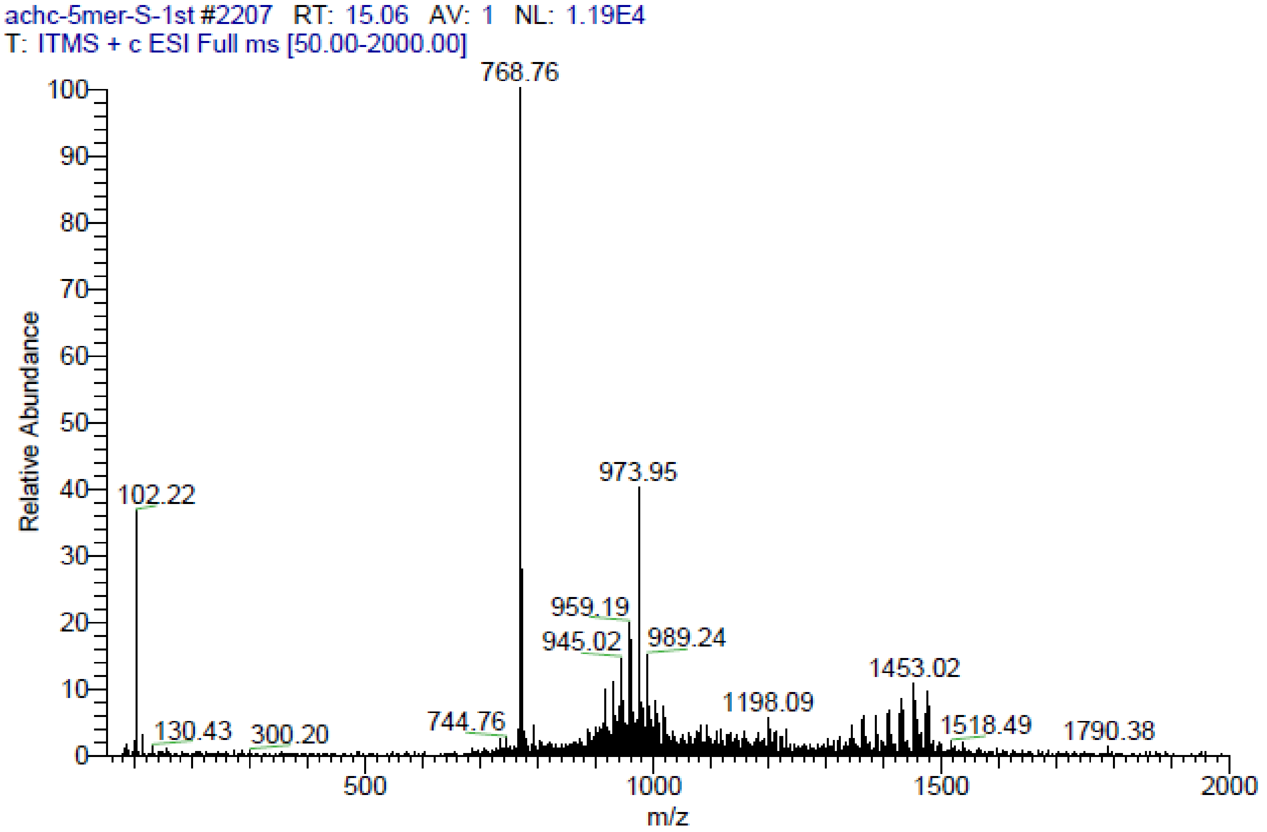

Figure S23. HPLC-MS chromatogram and mass spectrum for the diastereoselective chain elongation of **8** in the absence of water.

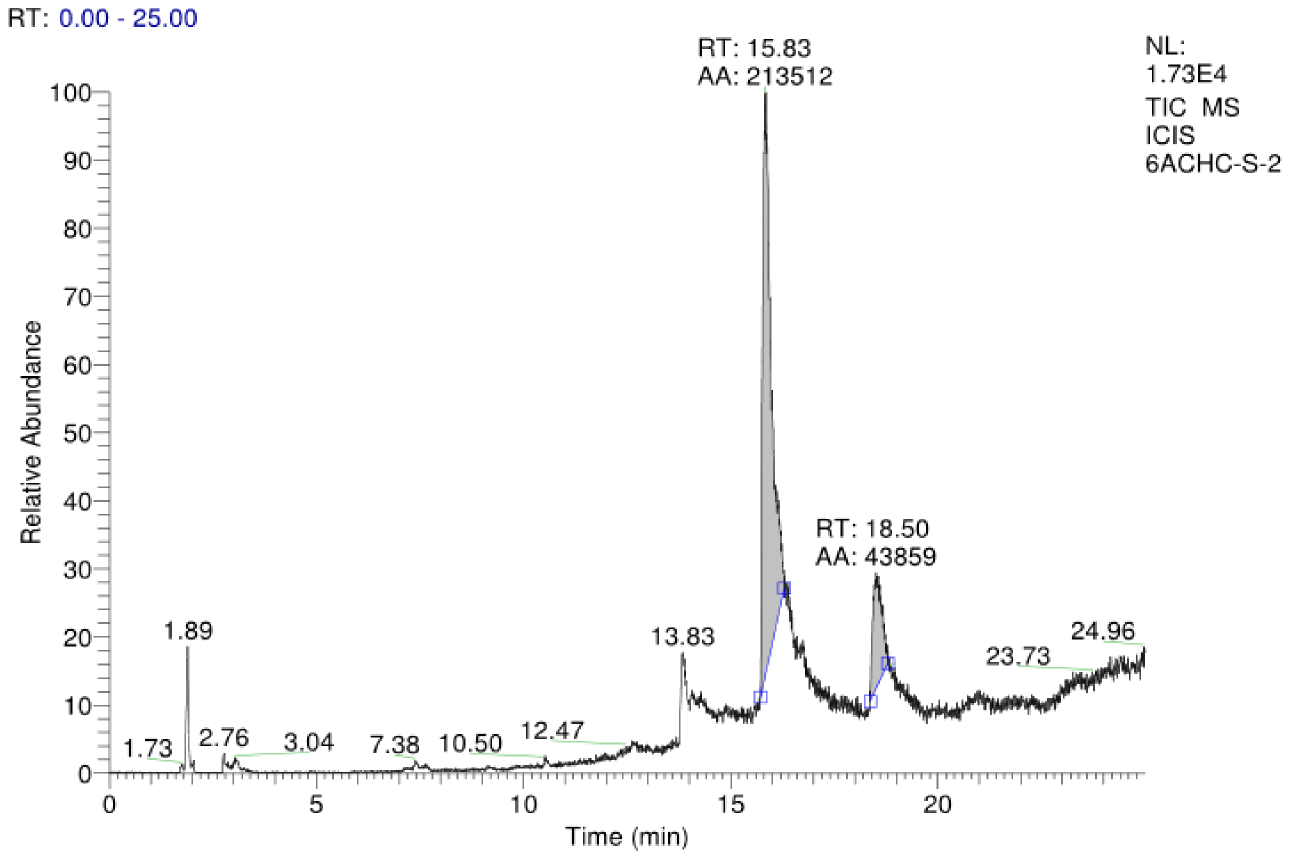

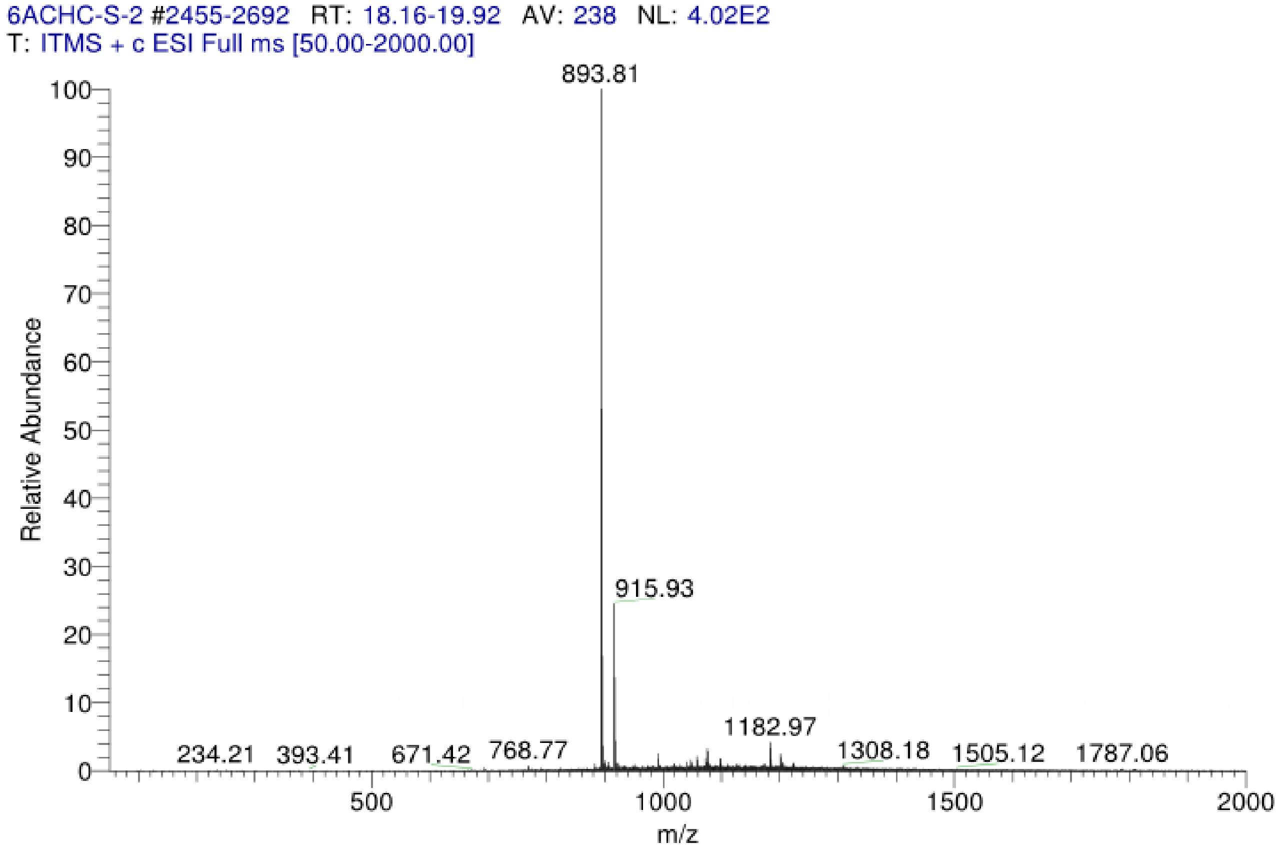

Figure S24. HPLC-MS chromatogram and mass spectrum for the diastereoselective chain elongation of **8** in the presence of water.

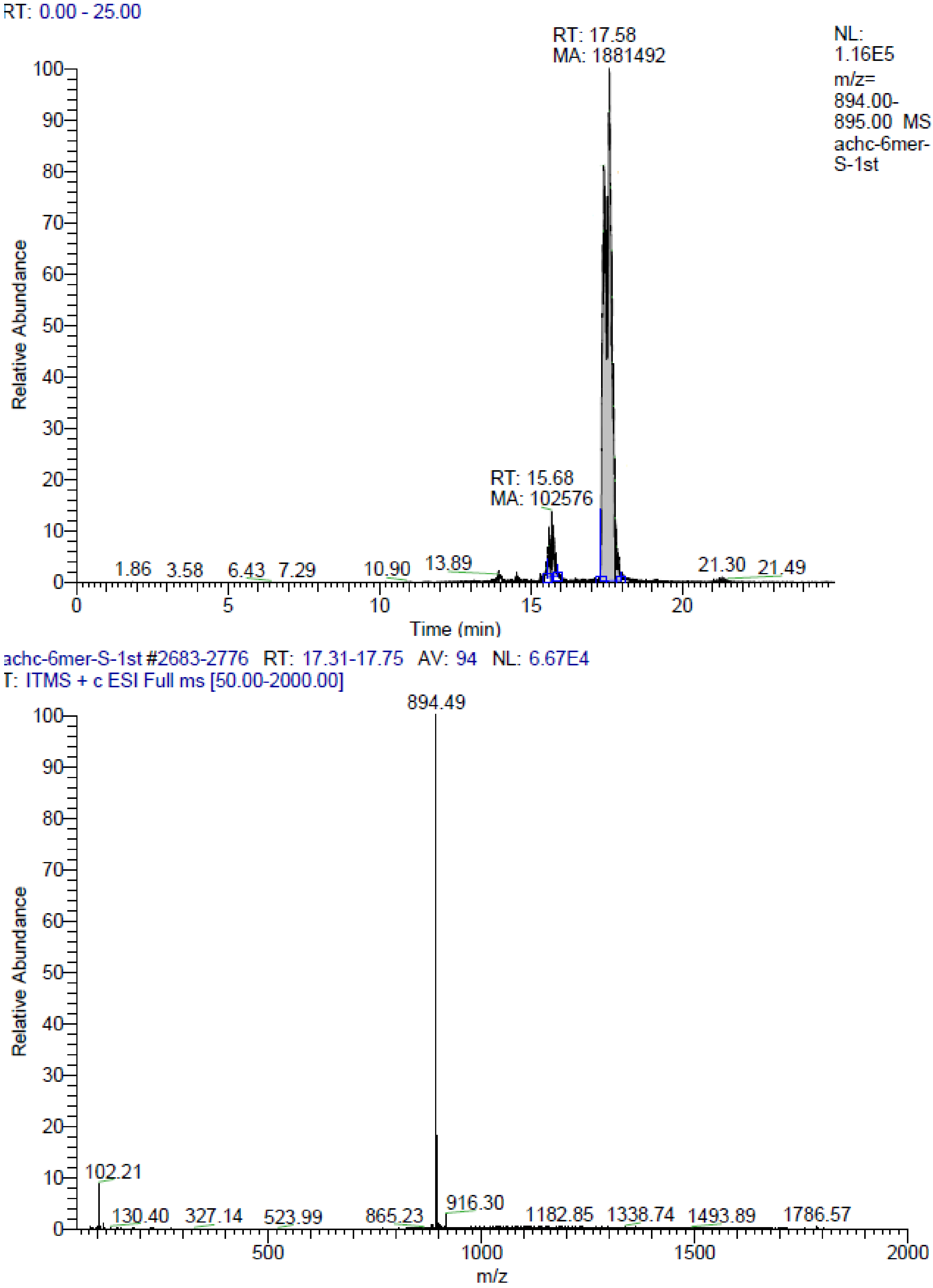

Figure S25. HPLC-MS chromatogram and mass spectrum of **9**.

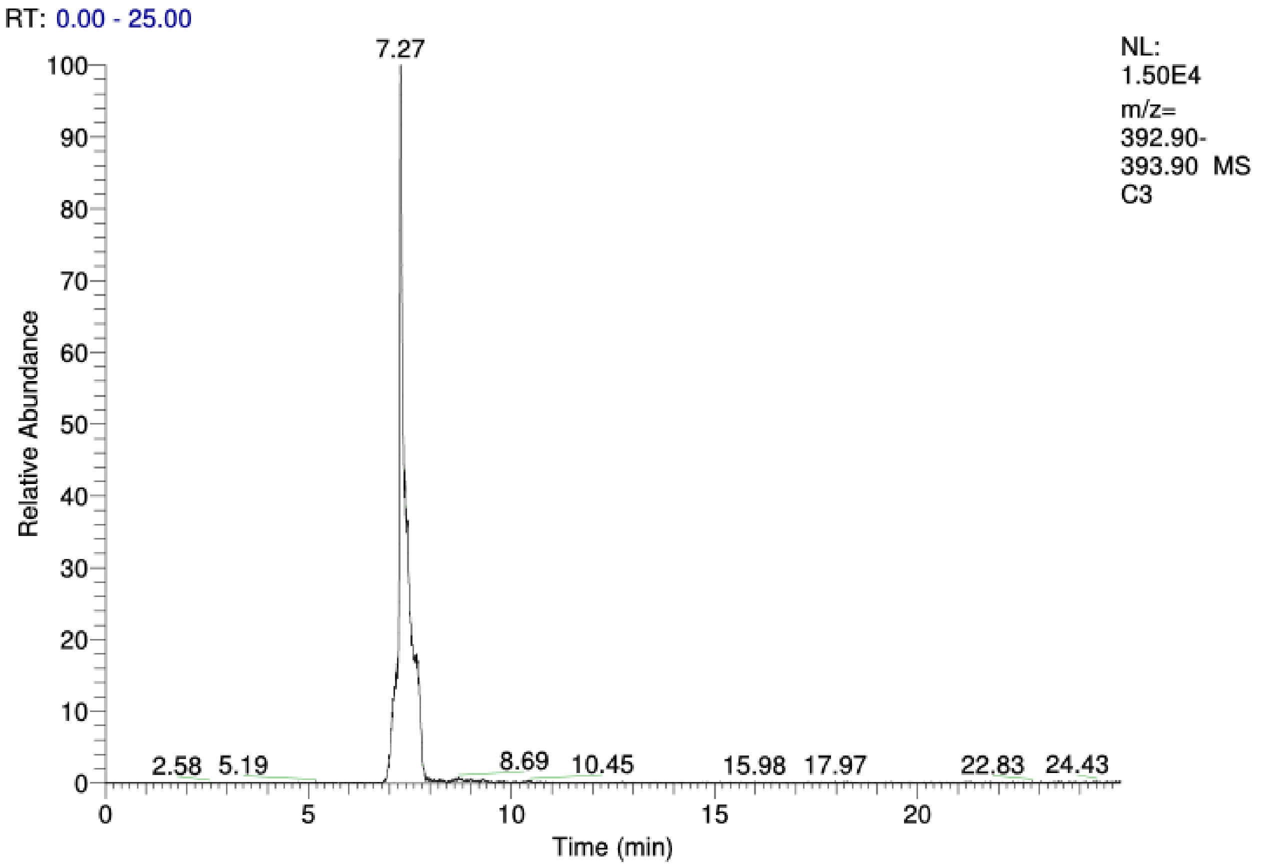

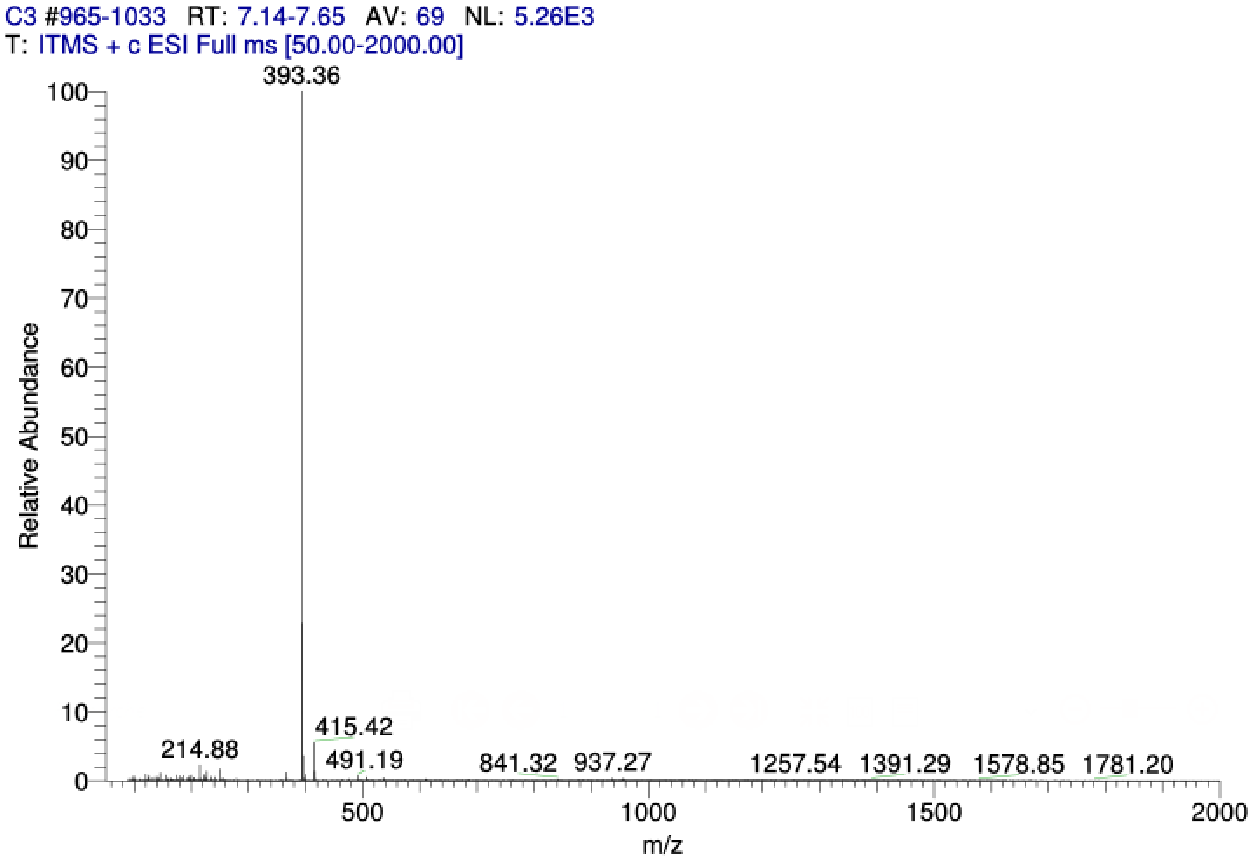

Figure S26. HPLC-MS chromatogram and mass spectrum of **10**.

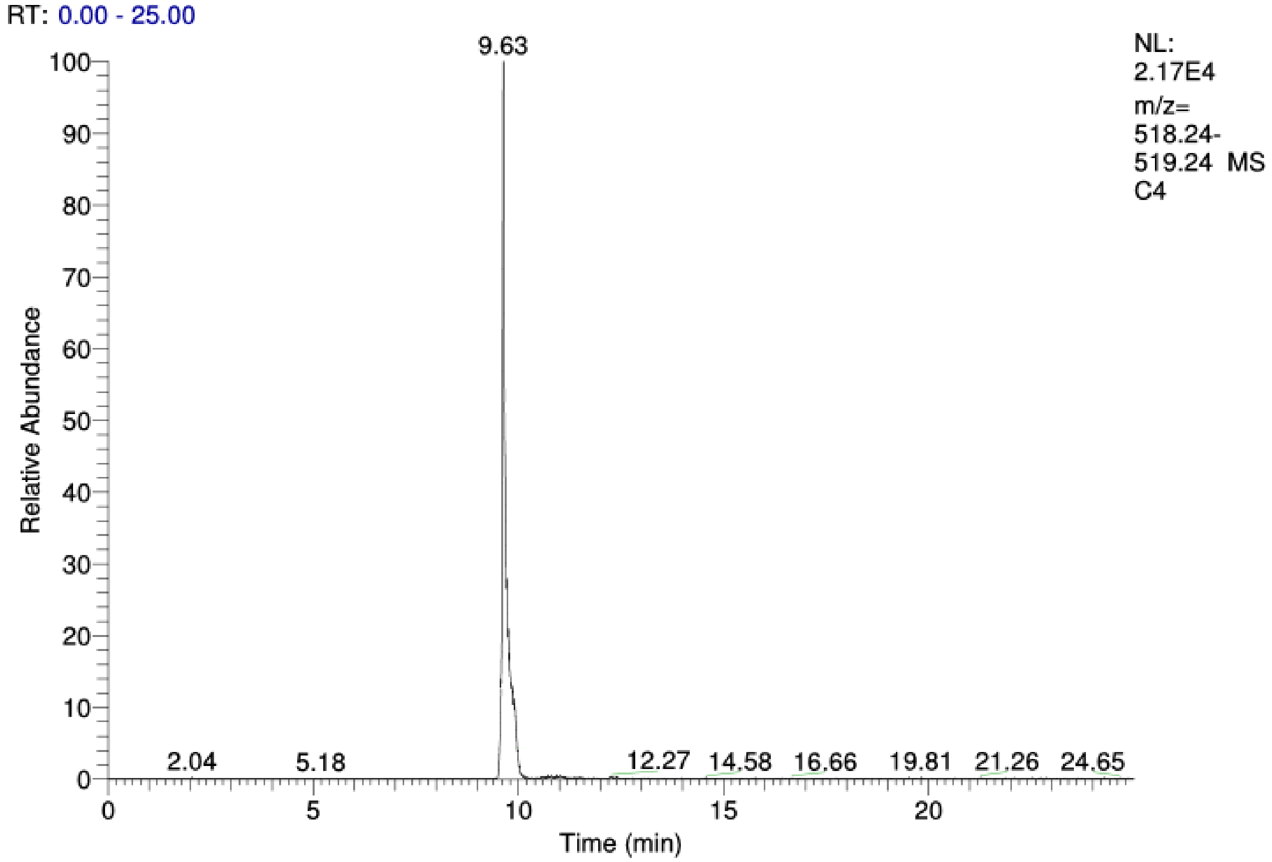

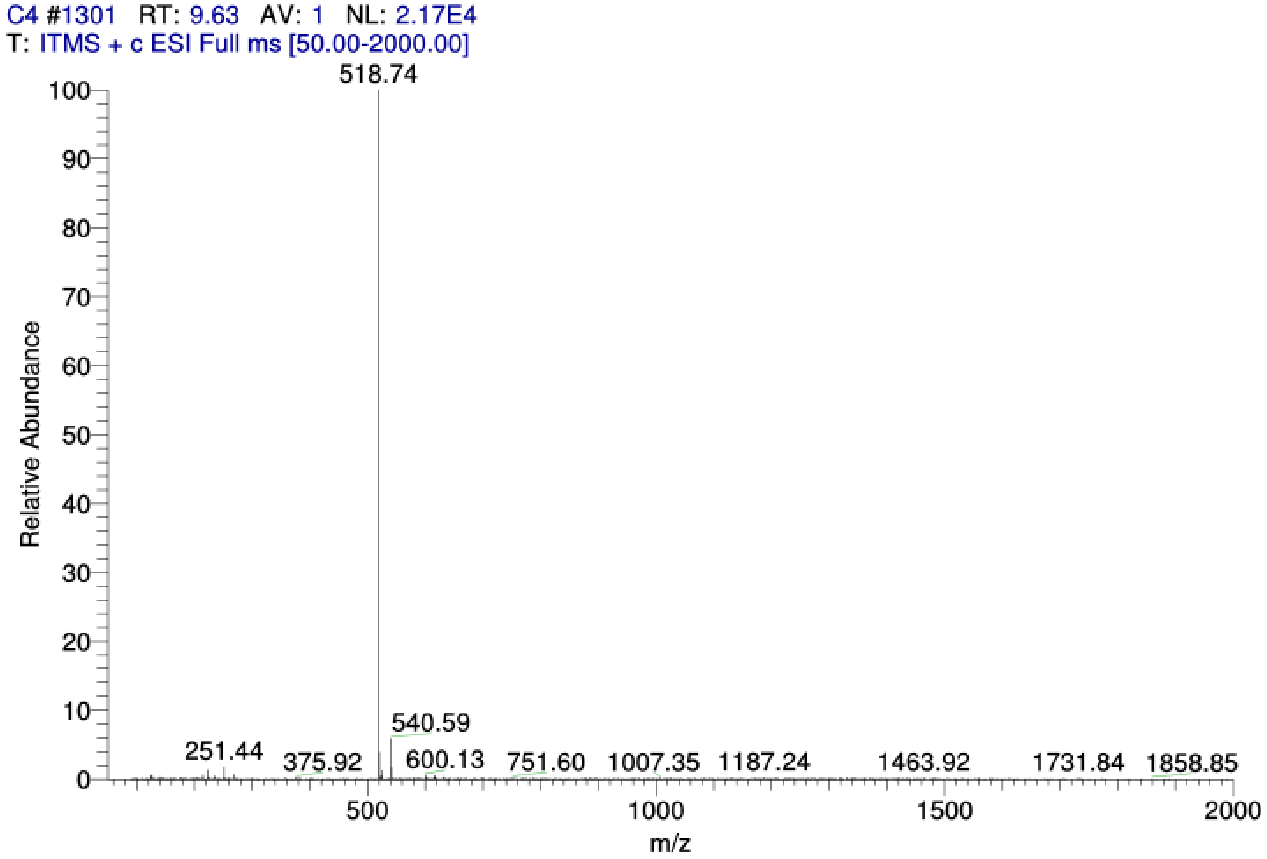

Figure S27. HPLC-MS chromatogram and mass spectrum of **11**.

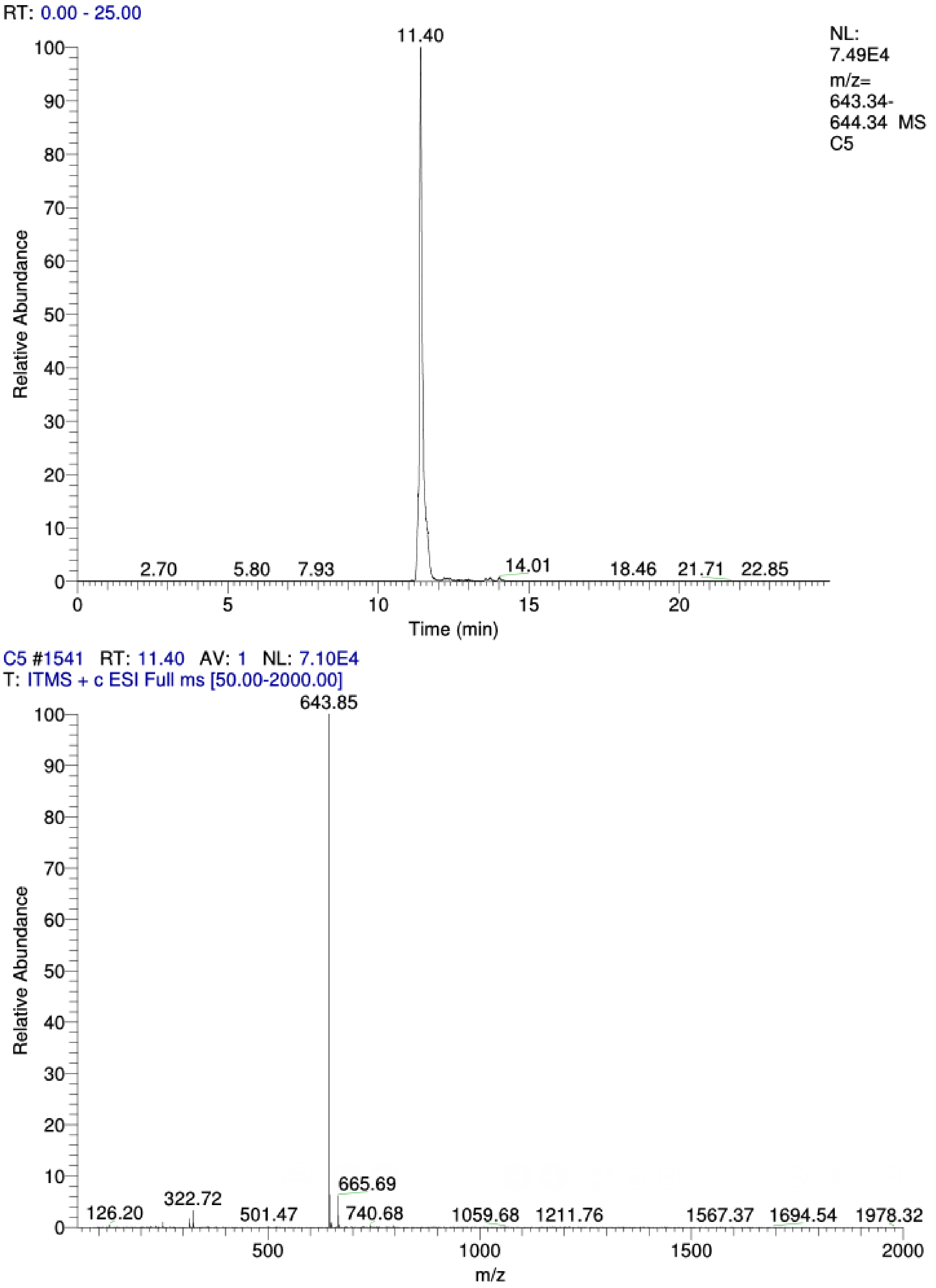

Figure S28. HPLC-MS chromatogram and mass spectrum of **12**.

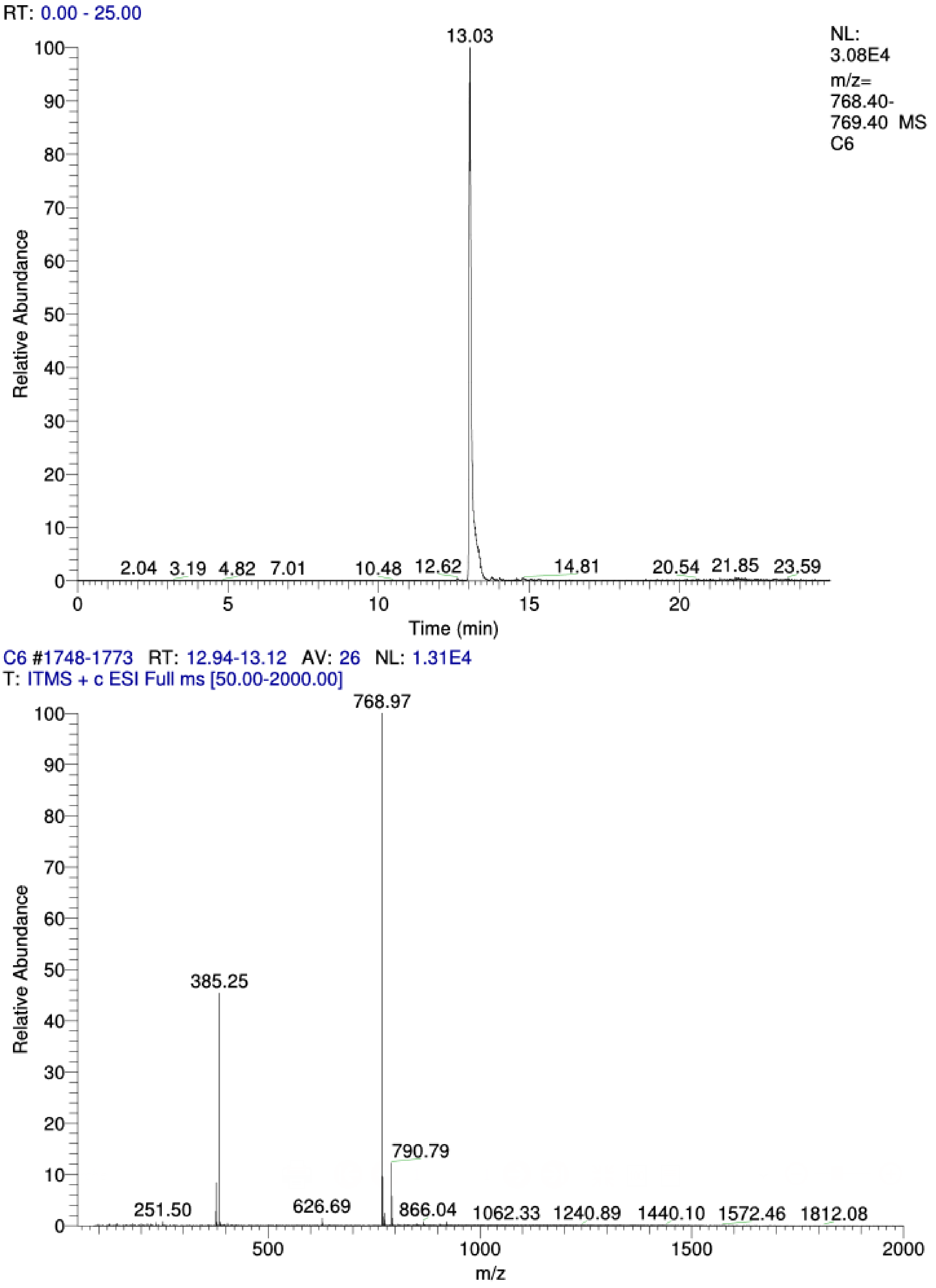

Figure S29. HPLC-MS chromatogram and mass spectrum for the diastereoselective chain elongation of **9** in the absence of water.

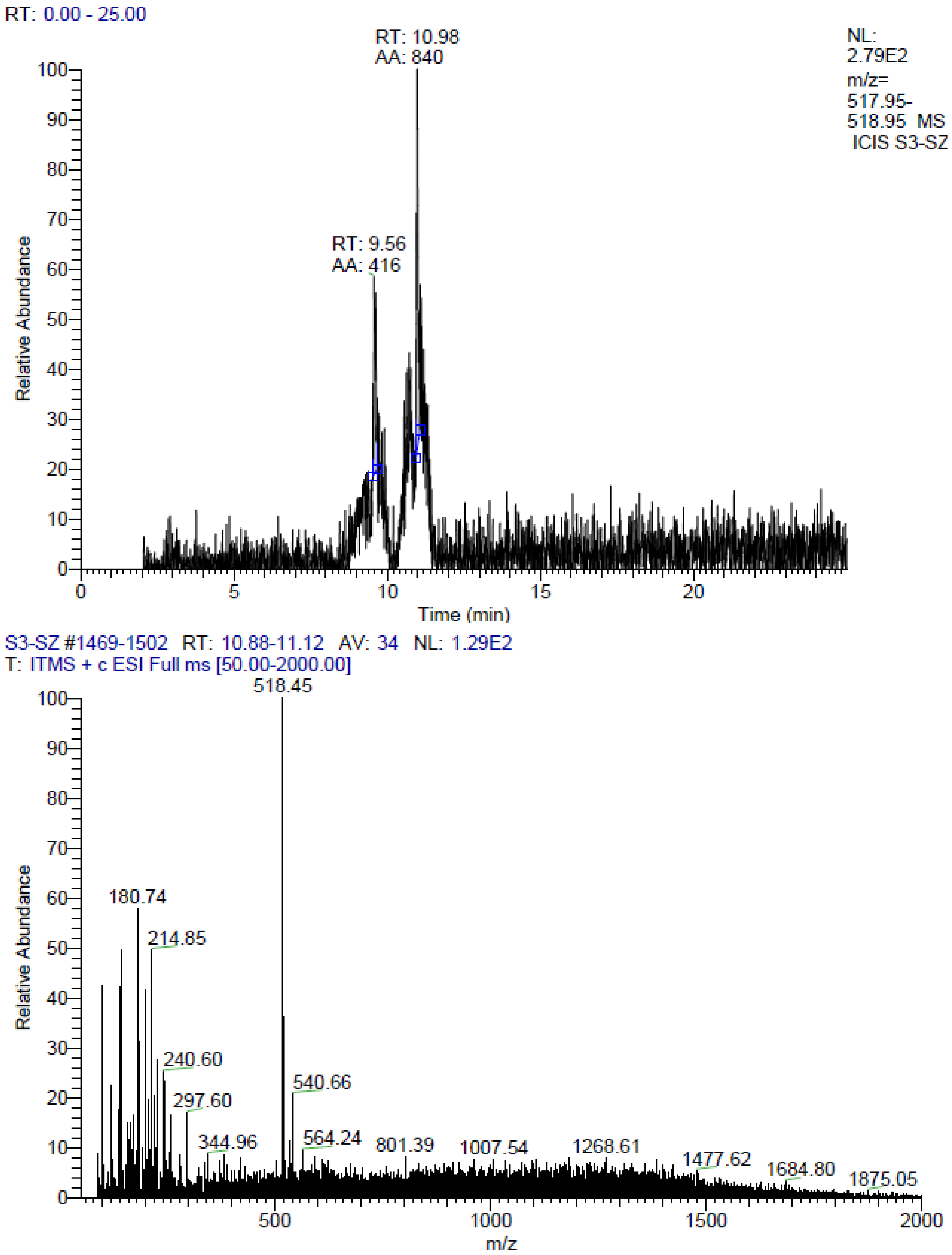

Figure S30. HPLC-MS chromatogram and mass spectrum for the diastereoselective chain elongation of **9** in the presence of water.

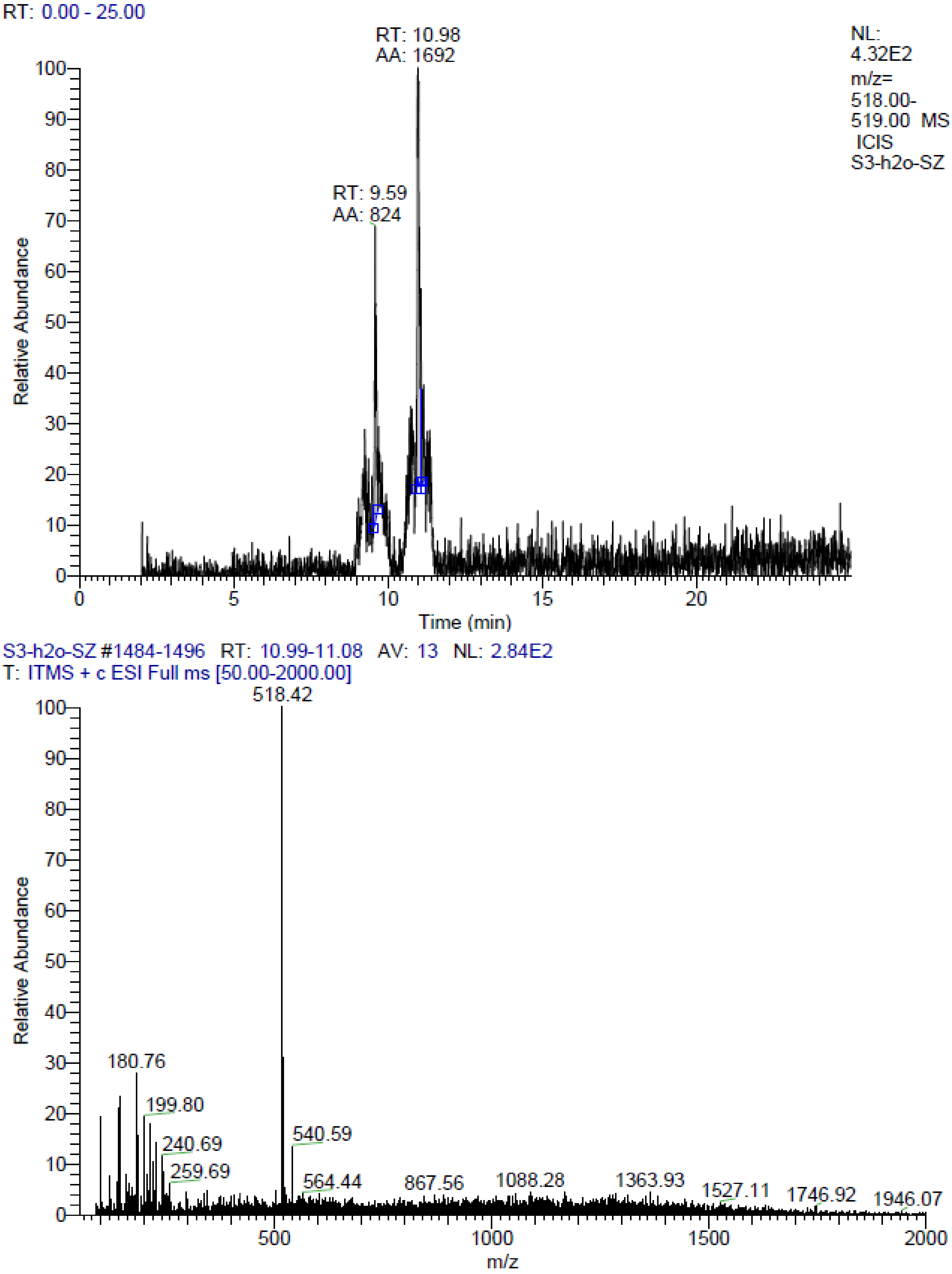

Figure S31. HPLC-MS chromatogram and mass spectrum for the diastereoselective chain elongation of **10** in the absence of water.

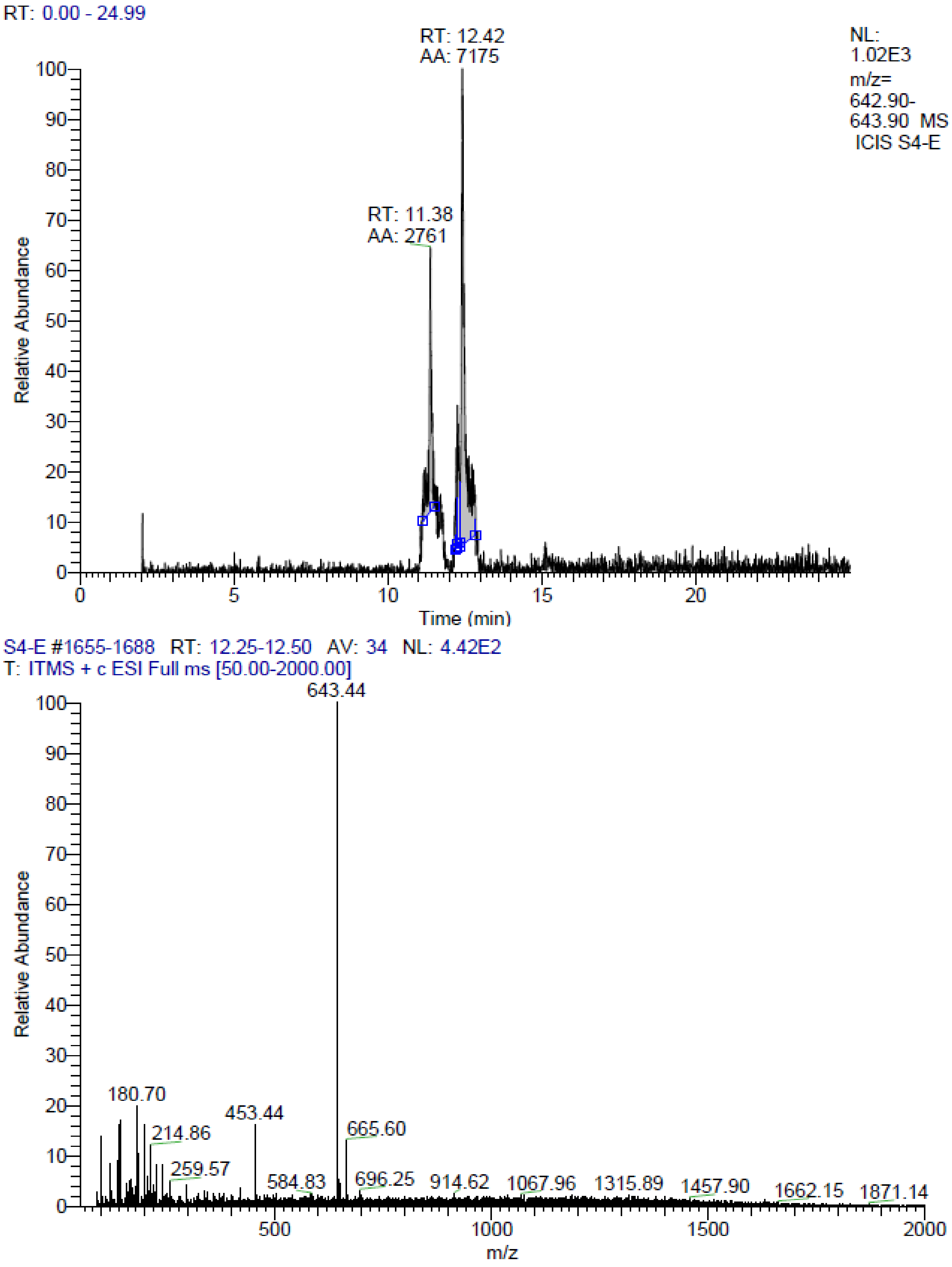

Figure S32. HPLC-MS chromatogram and mass spectrum for the diastereoselective chain elongation of **10** in the presence of water.

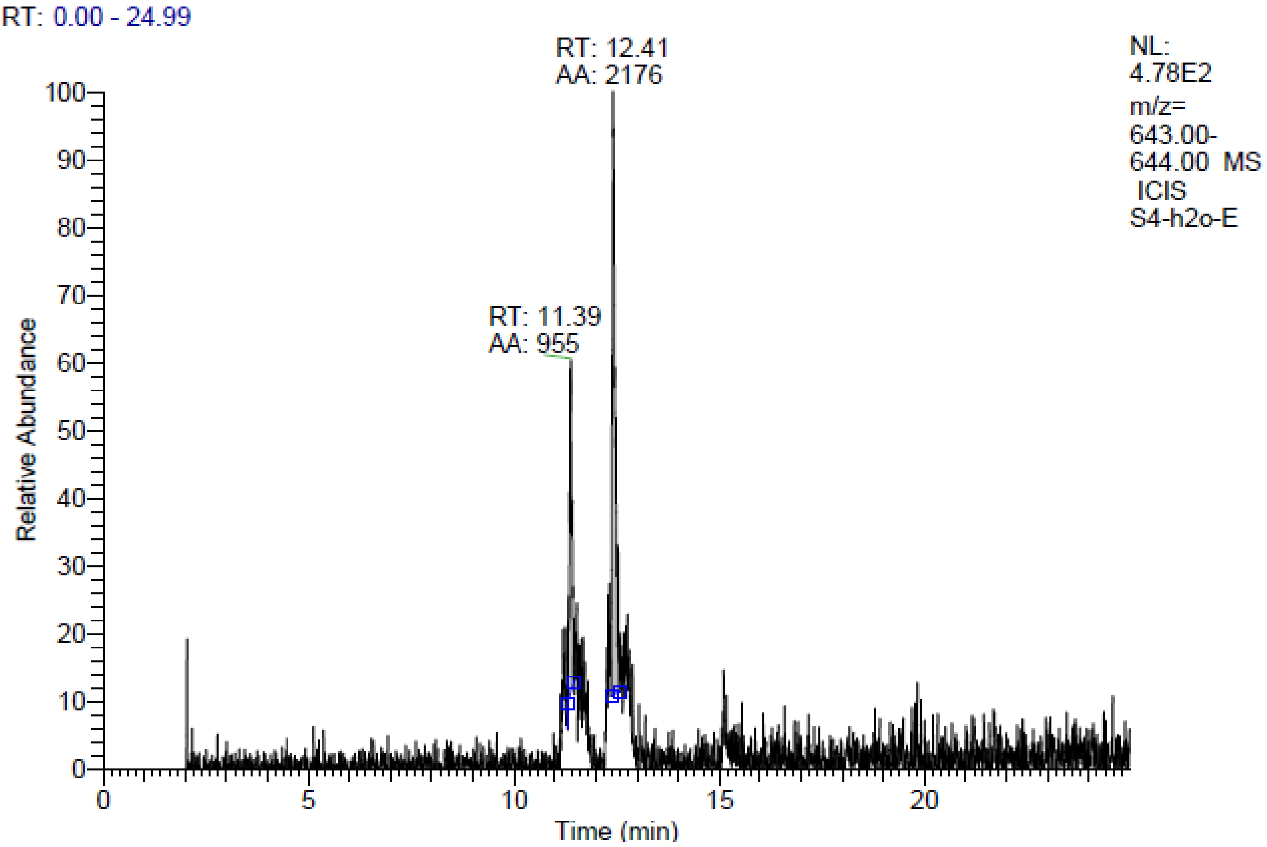

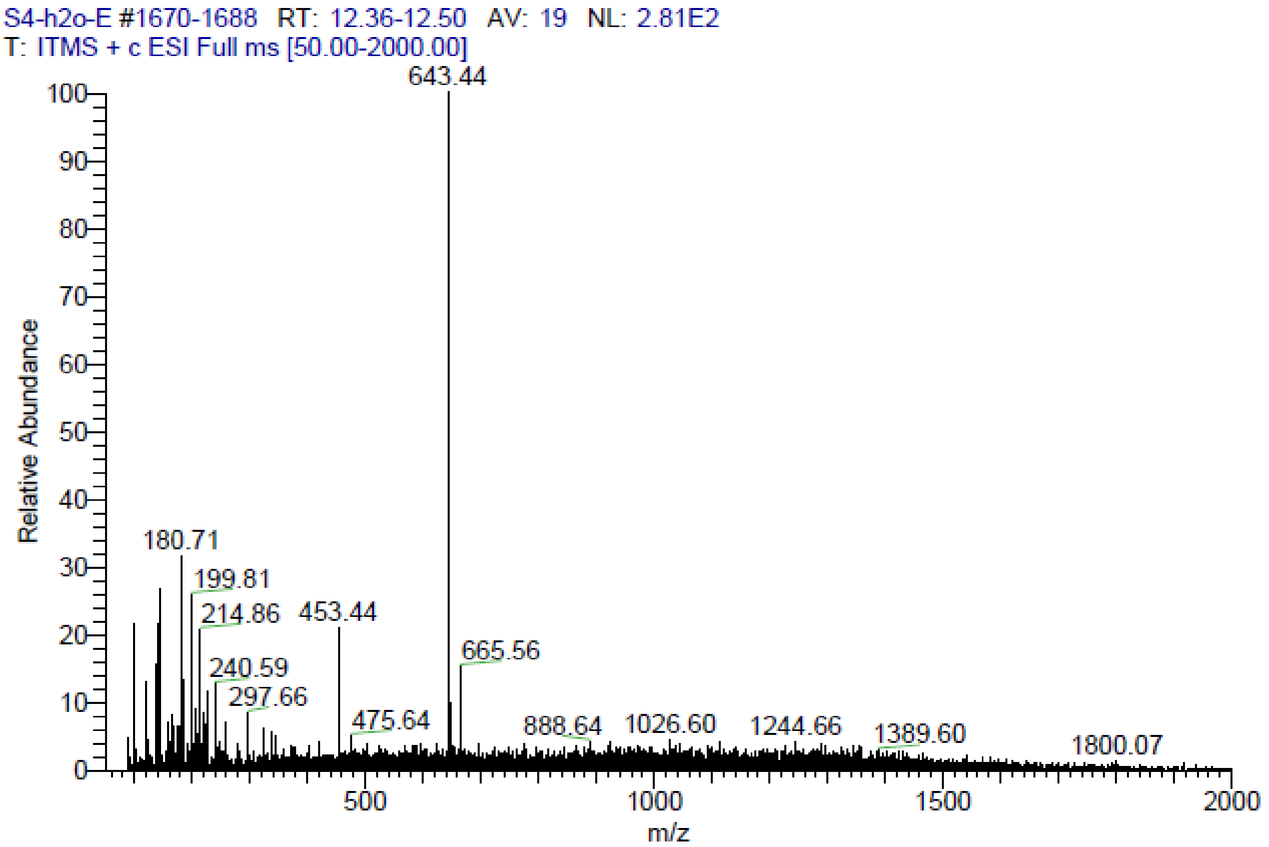

Figure S33. HPLC-MS chromatogram and mass spectrum for the diastereoselective chain elongation of **11** in the absence of water.

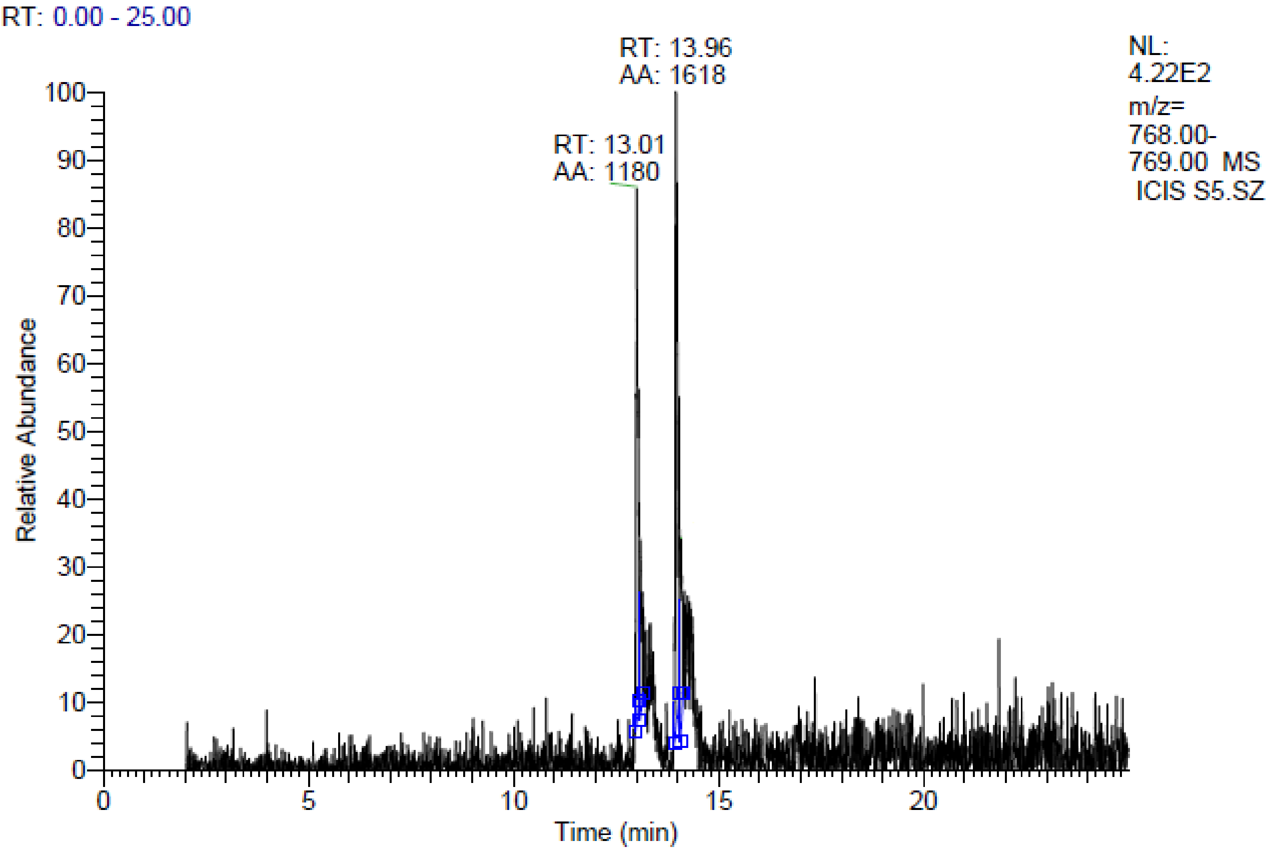

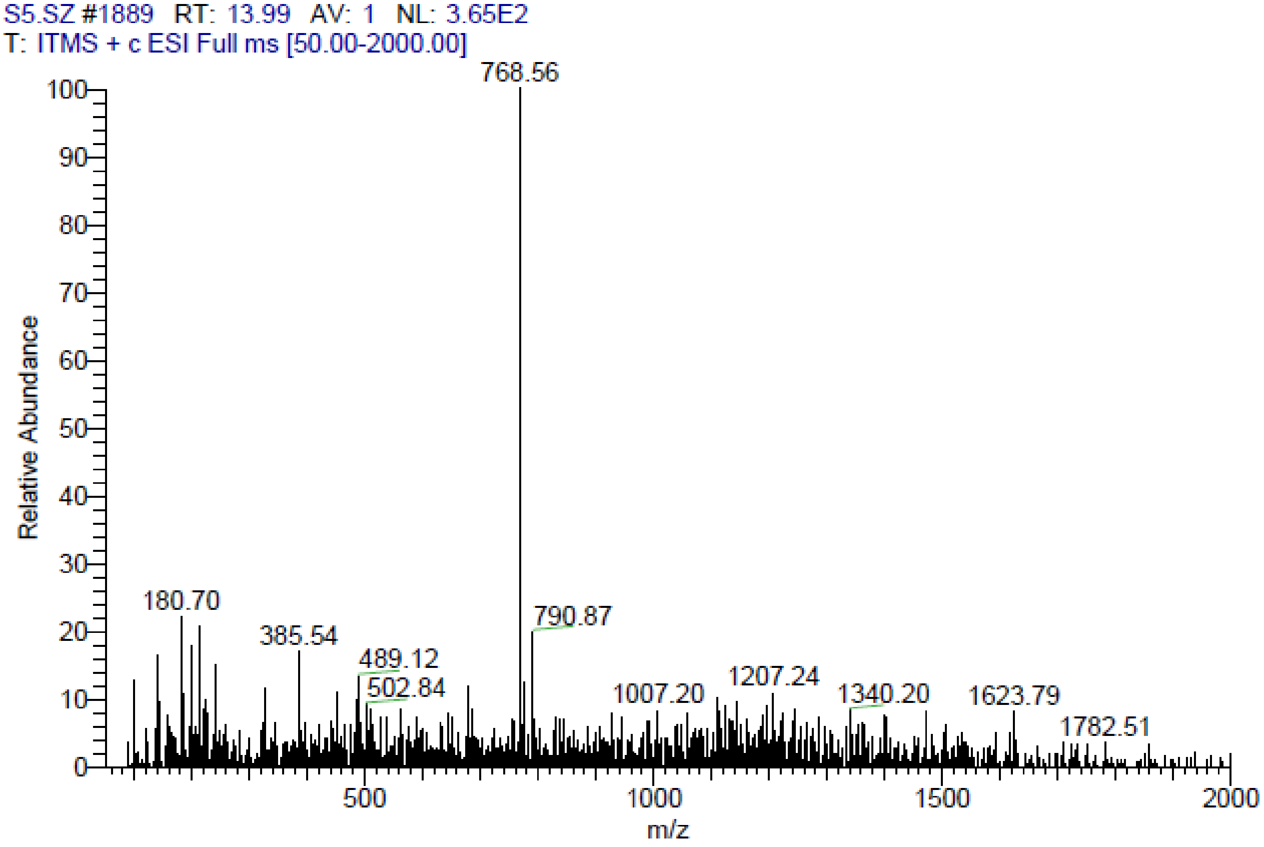

Figure S34. HPLC-MS chromatogram and mass spectrum for the diastereoselective chain elongation of **11** in the presence of water.

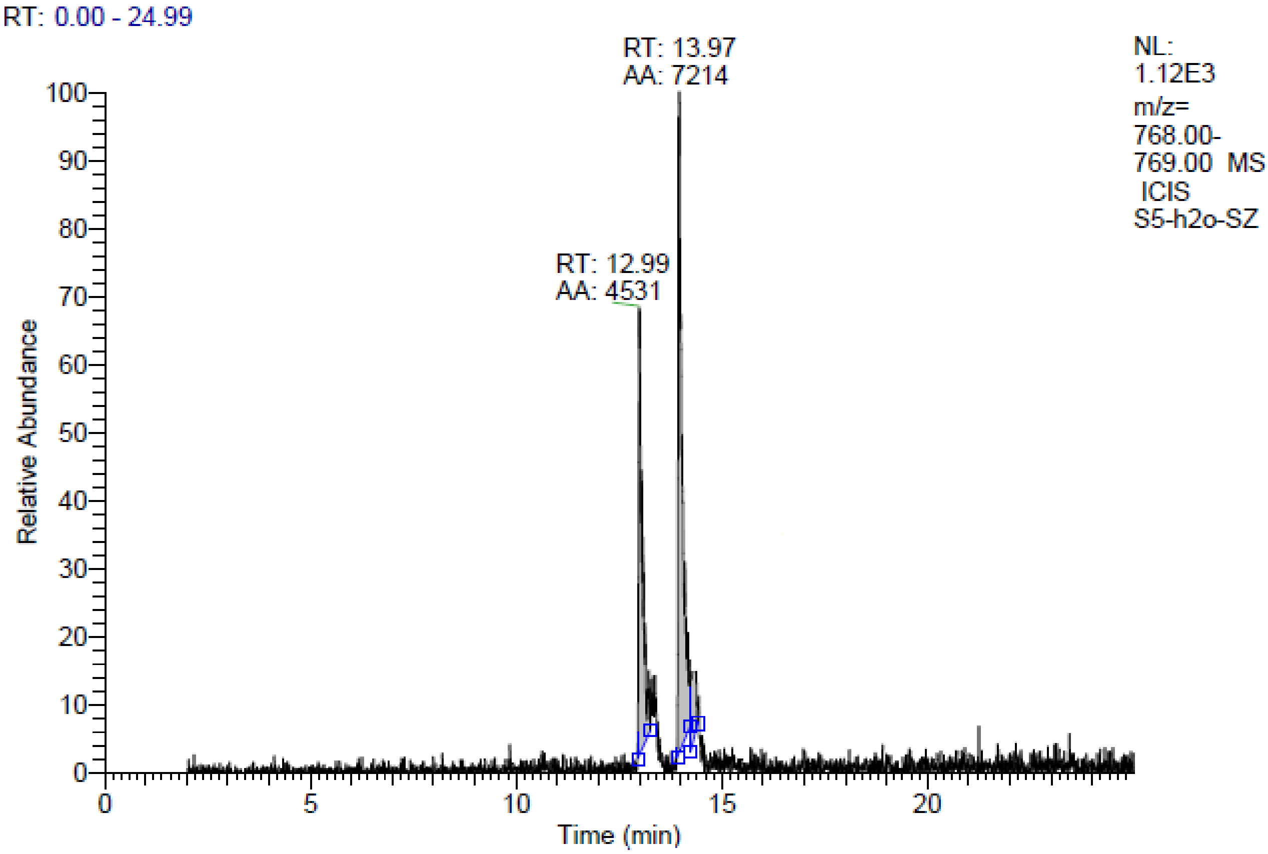

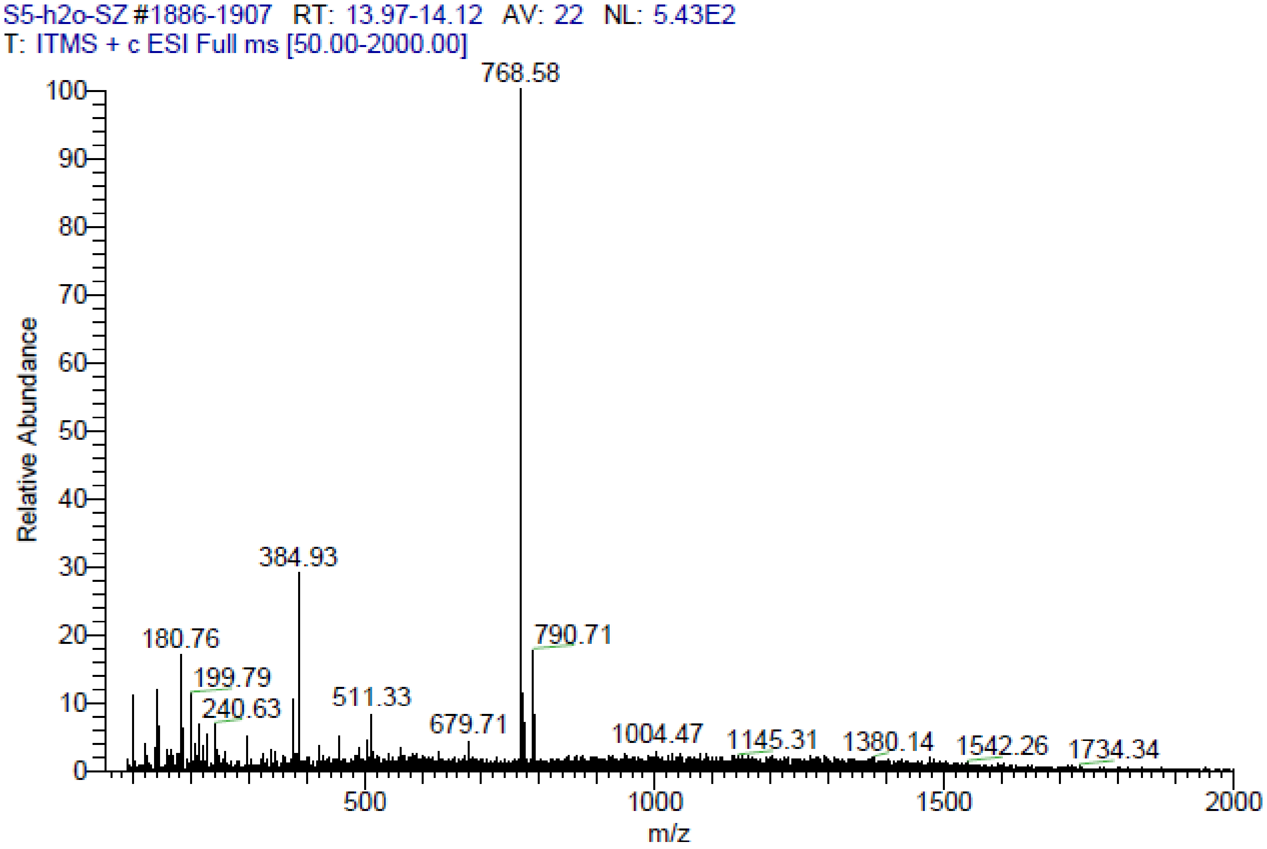

Figure S35. HPLC-MS chromatogram and mass spectrum for the diastereoselective chain elongation of **12** in the absence of water.

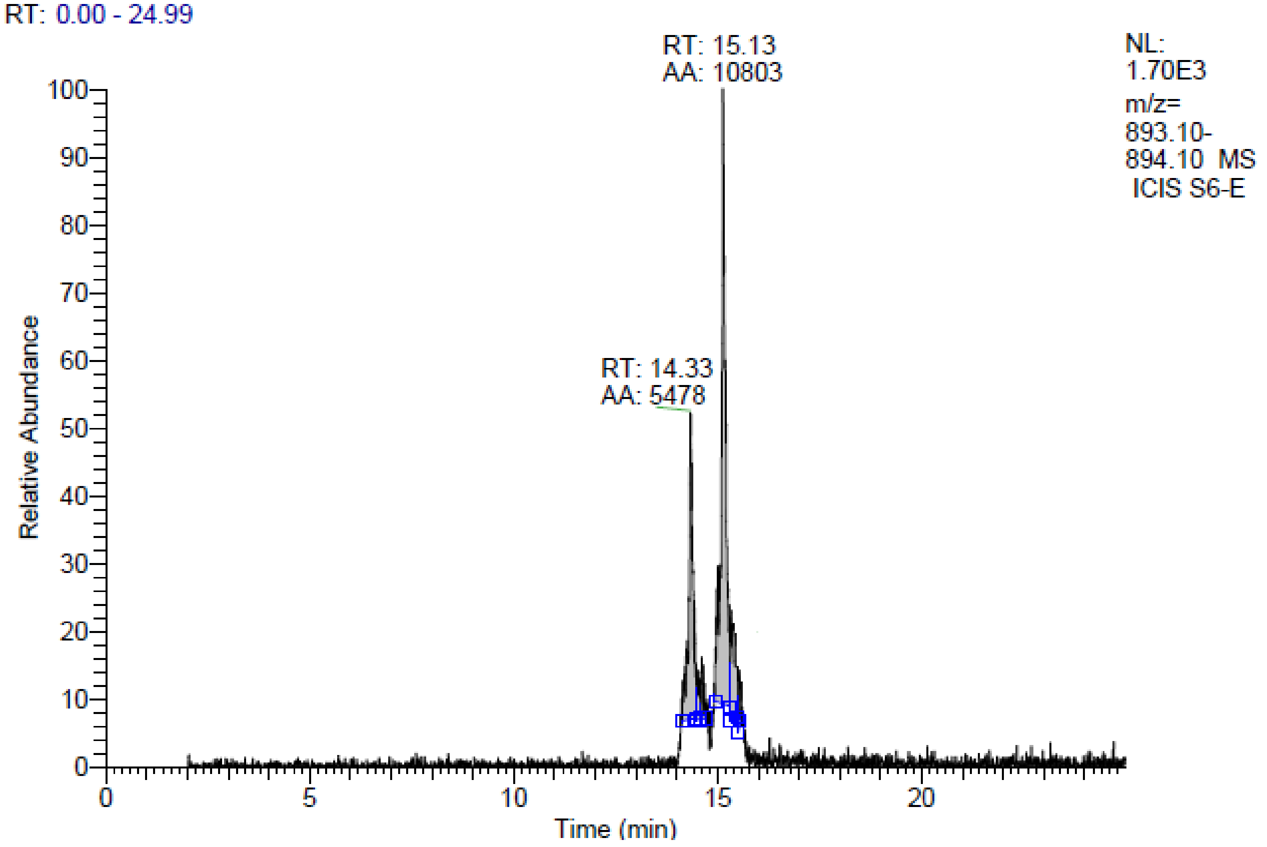

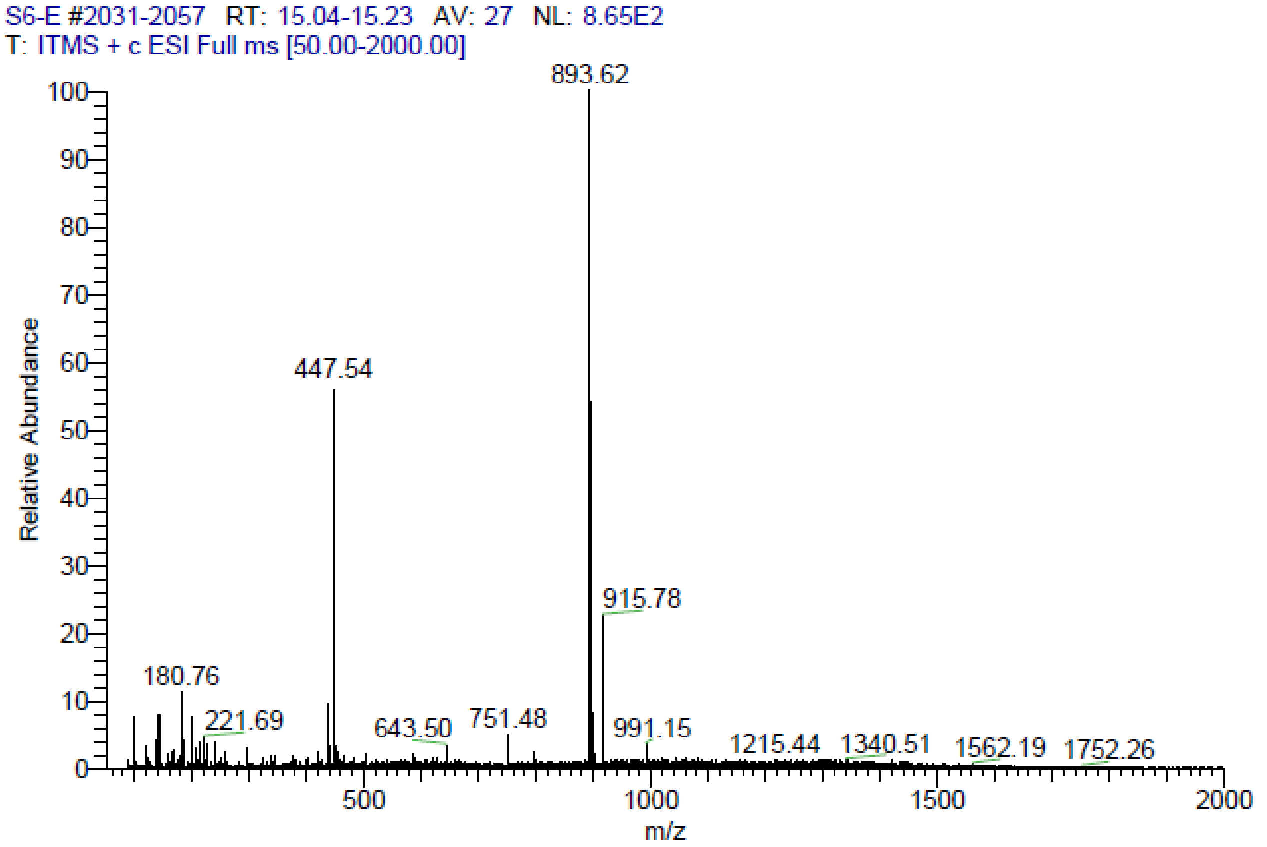

Figure S36. HPLC-MS chromatogram and mass spectrum for the diastereoselective chain elongation of **12** in the presence of water.

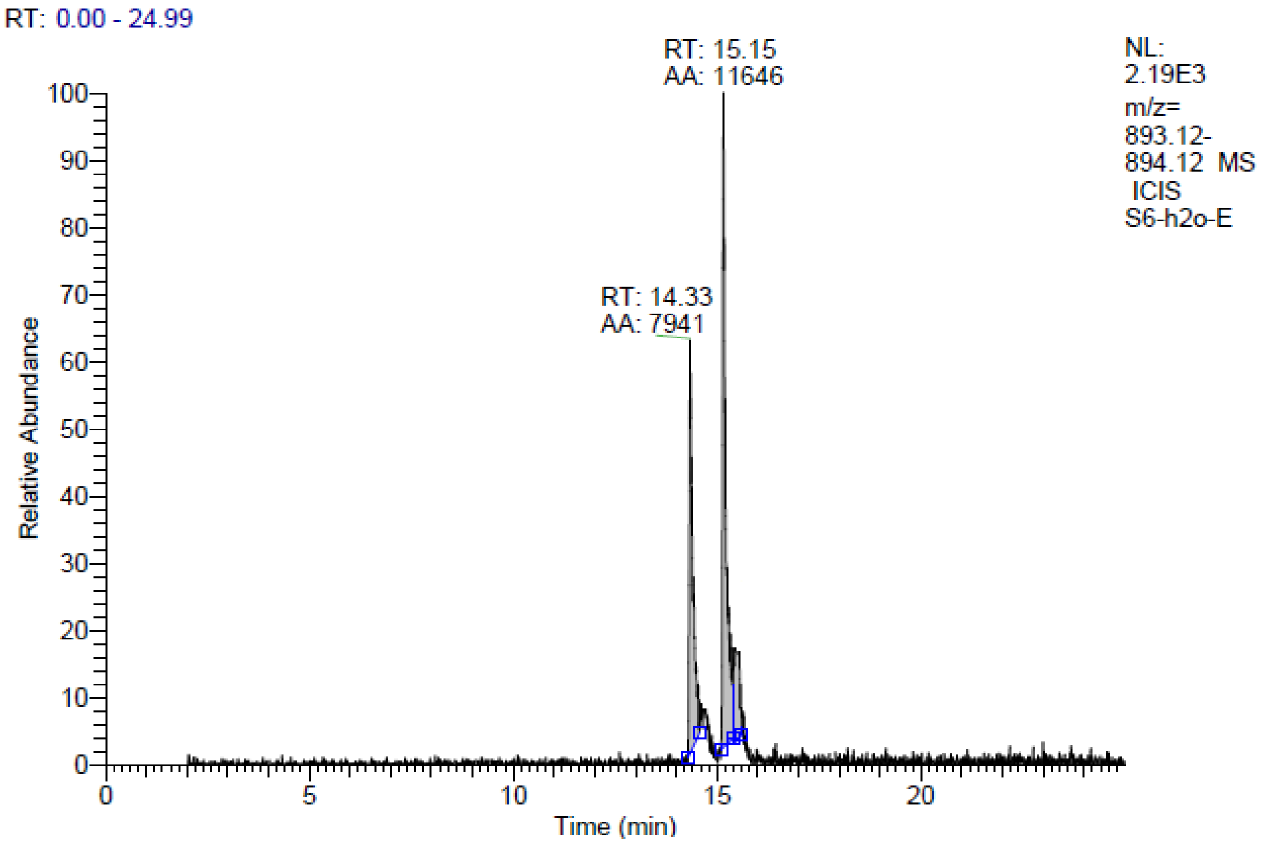

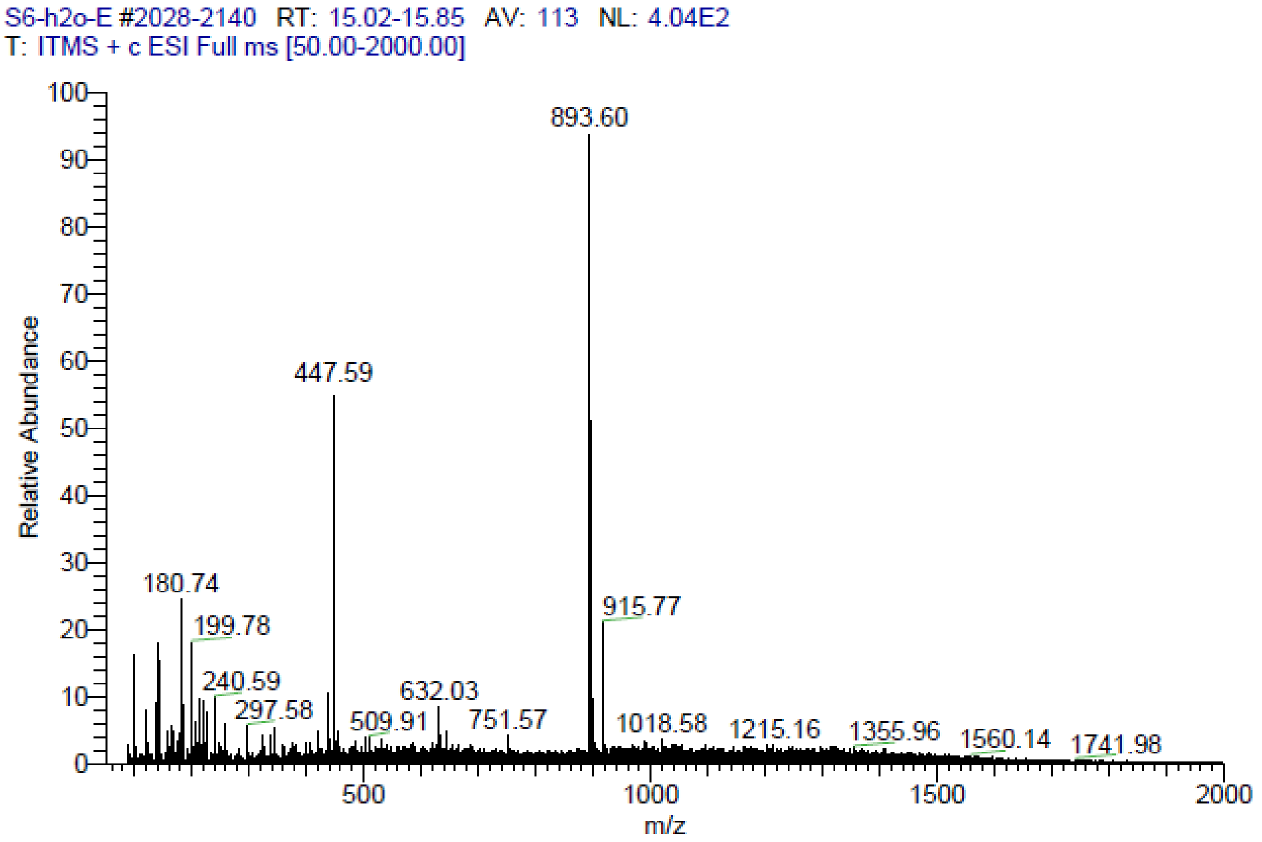

Figure S37. HPLC-MS chromatogram and mass spectrum of **13**.

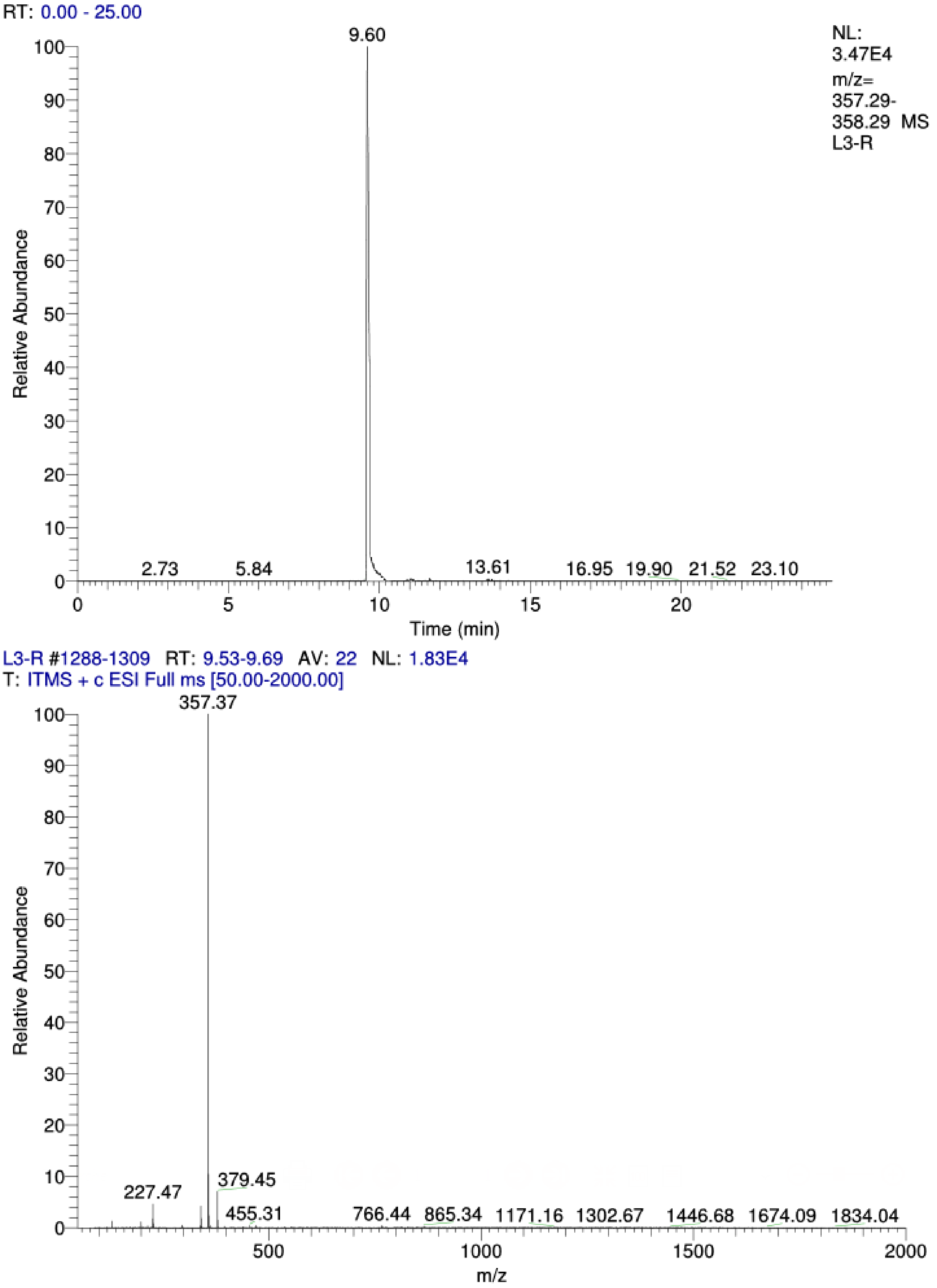

Figure S38. HPLC-MS chromatogram and mass spectrum of **14**.

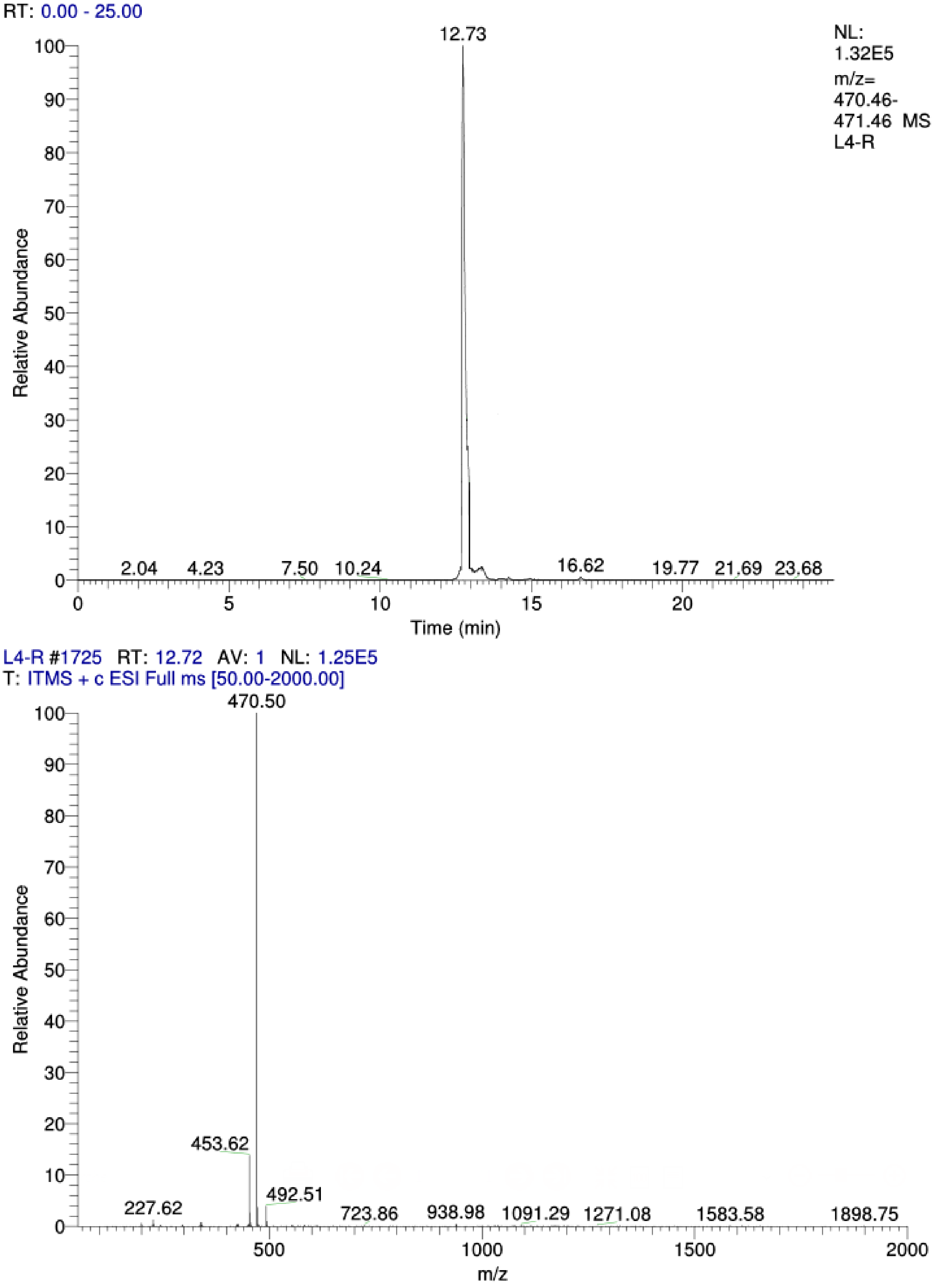

Figure S39. HPLC-MS chromatogram and mass spectrum of **15**.

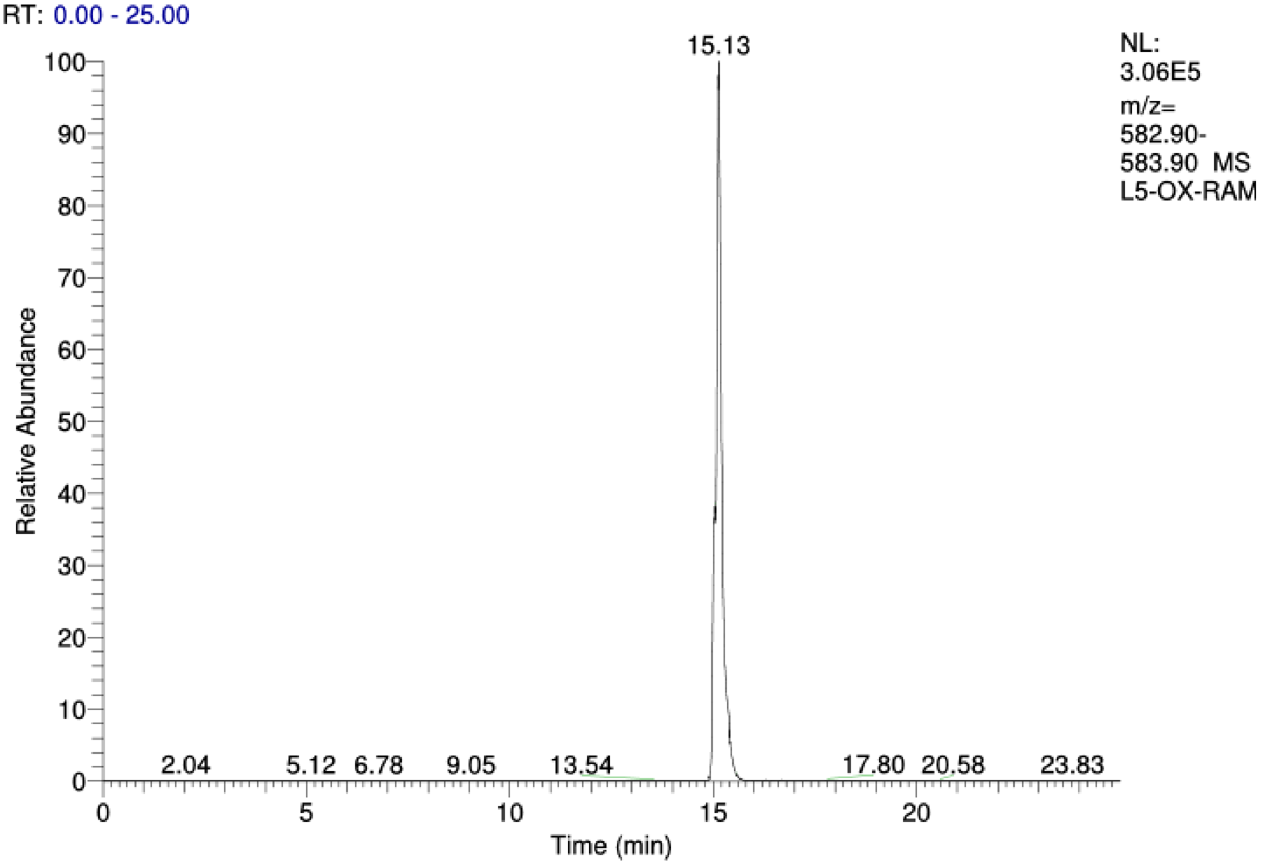

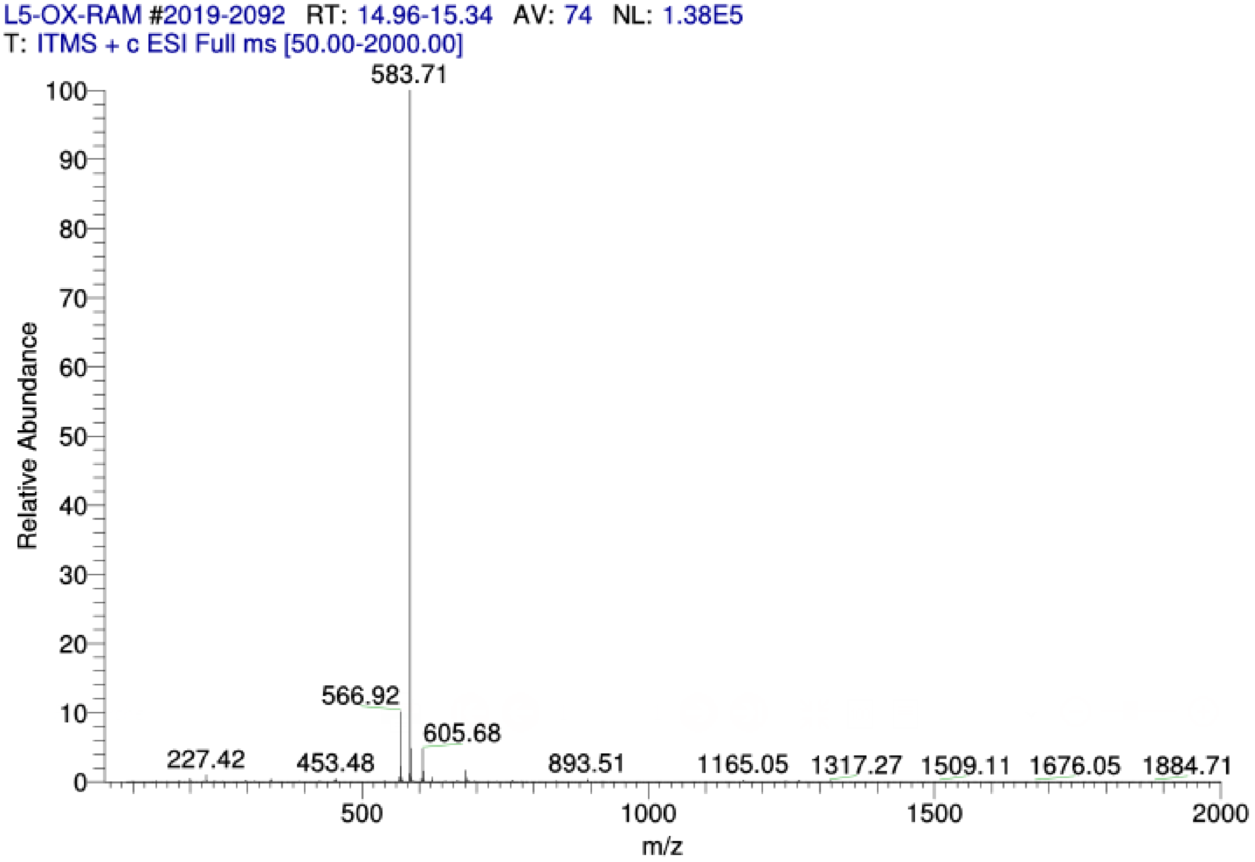

Figure S40. HPLC-MS chromatogram and mass spectrum of **16**.

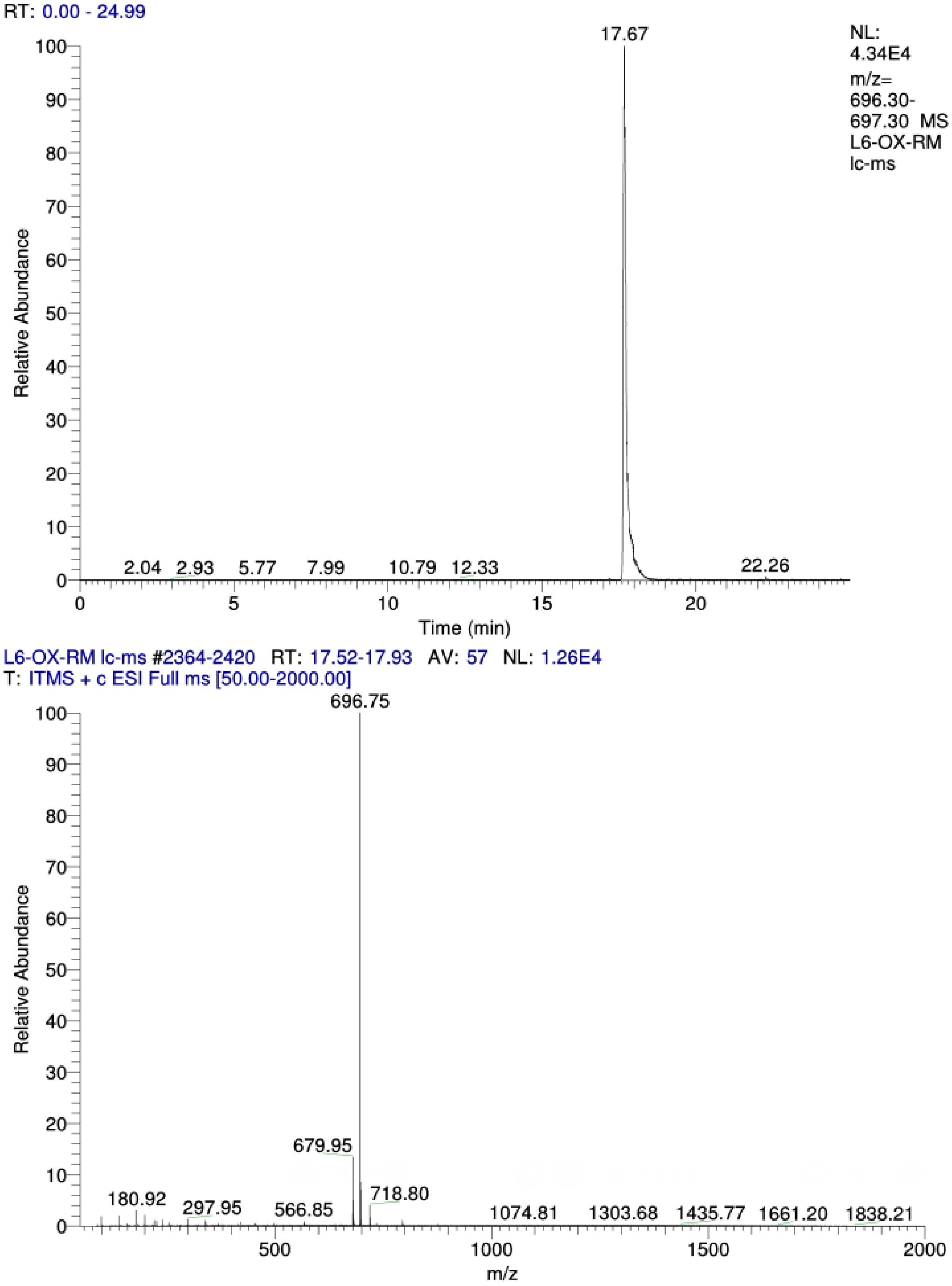

Figure S41. HPLC-MS chromatogram and mass spectrum for the diastereoselective chain elongation of **13** in the absence of water.

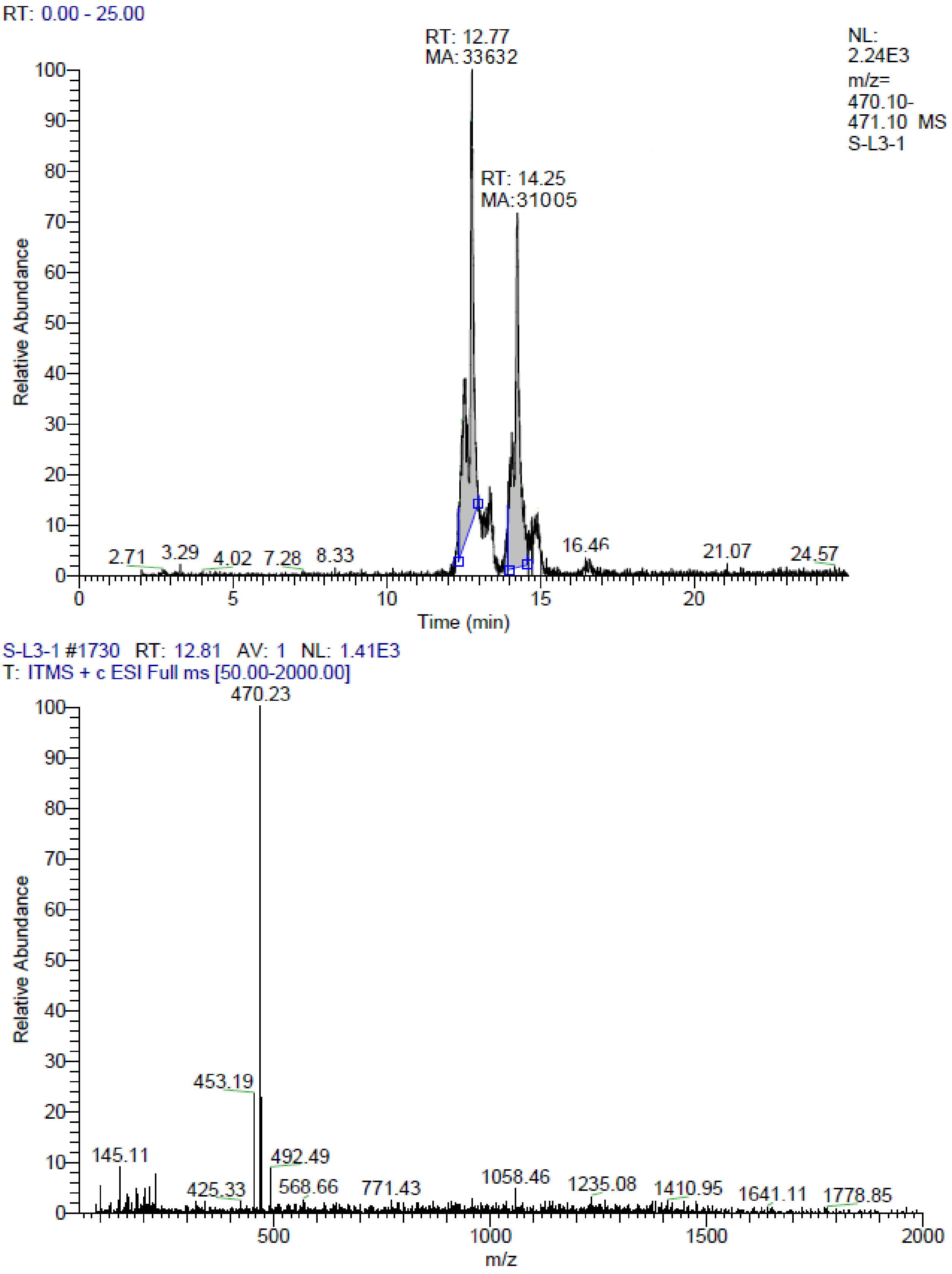

Figure S42. HPLC-MS chromatogram and mass spectrum for the diastereoselective chain elongation of **13** in the presence of water.

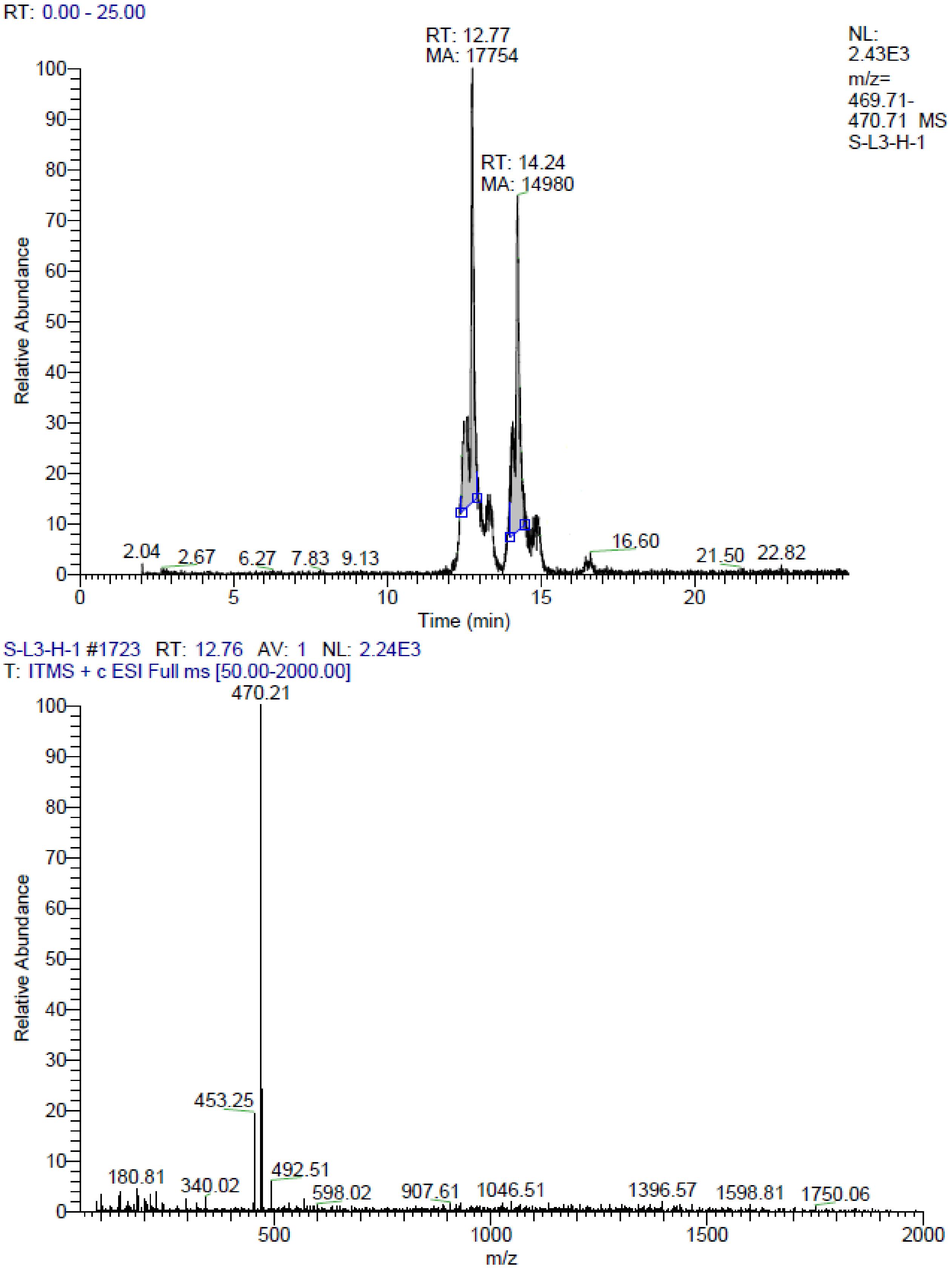

Figure S43. HPLC-MS chromatogram and mass spectrum for the diastereoselective chain elongation of **14** in the absence of water.

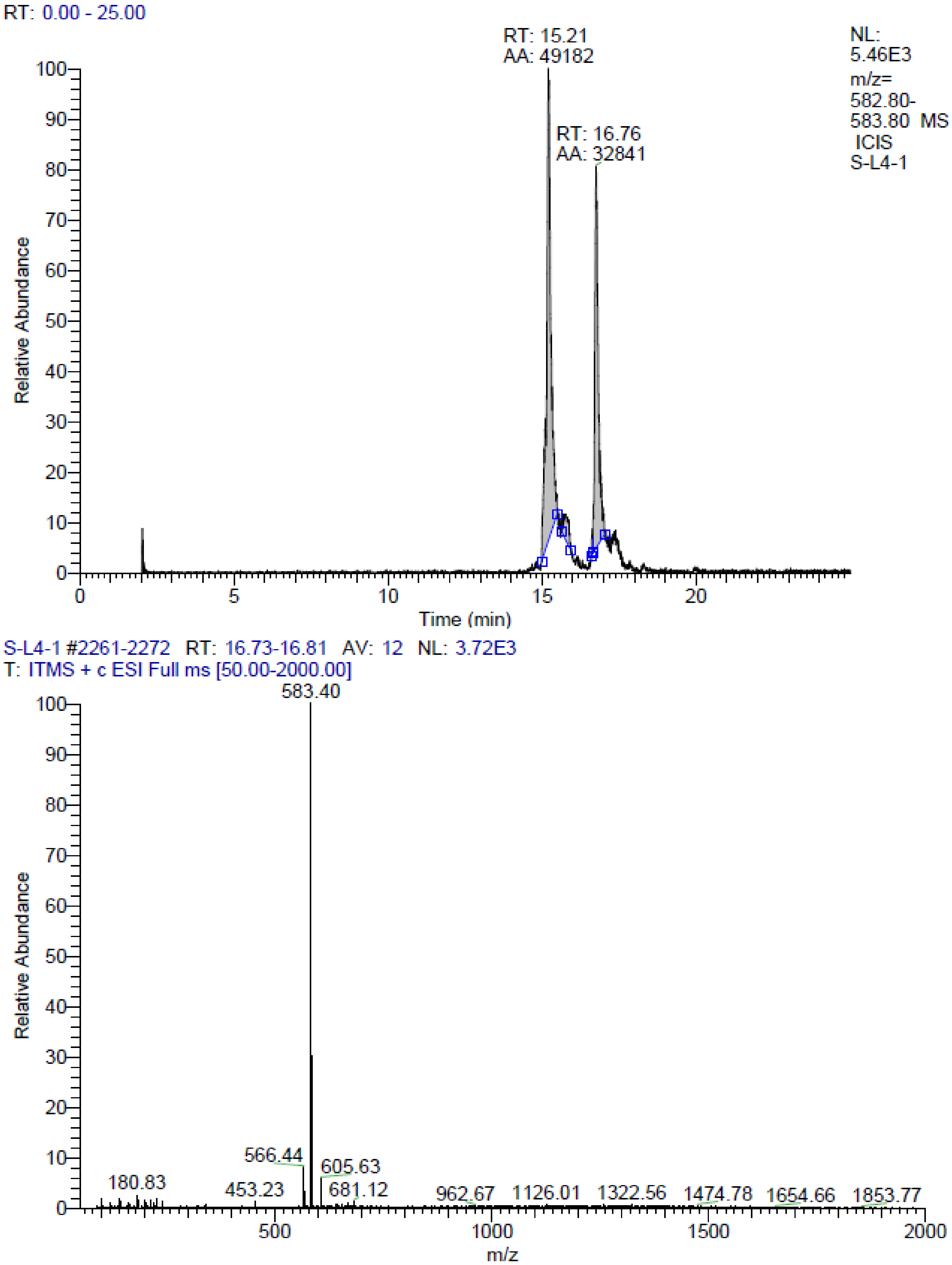

Figure S44. HPLC-MS chromatogram and mass spectrum for the diastereoselective chain elongation of **14** in the presence of water.

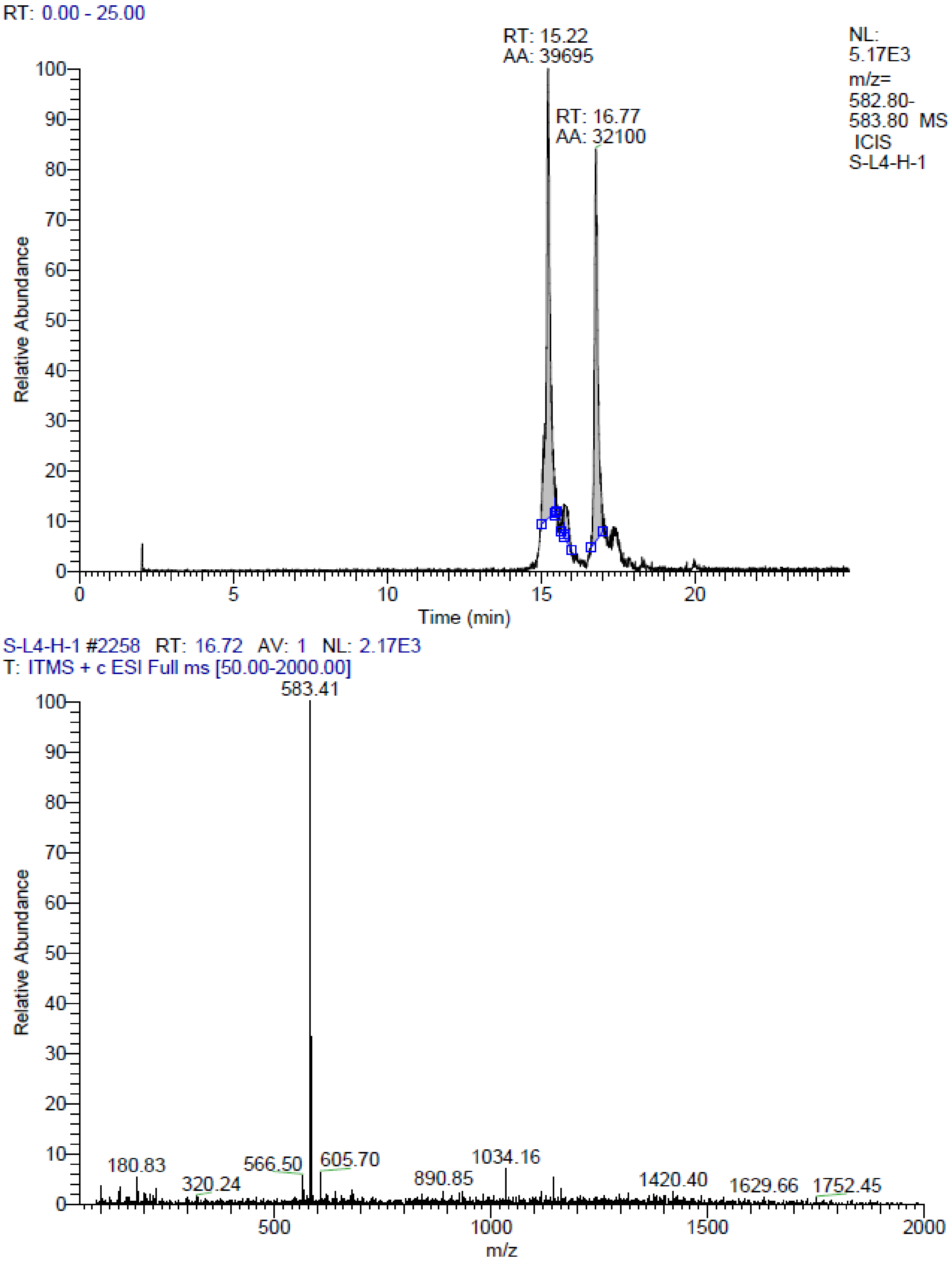

Figure S45. HPLC-MS chromatogram and mass spectrum for the diastereoselective chain elongation of **15** in the absence of water.

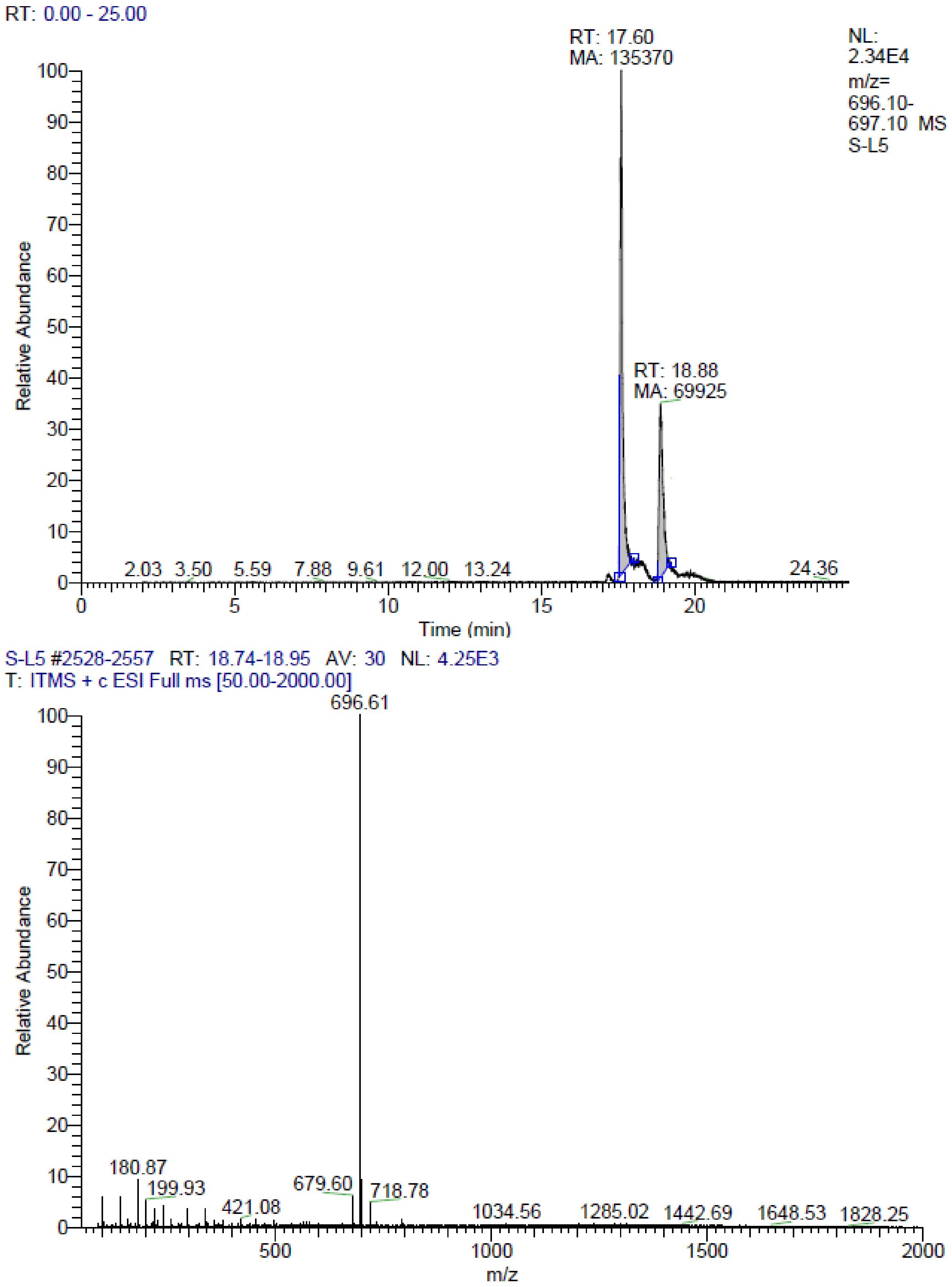

Figure S46. HPLC-MS chromatogram and mass spectrum for the diastereoselective chain elongation of **15** in the presence of water.

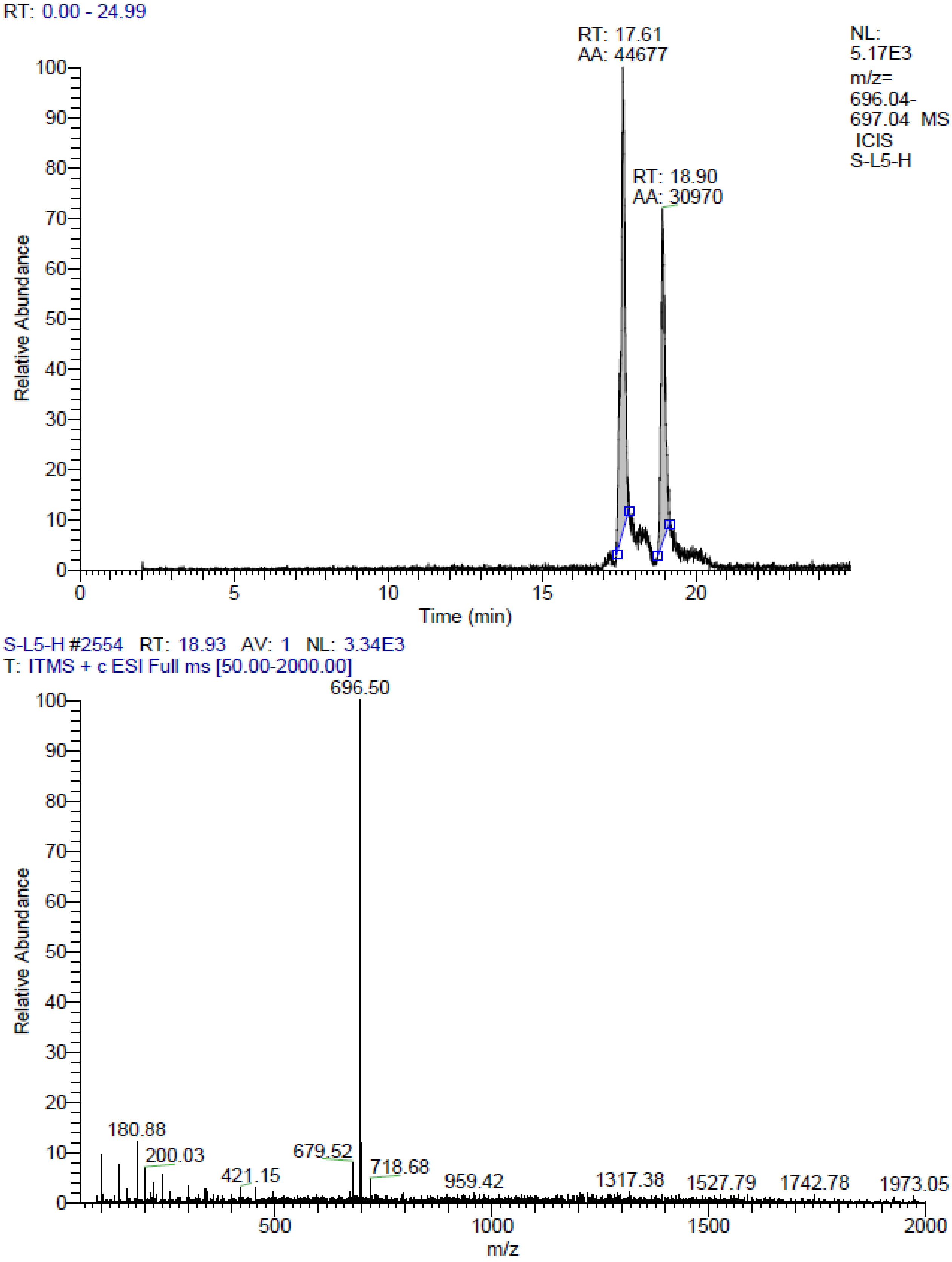

Figure S47. HPLC-MS chromatogram and mass spectrum for the diastereoselective chain elongation of **16** in the absence of water.

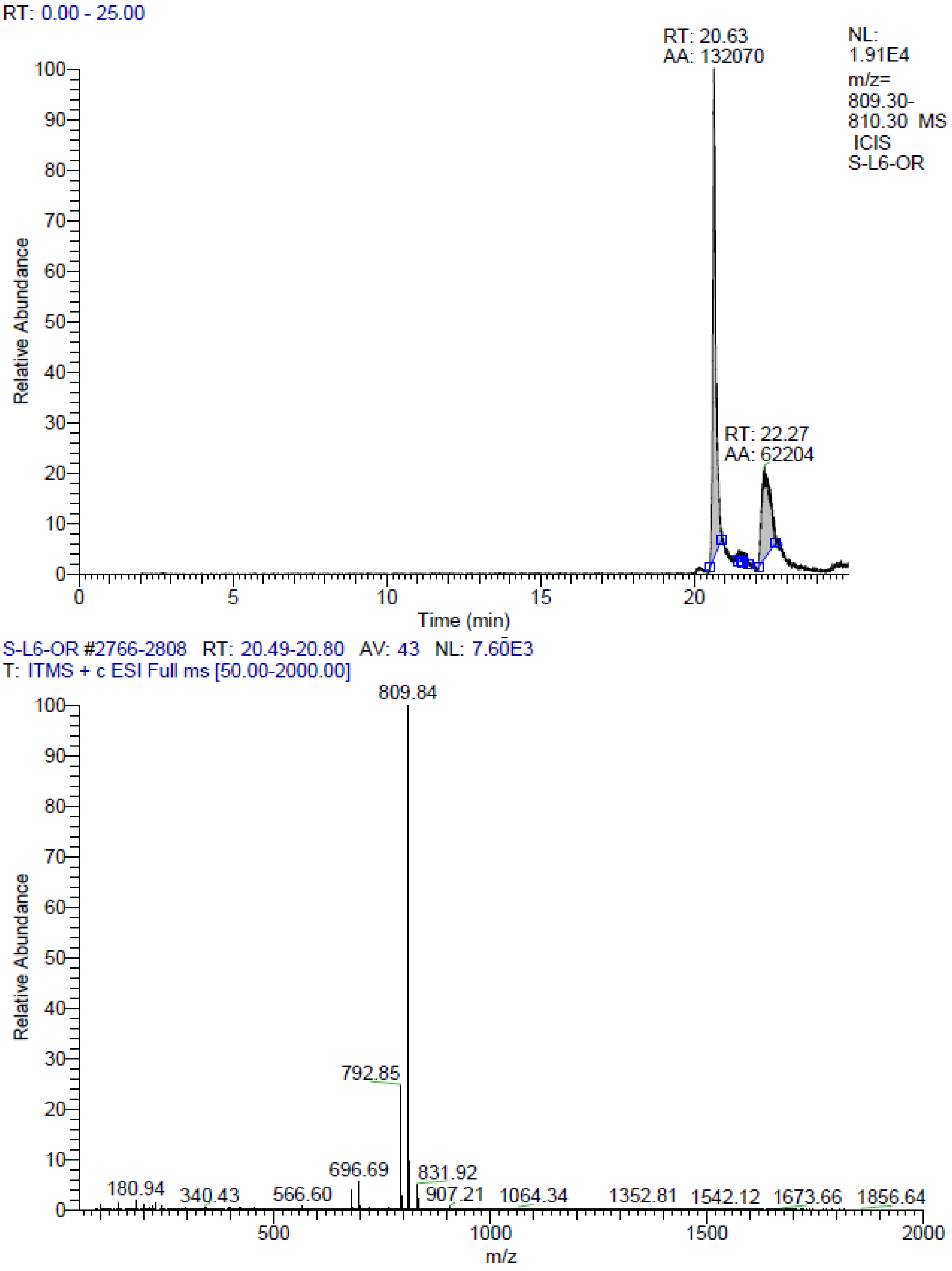

Figure S48. HPLC-MS chromatogram and mass spectrum for the diastereoselective chain elongation of **16** in the presence of water.

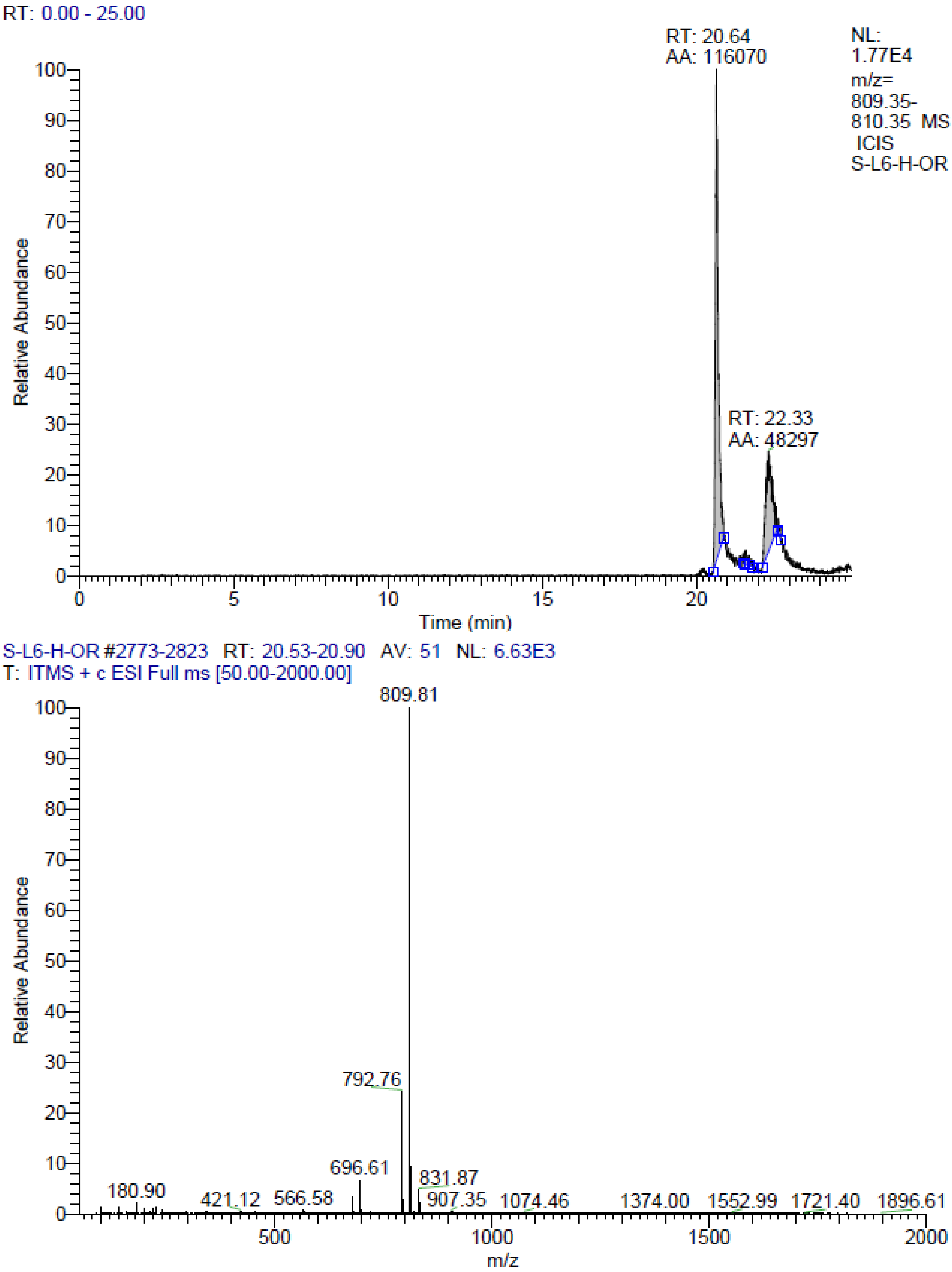

Table S5-S12 Optimized cartesian coordinates (in Å) and ADF total bonding energies (in kcal/mol) of the optimized minimum or transition state geometries calculated at OLYP/TZ2P level of theory in chloroform or water solvent.

S5. Minimized structure and energy of Boc-protected [1*R*,2*R*]-trans-ACHC hydroxybenzotriazole ester in chloroform (left, total bond energy: -6881.9 kcal/mol) and in water (right, total bond energy: -6885.9 kcal/mol)

1.C	-0.374350	-1.870817	-0.765867	1.C	-0.397482	-1.898091	-0.762999
2.C	0.199522	-4.303726	-1.282966	2.C	0.217978	-4.333152	-1.210491
3.C	-0.904378	-3.307846	-0.915158	3.C	-0.904657	-3.345909	-0.878933
4.C	0.792137	-1.832996	0.253834	4.C	0.756904	-1.816101	0.266874
5.C	1.909862	-2.835238	-0.130041	5.C	1.895683	-2.809727	-0.081577
6.C	1.356742	-4.253599	-0.283549	6.C	1.364057	-4.239301	-0.201789
7.H	-0.000651	-1.524396	-1.731857	7.H	-0.019354	-1.571922	-1.733947
8.H	-0.219960	-5.315234	-1.326936	8.H	-0.185985	-5.351623	-1.230972
9.H	0.574986	-4.077460	-2.289846	9.H	0.599948	-4.126716	-2.219140
10.H	-1.689425	-3.308008	-1.678884	10.H	-1.682313	-3.378122	-1.649572
11.H	-1.378196	-3.612144	0.027867	11.H	-1.382040	-3.631626	0.067702
12.H	0.406009	-2.106818	1.241262	12.H	0.366923	-2.073887	1.256701
13.C	1.381567	-0.445281	0.351033	13.C	1.326161	-0.420401	0.338056
14.H	2.369478	-2.509465	-1.069960	14.H	2.357992	-2.502748	-1.026664
15.H	2.692838	-2.812887	0.635078	15.H	2.670165	-2.755458	0.690127
16.H	1.018569	-4.623437	0.693539	16.H	1.021206	-4.585785	0.781867
17.H	2.167242	-4.917239	-0.605792	17.H	2.187691	-4.898584	-0.498641
18.N	-1.421367	-0.935954	-0.379215	18.N	-1.465283	-0.973635	-0.408641
19.C	-2.085456	-0.142514	-1.277378	19.C	-2.101729	-0.172338	-1.313915
20.O	-1.764411	0.002942	-2.448745	20.O	-1.736447	0.000368	-2.472306
21.O	-3.123482	0.459464	-0.637905	21.O	-3.173514	0.403738	-0.711160
22.C	-4.044894	1.404393	-1.314759	22.C	-4.041842	1.404947	-1.381916
23.H	-1.883945	-1.085333	0.506562	23.H	-1.948928	-1.129164	0.465029
24.C	-5.006127	1.784087	-0.182566	24.C	-5.051664	1.744651	-0.279977
25.C	-3.283290	2.639940	-1.806267	25.C	-3.229863	2.646828	-1.762878
26.C	-4.802189	0.695655	-2.442965	26.C	-4.754968	0.776105	-2.583249
27.H	-4.467563	2.253600	0.645340	27.H	-4.546866	2.156523	0.598332
28.H	-5.526976	0.901357	0.198672	28.H	-5.610030	0.855100	0.024736
29.H	-5.753406	2.491685	-0.551050	29.H	-5.763732	2.488034	-0.647309
30.H	-2.599656	2.397301	-2.619299	30.H	-2.510065	2.434795	-2.553249
31.H	-2.713456	3.092058	-0.989315	31.H	-2.695106	3.039469	-0.893064
32.H	-3.998927	3.384144	-2.168152	32.H	-3.910607	3.425942	-2.118090
33.H	-5.581530	1.360179	-2.827673	33.H	-5.504557	1.476659	-2.962861
34.H	-5.287079	-0.211091	-2.069148	34.H	-5.271416	-0.141410	-2.285928
35.H	-4.141644	0.428515	-3.267525	35.H	-4.061499	0.544761	-3.391461
36.O	1.403769	-0.058762	1.726758	36.O	1.439090	-0.046596	1.711399
37.O	1.806347	0.256402	-0.520175	37.O	1.680918	0.299289	-0.551976
38.H	-0.740167	2.176201	1.676760	38.H	-0.744409	2.135233	1.920938
39.N	2.003470	1.154086	1.958619	39.N	2.034733	1.172920	1.914723
40.N	3.621456	2.441142	2.510629	40.N	3.668767	2.483052	2.351434
41.C	2.471528	3.200676	2.441351	41.C	2.502630	3.217668	2.407190
42.C	1.392435	2.365434	2.086194	42.C	1.413358	2.366400	2.130260
43.H	0.761769	6.098233	2.689540	43.H	0.762568	6.066940	2.887262
44.C	0.967573	5.044048	2.525520	44.C	0.974954	5.022436	2.676800
45.C	-0.103513	4.187037	2.173130	45.C	-0.106342	4.148697	2.403455
46.H	-1.101334	4.607486	2.079613	46.H	-1.117362	4.546915	2.415764
47.N	3.323087	1.207381	2.224572	47.N	3.369683	1.248506	2.061097

48.H	3.086258	5.224240	2.935233	48.H	3.117325	5.243973	2.895744
49.C	2.261724	4.571042	2.664593	49.C	2.286161	4.577555	2.684248
50.C	0.080990	2.831962	1.945239	50.C	0.084513	2.804441	2.123990

S6. Minimized structure and energy of Boc-protected [1*S*,2*S*]-trans-ACHC hydroxybenzotriazole ester in chloroform (left, total bond energy: -6882.0 kcal/mol) and in water (right, total bond energy: -6885.9 kcal/mol)

1.C	-0.273927	-1.694712	0.750348	1.C	-0.327772	-1.765918	0.718944
2.C	-0.186965	-4.211139	0.332865	2.C	-0.186045	-4.261358	0.206986
3.C	-1.046005	-2.943894	0.291132	3.C	-1.068089	-3.009611	0.197403
4.C	1.026211	-1.531805	-0.076053	4.C	0.980278	-1.548342	-0.081552
5.C	1.894911	-2.814633	-0.023107	5.C	1.875183	-2.814810	-0.055278
6.C	1.101392	-4.042513	-0.474647	6.C	1.113722	-4.038641	-0.568254
7.C	3.988202	4.810241	0.211661	7.C	3.906678	4.818003	0.262192
8.H	0.062514	-4.449308	1.375491	8.H	0.048607	-4.534765	1.244356
9.H	-0.766372	-5.058807	-0.050264	9.H	-0.742379	-5.103181	-0.220547
10.H	-1.413466	-2.779660	-0.730774	10.H	-1.419180	-2.811122	-0.823842
11.H	-1.927769	-3.058364	0.930625	11.H	-1.959011	-3.164778	0.815309
12.C	1.445395	3.480419	-0.039076	12.C	1.387862	3.470104	-0.116622
13.C	1.842289	-0.361345	0.420485	13.C	1.765288	-0.378321	0.458874
14.H	2.778282	-2.678660	-0.655600	14.H	2.767376	-2.638696	-0.664484
15.H	2.252902	-2.957610	1.002755	15.H	2.213949	-2.989529	0.972336
16.H	1.734664	-4.931062	-0.371676	16.H	1.761914	-4.918504	-0.485585
17.H	0.859988	-3.951247	-1.541988	17.H	0.891384	-3.909635	-1.635584
18.N	-1.084704	-0.488483	0.667956	18.N	-1.163427	-0.574631	0.667794
19.C	-1.733447	0.056544	1.743814	19.C	-1.804074	-0.052878	1.755577
20.O	-1.569769	-0.290315	2.905611	20.O	-1.615514	-0.407448	2.914765
21.O	-2.554658	1.044900	1.299405	21.O	-2.652457	0.922275	1.338216
22.C	-3.364663	1.886749	2.213126	22.C	-3.446270	1.761321	2.272023
23.H	-1.421612	-0.208213	-0.242388	23.H	-1.508464	-0.281624	-0.235788
24.C	-2.454719	2.683220	3.154227	24.C	-2.518600	2.563866	3.189760
25.C	-4.079668	2.827927	1.237065	25.C	-4.188429	2.697353	1.311691
26.C	-4.384083	1.027626	2.968918	26.C	-4.443587	0.897318	3.050651
27.H	-1.707769	3.243947	2.584560	27.H	-1.785765	3.124316	2.601758
28.H	-1.941483	2.036548	3.865396	28.H	-1.989304	1.922363	3.894042
29.H	-3.057777	3.402661	3.716044	29.H	-3.112492	3.283928	3.760256
30.H	-4.701611	2.263536	0.536709	30.H	-4.823971	2.128642	0.627176
31.H	-3.358271	3.416828	0.663723	31.H	-3.483656	3.289918	0.721713
32.H	-4.723356	3.517201	1.789794	32.H	-4.822614	3.382972	1.879562
33.H	-5.068304	1.679742	3.519963	33.H	-5.119658	1.547461	3.613786
34.H	-3.900638	0.358103	3.679833	34.H	-3.941967	0.232918	3.753893
35.H	-4.977468	0.432053	2.268716	35.H	-5.047802	0.296627	2.364349
36.O	2.188738	0.454609	-0.701103	36.O	2.177287	0.433515	-0.641290
37.O	2.204486	-0.110733	1.533023	37.O	2.064978	-0.122756	1.590515
38.H	0.487976	2.980109	-0.136466	38.H	0.440488	2.964087	-0.267284
39.N	3.019488	1.503690	-0.395836	39.N	2.984420	1.488047	-0.294511
40.N	4.909339	2.494006	-0.229669	40.N	4.856834	2.489958	-0.033566
41.C	3.914051	3.431809	-0.045490	41.C	3.851290	3.430641	0.049943
42.C	2.660265	2.797031	-0.160532	42.C	2.609356	2.787800	-0.129311
43.H	2.804227	6.564502	0.533258	43.H	2.700607	6.577410	0.438207
44.C	2.791552	5.496160	0.335594	44.C	2.703243	5.502719	0.278915
45.C	1.542258	4.840894	0.209881	45.C	1.466068	4.838585	0.090595
46.H	0.631618	5.425239	0.311913	46.H	0.550076	5.423020	0.107662

47.N	4.355375	1.337135	-0.448577	47.N	4.318990	1.323672	-0.253015
48.H	4.948665	5.308566	0.306185	48.H	4.857066	5.324130	0.404564
49.H	0.758729	-1.339370	-1.120316	49.H	0.724788	-1.330860	-1.123512
50.H	-0.003077	-1.807447	1.802341	50.H	-0.070879	-1.916139	1.769458

S7. Minimized structure and energy of **7** in chloroform (total bond energy: -11 068.2 kcal/mol).

1.C	17.337176	0.022645	3.719984	43.H	20.789020	1.496053	2.512919
2.C	18.560805	-0.156214	4.622416	44.H	20.500235	-0.237701	2.553885
3.C	19.679819	0.820929	4.252834	45.H	16.576048	-0.727740	3.966556
4.H	20.022166	-3.597862	0.883063	46.H	16.882699	1.003547	3.903923
5.H	19.265255	-6.536100	0.580673	47.H	18.931389	-1.187259	4.536359
6.H	19.462804	-5.194288	-1.522257	48.H	18.267344	-0.018539	5.670529
7.H	15.845333	-5.842520	0.839205	49.H	19.359563	1.844622	4.491112
8.H	17.093042	-6.991715	0.514346	50.H	20.570958	0.625856	4.862149
9.H	18.645804	-1.210801	-3.215123	51.N	19.698596	-4.372161	-0.985986
10.H	18.928205	-4.243027	-3.337398	52.C	20.126272	-4.570365	0.396667
11.H	16.841201	-3.345826	-2.357084	53.C	19.202420	-5.578106	1.114043
12.H	14.671407	0.032089	-0.464847	54.C	17.738647	-5.114833	1.127686
13.H	15.218518	-2.909249	-1.043686	55.O	17.404195	-3.984166	1.486016
14.H	16.464483	-1.862554	0.902652	56.N	16.829286	-6.047624	0.745283
15.H	18.474818	1.122921	-0.163478	57.N	17.082745	-2.399290	-2.608318
16.H	19.529968	-0.058200	0.181239	58.C	18.256260	-2.202931	-3.458016
17.H	18.422275	1.933369	2.017512	59.C	19.344379	-3.248126	-3.127333
18.H	18.095633	-1.093251	2.041823	60.C	19.771085	-3.186131	-1.651611
19.H	22.221765	-4.257179	-0.002653	61.O	20.160354	-2.141710	-1.125846
20.H	21.719947	-5.942171	-0.106435	62.N	15.996756	-0.984009	0.722260
21.H	19.034853	-6.570545	3.035885	63.C	14.813006	-0.998875	-0.132996
22.H	19.533823	-4.887244	3.135527	64.C	15.033894	-1.880977	-1.381988
23.H	21.252324	-7.243736	2.188313	65.C	16.236304	-1.409913	-2.218007
24.H	21.438989	-6.346027	3.689184	66.O	16.395871	-0.227484	-2.531040
25.H	22.062726	-4.301567	2.461166	67.N	19.256916	0.887464	0.442196
26.H	23.089777	-5.620289	1.919071	68.C	18.825215	0.928003	1.846404
27.H	17.148577	-1.468657	-5.156312	69.C	17.705574	-0.084457	2.223189
28.H	17.416475	-3.209937	-5.176456	70.C	16.456823	0.123557	1.359438
29.H	21.314808	-3.831581	-3.818706	71.O	15.911027	1.230667	1.259782
30.H	21.047181	-2.093236	-3.785929	72.C	21.597497	-5.018704	0.475818
31.H	19.871806	-4.080863	-5.803396	73.C	19.675124	-5.814273	2.567750
32.H	21.111120	-2.873482	-6.121030	74.C	21.141257	-6.247756	2.638572
33.H	19.514082	-1.039322	-5.718869	75.C	22.055918	-5.255913	1.917270
34.H	18.821975	-2.139101	-6.903089	76.C	17.892681	-2.245343	-4.954068
35.H	13.416299	-0.769859	1.492341	77.C	20.577180	-3.050750	-4.037799
36.H	13.735737	-2.446487	1.062273	78.C	20.213153	-3.074352	-5.524541
37.H	13.923918	-2.561130	-3.118754	79.C	19.119035	-2.055642	-5.851040
38.H	13.611550	-0.888202	-2.673074	80.C	13.559920	-1.446335	0.643333
39.H	12.615329	-3.382321	-1.194446	81.C	13.763563	-1.893973	-2.263962
40.H	11.641245	-2.266417	-2.142597	82.C	12.517936	-2.327346	-1.486605
41.H	12.056964	-0.442315	-0.539389	83.C	12.308104	-1.468233	-0.237948
42.H	11.454696	-1.839792	0.341676	84.C	20.039392	0.737900	2.766441

S8. Minimized structure and energy of **7** in water (total bond energy: -11 077.2 kcal/mol).

1.C	17.285442	0.048426	3.750131	43.H	20.801995	1.413849	2.604417
2.C	18.482362	-0.145571	4.684620	44.H	20.467893	-0.311996	2.666600
3.C	19.634502	0.796499	4.326594	45.H	16.500189	-0.678497	3.990012
4.H	20.047182	-3.558845	0.863150	46.H	16.853067	1.043923	3.907713
5.H	19.240479	-6.484366	0.572446	47.H	18.827937	-1.186857	4.625552
6.H	19.426822	-5.163166	-1.521341	48.H	18.166856	0.018628	5.722165
7.H	15.844001	-5.705408	0.721367	49.H	19.336026	1.832092	4.540228
8.H	17.085456	-6.847635	0.345726	50.H	20.505686	0.587736	4.959220
9.H	18.640358	-1.216800	-3.267811	51.N	19.698314	-4.340513	-1.002612
10.H	18.918295	-4.250983	-3.342815	52.C	20.131903	-4.534653	0.379527
11.H	16.817281	-3.346789	-2.405797	53.C	19.196174	-5.526306	1.105300
12.H	14.669529	0.021894	-0.447800	54.C	17.740412	-5.041588	1.122827
13.H	15.242424	-2.912128	-1.037336	55.O	17.420334	-3.925452	1.548974
14.H	16.441193	-1.873117	0.957938	56.N	16.826903	-5.930008	0.671312
15.H	18.537556	1.039679	-0.122278	57.N	17.084399	-2.397651	-2.621966
16.H	19.551366	-0.158432	0.270869	58.C	18.248180	-2.212637	-3.489045
17.H	18.462494	1.902228	2.041447	59.C	19.339471	-3.254581	-3.156181
18.H	18.059753	-1.115443	2.113144	60.C	19.785869	-3.173382	-1.688327
19.H	22.231237	-4.260775	-0.035702	61.O	20.204716	-2.123448	-1.186751
20.H	21.694472	-5.935306	-0.133626	62.N	15.995112	-0.989021	0.746655
21.H	19.024172	-6.513780	3.026110	63.C	14.818324	-1.008980	-0.120546
22.H	19.556319	-4.839159	3.124271	64.C	15.055749	-1.883727	-1.371563
23.H	21.221444	-7.226709	2.162812	65.C	16.260983	-1.408334	-2.198037
24.H	21.436986	-6.333102	3.663103	66.O	16.438157	-0.215120	-2.475490
25.H	22.088021	-4.300203	2.430882	67.N	19.302887	0.801545	0.503904
26.H	23.086985	-5.637722	1.879467	68.C	18.840479	0.882696	1.899262
27.H	17.117461	-1.507490	-5.187210	69.C	17.688425	-0.095104	2.264538
28.H	17.380632	-3.249214	-5.173993	70.C	16.466572	0.118458	1.366043
29.H	21.296037	-3.858378	-3.864177	71.O	15.948069	1.239911	1.228465
30.H	21.037668	-2.117204	-3.864160	72.C	21.595588	-5.009469	0.448472
31.H	19.821720	-4.141411	-5.818413	73.C	19.674855	-5.768495	2.555093
32.H	21.060232	-2.945547	-6.182215	74.C	21.132981	-6.229492	2.614755
33.H	19.476415	-1.097209	-5.791935	75.C	22.060021	-5.254219	1.886747
34.H	18.761575	-2.219284	-6.942282	76.C	17.862477	-2.282086	-4.978542
35.H	13.408889	-0.804458	1.499232	77.C	20.559612	-3.078096	-4.087663
36.H	13.749253	-2.474605	1.059625	78.C	20.171461	-3.130862	-5.567312
37.H	13.964439	-2.563667	-3.118924	79.C	19.076052	-2.114771	-5.897255
38.H	13.630617	-0.895264	-2.667090	80.C	13.565983	-1.473758	0.646527
39.H	12.655302	-3.408219	-1.208390	81.C	13.791380	-1.902015	-2.262958
40.H	11.673317	-2.297721	-2.155586	82.C	12.546613	-2.353285	-1.494865
41.H	12.058535	-0.478648	-0.538953	83.C	12.320022	-1.503685	-0.242885
42.H	11.468917	-1.889344	0.330524	84.C	20.027468	0.678507	2.850932

S9. Transition state optimized structure and energy of Boc-protected [1*R*,2*R*]-trans-ACHC hydroxybenzotriazole ester and **7** complex in chloroform (total bond energy: -17 899.2 kcal/mol).

1.C	-4.217768	-2.552774	0.007406
2.C	-4.146604	-5.109284	0.223487
3.C	-4.861376	-3.818247	0.620976
4.C	-2.736907	-2.471012	0.477393
5.C	-2.018251	-3.768997	-0.004927
6.C	-2.660229	-5.036031	0.566986
7.H	-4.218480	-2.656246	-1.085170
8.H	-4.616281	-5.962608	0.727240
9.H	-4.266044	-5.278824	-0.855421
10.H	-5.906475	-3.852275	0.305242
11.H	-4.860177	-3.708013	1.713699
12.H	-2.717928	-2.426766	1.569592
13.C	-1.941229	-1.294270	-0.070183
14.H	-2.065174	-3.795026	-1.100118
15.H	-0.960384	-3.751650	0.257569
16.H	-2.529783	-5.055631	1.657733
17.H	-2.126047	-5.909069	0.172999
18.N	-4.995270	-1.370576	0.366582
19.C	-6.198317	-1.100829	-0.215424
20.O	-6.764672	-1.824370	-1.029600
21.O	-6.671068	0.083840	0.266620
22.C	-7.997983	0.627806	-0.091115
23.H	-4.501135	-0.601817	0.812812
24.C	-8.043600	1.930016	0.720143
25.C	-8.079653	0.934722	-1.592287
26.C	-9.109033	-0.325155	0.369417
27.H	-7.228629	2.598184	0.428070
28.H	-7.954613	1.724014	1.790804
29.H	-8.992423	2.445457	0.546162
30.H	-8.061890	0.024666	-2.191879
31.H	-7.248518	1.576552	-1.898313
32.H	-9.012125	1.468385	-1.801913
33.H	-10.081694	0.158930	0.236207
34.H	-8.993896	-0.563250	1.431535
35.H	-9.105823	-1.253723	-0.201962
36.O	-2.808446	0.236199	1.135647
37.O	-1.879336	-0.886181	-1.190455
38.H	-3.969161	1.125288	-1.611406
39.N	-2.504620	1.499651	0.902952
40.N	-1.732395	3.471579	1.427638
41.C	-2.360955	3.573906	0.213184
42.C	-2.872105	2.307571	-0.147373
43.H	-3.425485	5.320024	-2.480397
44.C	-3.256145	4.484771	-1.804455
45.C	-3.765638	3.206856	-2.152112
46.H	-4.312761	3.094970	-3.085497
47.N	-1.823294	2.223301	1.829022
48.H	-2.161218	5.663193	-0.356337
49.C	-2.552844	4.685780	-0.628543
50.C	-3.584974	2.100640	-1.337930
51.O	1.511903	1.330663	-0.292831
52.N	3.362341	-2.301179	0.330743
53.C	4.038576	-3.043686	-0.734127
54.C	4.457148	-2.109835	-1.894816
55.C	3.241557	-1.358526	-2.463822
56.O	2.196846	-1.943481	-2.761301
57.N	-0.367784	-1.156191	0.677045
58.C	0.111851	-1.708955	2.003425
59.C	1.659416	-1.802724	2.026316
60.C	2.199717	-2.703084	0.898755
61.O	1.597146	-3.731490	0.566557
62.C	2.615731	4.054491	1.918385
63.C	4.864110	2.818063	3.385851
64.C	4.138317	4.045433	3.944593
65.C	2.692106	4.119984	3.447619
66.C	2.418098	1.100122	-4.613854
67.C	1.321505	3.188753	-2.835146
68.C	1.354908	3.385057	-4.354528
69.C	1.316002	2.047811	-5.099890
70.C	5.242507	-3.840525	-0.199264
71.C	5.154938	-2.924619	-3.008676
72.C	6.344521	-3.732012	-2.478999
73.C	5.939623	-4.634534	-1.309864
74.C	-0.412499	-0.887079	3.182871
75.C	2.126857	-2.376197	3.389305
76.C	1.584748	-1.580935	4.578721
77.C	0.061034	-1.465722	4.520811
78.H	2.819908	1.917544	1.696909
79.H	5.327329	3.635853	1.446990
80.H	3.866127	3.409537	-0.616673
81.H	7.284379	0.901716	0.489233
82.H	7.126593	2.614470	0.660981
83.H	1.414825	0.412841	-2.831766
84.H	3.407197	2.708145	-2.590161
85.H	4.306793	0.375720	-2.386495
86.H	3.293534	-3.743567	-1.118180
87.H	5.180121	-1.380963	-1.504379
88.H	3.808191	-1.464064	0.685964
89.H	0.196375	-1.565453	-0.069059
90.H	-0.185639	-0.151654	0.623987
91.H	-0.276693	-2.726563	2.060763
92.H	2.071806	-0.794160	1.916508
93.H	1.571802	4.031171	1.590698
94.H	3.069236	4.958160	1.487403
95.H	5.912913	2.820915	3.705100
96.H	4.414343	1.903339	3.790716
97.H	4.676841	4.956076	3.647253
98.H	4.163435	4.012865	5.040694
99.H	2.113617	3.290499	3.877349
100.H	2.215395	5.042342	3.799991
101.H	2.327453	0.126226	-5.106439
102.H	3.403105	1.504675	-4.885018
103.H	1.417517	4.156437	-2.329310
104.H	0.354266	2.771477	-2.533158
105.H	2.265973	3.933040	-4.633541
106.H	0.508731	4.013672	-4.658069
107.H	0.336474	1.573257	-4.949370
108.H	1.418860	2.210403	-6.179541
109.H	4.894574	-4.516780	0.589629
110.H	5.957042	-3.149243	0.268040
111.H	5.485637	-2.243465	-3.801686
112.H	4.419959	-3.601757	-3.460465
113.H	7.139154	-3.045429	-2.154452
114.H	6.766880	-4.333349	-3.293387
115.H	5.263510	-5.420772	-1.673179
116.H	6.819355	-5.145749	-0.900659
117.H	-1.502273	-0.846384	3.152100
118.H	-0.068586	0.148926	3.086160
119.H	3.221894	-2.388720	3.408696
120.H	1.797584	-3.420114	3.462282
121.H	2.030489	-0.576788	4.585431
122.H	1.896403	-2.066656	5.511135
123.H	-0.387965	-2.456877	4.673950
124.H	-0.307339	-0.830262	5.334220
125.N	3.272548	2.771596	-0.106309
126.C	3.336015	2.816233	1.352662
127.C	4.801564	2.755711	1.840967
128.C	5.520535	1.491963	1.350651
129.O	5.057032	0.359376	1.510929
130.N	6.720515	1.697177	0.751281
131.N	3.407681	-0.018355	-2.619555
132.C	2.363373	0.891453	-3.088909
133.C	2.442066	2.242374	-2.344579
134.C	2.353126	2.059112	-0.819062

S10. Transition state optimized structure and energy of Boc-protected [1*R*,2*R*]-trans-ACHC hydroxybenzotriazole ester and **7** complex in water (total bond energy: -17 909.8 kcal/mol).

1.C	-4.249018	-2.535600	0.024749
2.C	-4.157601	-5.092158	0.236034
3.C	-4.877959	-3.807003	0.641774
4.C	-2.765075	-2.441612	0.485707
5.C	-2.043960	-3.733203	-0.007253
6.C	-2.669405	-5.006963	0.567752
7.H	-4.256139	-2.635456	-1.067958
8.H	-4.616675	-5.948825	0.743565
9.H	-4.284748	-5.261361	-0.842072
10.H	-5.926190	-3.849532	0.336928
11.H	-4.865988	-3.698344	1.734348
12.H	-2.740684	-2.403593	1.577843
13.C	-1.977905	-1.254322	-0.060662
14.H	-2.100839	-3.757253	-1.101958
15.H	-0.984302	-3.708329	0.245585
16.H	-2.530123	-5.026703	1.657108
17.H	-2.131771	-5.874556	0.166687
18.N	-5.038157	-1.361768	0.391090
19.C	-6.245495	-1.093178	-0.178578
20.O	-6.807098	-1.807436	-1.006522
21.O	-6.730861	0.074847	0.327618
22.C	-8.067779	0.616962	-0.014599
23.H	-4.554611	-0.599817	0.855749
24.C	-8.117975	1.903417	0.820481
25.C	-8.158268	0.950338	-1.508754
26.C	-9.167728	-0.352440	0.435947
27.H	-7.312086	2.585104	0.534067
28.H	-8.021619	1.679759	1.887057
29.H	-9.072516	2.412415	0.661109
30.H	-8.145357	0.052014	-2.126082
31.H	-7.330218	1.598613	-1.809238
32.H	-9.092682	1.486855	-1.700602
33.H	-10.143478	0.129170	0.319189
34.H	-9.043762	-0.610498	1.492345
35.H	-9.165594	-1.269607	-0.153725
36.O	-2.812862	0.227934	1.092533
37.O	-1.901552	-0.869385	-1.191532
38.H	-4.030534	1.063192	-1.659048
39.N	-2.517302	1.488895	0.822971
40.N	-1.760366	3.474008	1.303819
41.C	-2.405455	3.551018	0.095643
42.C	-2.910350	2.274632	-0.235251
43.H	-3.526174	5.245237	-2.606736
44.C	-3.340005	4.422700	-1.919986
45.C	-3.847745	3.135955	-2.236567
46.H	-4.412415	3.005334	-3.156888
47.N	-1.832978	2.232527	1.727402
48.H	-2.228792	5.632902	-0.511733
49.C	-2.618230	4.648400	-0.760058
50.C	-3.644837	2.043874	-1.408410
51.O	1.505330	1.355678	-0.319303
52.N	3.364381	-2.283678	0.324692
53.C	4.056955	-3.023474	-0.733183
54.C	4.484942	-2.089329	-1.890331
55.C	3.278881	-1.337699	-2.473937
56.O	2.235050	-1.925201	-2.788762
57.N	-0.385965	-1.134736	0.680342
58.C	0.102973	-1.697372	1.998273
59.C	1.650701	-1.795387	2.009867
60.C	2.189491	-2.675518	0.867052
61.O	1.571755	-3.687632	0.501395
62.C	2.651693	4.038789	1.921703
63.C	4.872172	2.772834	3.403216
64.C	4.164825	4.014723	3.953015
65.C	2.721453	4.108921	3.451005
66.C	2.498718	1.136814	-4.627054
67.C	1.390902	3.222367	-2.851022
68.C	1.453484	3.429847	-4.367987
69.C	1.414226	2.098821	-5.124420
70.C	5.260051	-3.810155	-0.181617
71.C	5.199138	-2.902975	-2.995077
72.C	6.387736	-3.700267	-2.448598
73.C	5.974925	-4.602455	-1.282144
74.C	-0.408827	-0.883012	3.188717
75.C	2.123192	-2.389342	3.363112
76.C	1.594222	-1.602536	4.563944
77.C	0.071187	-1.475960	4.518195
78.H	2.819645	1.897405	1.702170
79.H	5.358129	3.571080	1.462650
80.H	3.926991	3.364627	-0.598390
81.H	7.241447	0.807928	0.462671
82.H	7.091261	2.524860	0.602100
83.H	1.457910	0.444835	-2.866119
84.H	3.465719	2.718959	-2.573281
85.H	4.346127	0.388194	-2.385875
86.H	3.322747	-3.730387	-1.123933
87.H	5.201323	-1.358565	-1.493696
88.H	3.819025	-1.460893	0.703846
89.H	0.163129	-1.552582	-0.071492
90.H	-0.187023	-0.133368	0.625311
91.H	-0.290656	-2.712984	2.053890
92.H	2.065207	-0.786418	1.913645
93.H	1.609543	4.032141	1.586366
94.H	3.123138	4.932384	1.490207
95.H	5.920058	2.761881	3.724559
96.H	4.405904	1.868447	3.812704
97.H	4.717976	4.914791	3.651165
98.H	4.185442	3.987805	5.049384
99.H	2.130257	3.289220	3.882897
100.H	2.258070	5.040607	3.797170
101.H	2.408388	0.168556	-5.131243
102.H	3.491831	1.534844	-4.875711
103.H	1.491260	4.184973	-2.336728
104.H	0.413591	2.813566	-2.568747
105.H	2.375600	3.969365	-4.624903
106.H	0.619754	4.069716	-4.682655
107.H	0.427480	1.632635	-4.994731
108.H	1.538746	2.268858	-6.200629
109.H	4.908012	-4.486539	0.605414
110.H	5.964668	-3.112441	0.290355
111.H	5.534933	-2.220422	-3.784316
112.H	4.474677	-3.587230	-3.453526
113.H	7.172315	-3.006470	-2.116386
114.H	6.823406	-4.300934	-3.256323
115.H	5.308438	-5.394530	-1.650790
116.H	6.853327	-5.105292	-0.860221
117.H	-1.498974	-0.842914	3.168193
118.H	-0.059850	0.152190	3.098311
119.H	3.217899	-2.409648	3.375931
120.H	1.785833	-3.431283	3.427304
121.H	2.047923	-0.602254	4.578701
122.H	1.907915	-2.101287	5.488520
123.H	-0.384432	-2.464848	4.664507
124.H	-0.286093	-0.844315	5.339351
125.N	3.301844	2.749229	-0.097625
126.C	3.353860	2.787076	1.361945
127.C	4.815342	2.703602	1.859074
128.C	5.514416	1.425930	1.377275
129.O	5.042003	0.298784	1.573916
130.N	6.699528	1.608967	0.752851
131.N	3.445954	-0.001814	-2.624015
132.C	2.414267	0.917173	-3.105067
133.C	2.492750	2.261126	-2.348539
134.C	2.375637	2.066887	-0.825982

S11. Transition state optimized structure and energy of Boc-protected [1*S*,2*S*]-trans-ACHC hydroxybenzotriazole ester and **7** in chloroform (total bond energy: -17 900.7 kcal/mol).

1.C	3.412870	-2.419304	-0.812230	46.H	6.960122	3.104683	-1.492417	91.H	0.145268	0.406142	-3.421275
2.C	2.385070	-4.721352	-1.281199	47.N	1.782820	2.716627	0.548114	92.H	-2.106914	1.463515	-1.649792
3.C	3.223096	-3.874930	-0.324983	48.H	4.077365	6.027620	-0.143889	93.H	-0.952410	2.923445	3.134169
4.C	2.016682	-1.751116	-0.976095	49.C	4.364215	4.995274	-0.329273	94.H	-2.358084	3.477623	4.044055
5.C	1.202002	-2.604671	-1.989110	50.C	5.114657	2.262213	-0.815702	95.H	-5.052183	4.980659	1.202218
6.C	1.034762	-4.059769	-1.543402	51.O	-1.397393	0.051510	1.585636	96.H	-3.642890	4.417281	0.312628
7.C	-2.528297	-1.182016	3.348761	52.N	-3.452525	-0.674582	-1.936553	97.H	-3.651038	5.650109	3.121179
8.H	2.925796	-4.847845	-2.229388	53.C	-4.277090	-1.876817	-2.090342	98.H	-3.037906	6.396613	1.650849
9.H	2.248293	-5.726596	-0.864203	54.C	-4.788154	-2.401591	-0.728794	99.H	-1.238034	4.717773	1.473537
10.H	2.750468	-3.846678	0.666239	55.C	-3.619344	-2.765461	0.202752	100.H	-1.193338	5.424795	3.079966
11.H	4.212846	-4.318163	-0.192859	56.O	-2.687195	-3.477569	-0.174361	101.H	-3.011780	-4.571950	2.729195
12.C	-2.237460	0.058152	2.484109	57.N	0.378660	0.343941	-1.346230	102.H	-3.917842	-3.572627	3.863512
13.C	2.049181	-0.304271	-1.490169	58.C	-0.199720	1.001388	-2.572171	103.H	-1.346369	-0.483730	5.031806
14.H	0.214479	-2.176240	-2.160466	59.C	-1.749219	0.981317	-2.566687	104.H	-0.452599	-1.468588	3.879989
15.H	1.719345	-2.572432	-2.955586	60.C	-2.335554	-0.441936	-2.672251	105.H	-2.543329	-2.455566	5.897398
16.H	0.479281	-4.601894	-2.318007	61.O	-1.837739	-1.277490	-3.434305	106.H	-0.809380	-2.746451	5.960917
17.H	0.415933	-4.094594	-0.637837	62.C	-1.935374	3.394891	3.032826	107.H	-0.901278	-4.131219	3.926412
18.N	4.292567	-1.700195	0.104941	63.C	-4.061518	4.525911	1.320308	108.H	-2.096080	-4.731743	5.067232
19.C	5.636079	-1.917745	0.142870	64.C	-3.160021	5.433846	2.161988	109.H	-5.030936	-1.304409	-4.029441
20.O	6.233517	-2.740766	-0.544235	65.C	-1.794349	4.790903	2.417209	110.H	-6.050702	-0.787164	-2.690018
21.O	6.199673	-1.085045	1.065430	66.C	-2.942374	-3.687095	3.370736	111.H	-6.082620	-3.964373	0.039054
22.C	7.625522	-1.160969	1.447641	67.C	-1.413333	-1.378307	4.401586	112.H	-5.068237	-4.465061	-1.308555
23.H	3.931923	-0.872987	0.562058	68.C	-1.653488	-2.616274	5.272581	113.H	-7.539067	-2.655218	-1.470249
24.C	7.732656	-0.073040	2.525015	69.C	-1.846940	-3.877833	4.425734	114.H	-7.410033	-4.310651	-2.052565
25.C	7.946247	-2.536090	2.048450	70.C	-5.443555	-1.622324	-3.065664	115.H	-5.754140	-3.647252	-3.755687
26.C	8.532789	-0.821370	0.257557	71.C	-5.689398	-3.643049	-0.932788	116.H	-7.169095	-2.614010	-3.911855
27.H	7.066070	-0.291059	3.364468	72.C	-6.841593	-3.383741	-1.907350	117.H	1.434629	2.396048	-2.743309
28.H	7.466316	0.906174	2.117478	73.C	-6.329024	-2.858733	-3.250568	118.H	0.083552	2.992520	-1.787814
29.H	8.757699	-0.021930	2.903005	74.C	0.345159	2.431698	-2.693049	119.H	-3.394073	1.807565	-3.711105
30.H	7.890095	-3.326331	1.299400	75.C	-2.301665	1.775779	-3.783388	120.H	-2.060858	1.218790	-4.696701
31.H	7.252486	-2.770063	2.862050	76.C	-1.737499	3.192213	-3.892692	121.H	-2.086420	3.801152	-3.047310
32.H	8.959503	-2.524591	2.462302	77.C	-0.210332	3.168158	-3.914118	122.H	-2.127851	3.665569	-4.801726
33.H	9.568274	-0.740917	0.603385	78.H	-2.357111	2.293839	1.224402	123.H	0.139121	2.678925	-4.833393
34.H	8.249625	0.140475	-0.180076	79.H	-4.714449	3.235387	2.916889	124.H	0.192228	4.187643	-3.930676
35.H	8.486292	-1.587323	-0.516407	80.H	-3.579834	1.090035	3.618597	125.N	-2.984517	1.155240	2.805430
36.O	2.579080	0.571843	0.157883	81.H	-7.011246	1.509751	0.924175	126.C	-2.835203	2.471335	2.190068
37.O	2.643169	0.121401	-2.439285	82.H	-6.682489	2.372110	2.385574	127.C	-4.217797	3.118611	1.944129
38.H	5.397445	1.233355	-1.003213	83.H	-1.744088	-2.607747	1.938692	128.C	-5.104593	2.252847	1.039162
39.N	2.756590	1.882925	0.100705	84.H	-3.473657	-1.024784	3.887409	129.O	-4.726991	1.838411	-0.060164
40.N	2.202444	3.955400	0.464376	85.H	-4.516439	-1.716810	1.704495	130.N	-6.349483	1.993358	1.513748
41.C	3.476867	3.939213	-0.049064	86.H	-3.615382	-2.635161	-2.514008	131.N	-3.707256	-2.270537	1.467324
42.C	3.857221	2.601461	-0.294678	87.H	-5.394871	-1.613951	-0.261453	132.C	-2.677063	-2.453866	2.487370
43.H	6.319077	5.452985	-1.078007	88.H	-3.819956	0.083792	-1.374750	133.H	1.509895	-1.754392	-0.004584
44.C	5.605959	4.665133	-0.846733	89.H	-0.211065	-0.422624	-1.029920	134.H	3.890242	-2.442764	-1.799789
45.C	5.974203	3.315620	-1.084642	90.H	0.426473	0.995637	-0.561241				

S12. Transition state optimized structure and energy of Boc-protected [1*S*,2*S*]-trans-ACHC hydroxybenzotriazole ester and **7** in water (total bond energy: -17 914.6 kcal/mol).

1.C	4.945469	2.141633	0.340131
2.C	4.709739	4.668988	0.727686
3.C	5.221463	3.566333	-0.198141
4.C	3.414521	1.952751	0.553396
5.C	2.947941	3.051117	1.548750
6.C	3.228651	4.467669	1.037408
7.C	-4.696775	-0.815540	-3.156230
8.H	5.285860	4.658175	1.663185
9.H	4.879005	5.649323	0.266194
10.H	4.754449	3.660506	-1.187819
11.H	6.301084	3.668699	-0.345642
12.C	-4.072295	-1.517370	-1.939413
13.C	3.029061	0.569563	1.098654
14.H	1.881292	2.961468	1.746609
15.H	3.464292	2.897249	2.504254
16.H	2.889894	5.188813	1.791075
17.H	2.632290	4.654042	0.133803
18.N	5.560767	1.148270	-0.532876
19.C	6.896459	0.882649	-0.486906
20.O	7.646367	1.235065	0.420663
21.O	7.252007	0.148094	-1.577005
22.C	8.658365	-0.215345	-1.889372
23.H	5.071487	0.884089	-1.375027
24.C	8.509672	-0.973308	-3.214990
25.C	9.500244	1.050369	-2.092402
26.C	9.240761	-1.136640	-0.810966
27.H	8.066748	-0.332576	-3.983082
28.H	7.876784	-1.856861	-3.091843
29.H	9.492111	-1.301190	-3.565564
30.H	9.634580	1.601871	-1.161433
31.H	9.032796	1.709321	-2.830631
32.H	10.487981	0.768733	-2.469959
33.H	10.219665	-1.498328	-1.140595
34.H	8.597537	-2.007566	-0.656117
35.H	9.368298	-0.618846	0.139571
36.O	2.968481	-0.405982	-0.536920
37.O	3.580967	-0.049202	1.965579
38.H	5.610647	-1.991631	-0.181468
39.N	2.706676	-1.693398	-0.378882
40.N	1.412690	-3.442556	-0.292856
41.C	2.713862	-3.867853	-0.176907
42.C	3.570307	-2.746256	-0.224302
43.H	5.071525	-6.265836	0.159132
44.C	4.622063	-5.281849	0.049347
45.C	5.469253	-4.144177	-0.003439
46.H	6.544636	-4.288811	0.067712
47.N	1.425385	-2.136192	-0.414917
48.H	2.593404	-6.033853	0.001181
49.C	3.244944	-5.164332	-0.036598
50.C	4.965720	-2.860425	-0.141714
51.O	-2.851033	-1.549974	-1.756298
52.N	-2.659611	2.083146	1.291502
53.C	-3.238929	3.334159	0.788465
54.C	-4.278147	3.081996	-0.324272
55.C	-3.682021	2.349351	-1.537259
56.O	-2.575323	2.648294	-2.004933
57.N	1.214141	0.512348	1.202149
58.C	0.527308	0.430310	2.535856
59.C	-1.003603	0.663848	2.418248
60.C	-1.379075	1.984519	1.718292
61.O	-0.552693	2.900809	1.582575
62.C	-4.391431	-4.336340	-0.165622
63.C	-5.272705	-3.409745	2.495638
64.C	-5.052901	-4.898773	2.212460
65.C	-4.022234	-5.115396	1.101118
66.C	-4.766841	1.315440	-4.544013
67.C	-4.402520	-1.614524	-4.446247
68.C	-4.970586	-0.930823	-5.693918
69.C	-4.478853	0.513934	-5.817830
70.C	-3.851377	4.156597	1.940953
71.C	-4.887413	4.425368	-0.801961
72.C	-5.482544	5.244229	0.346455
73.C	-4.455636	5.477691	1.456281
74.C	0.797931	-0.917132	3.223237
75.C	-1.621426	0.635034	3.843361
76.C	-1.324980	-0.672001	4.582287
77.C	0.174021	-0.967864	4.621615
78.H	-3.646066	-2.404742	0.450298
79.H	-6.608371	-3.017452	0.852637
80.H	-5.936328	-2.090580	-1.359332
81.H	-7.316020	0.264852	1.793131
82.H	-7.885147	-1.280687	1.266506
83.H	-3.094832	0.609860	-3.372107
84.H	-5.786502	-0.778207	-3.025239
85.H	-5.391398	1.263905	-1.659147
86.H	-2.408034	3.895406	0.356182
87.H	-5.090979	2.473848	0.093484
88.H	-3.290608	1.312729	1.478294
89.H	0.882752	1.347376	0.722296
90.H	0.964676	-0.291726	0.622186
91.H	0.950335	1.235565	3.141548
92.H	-1.439471	-0.164027	1.842861
93.H	-3.610267	-4.450007	-0.924754
94.H	-5.316417	-4.744613	-0.594785
95.H	-6.061932	-3.281300	3.245436
96.H	-4.360056	-2.973021	2.918384
97.H	-6.006707	-5.360752	1.922677
98.H	-4.729940	-5.398823	3.133752
99.H	-3.031623	-4.794295	1.452700
100.H	-3.936203	-6.182643	0.864265
101.H	-4.348780	2.324651	-4.626520
102.H	-5.851777	1.428929	-4.415276
103.H	-4.820649	-2.623064	-4.346825
104.H	-3.316713	-1.729264	-4.550172
105.H	-6.068485	-0.941551	-5.649636
106.H	-4.687496	-1.506122	-6.583906
107.H	-3.397616	0.516953	-6.013993
108.H	-4.953042	1.005769	-6.675700
109.H	-3.073532	4.347513	2.687977
110.H	-4.626524	3.556293	2.436063
111.H	-5.656361	4.221170	-1.555359
112.H	-4.102641	5.008464	-1.299726
113.H	-6.355997	4.719816	0.758059
114.H	-5.847493	6.202054	-0.043956
115.H	-3.654784	6.131039	1.083053
116.H	-4.917770	6.001641	2.301538
117.H	1.872137	-1.086437	3.291610
118.H	0.384135	-1.725893	2.607210
119.H	-2.701721	0.784217	3.774042
120.H	-1.220897	1.482704	4.414421
121.H	-1.850060	-1.498210	4.084503
122.H	-1.729804	-0.611492	5.599608
123.H	0.677486	-0.237578	5.269595
124.H	0.358290	-1.954638	5.061629
125.N	-4.961910	-2.100233	-1.094109
126.C	-4.593675	-2.834884	0.115176
127.C	-5.652644	-2.622130	1.219996
128.C	-5.838095	-1.138124	1.567087
129.O	-4.893672	-0.411570	1.897490
130.N	-7.112525	-0.685321	1.518581
131.N	-4.481598	1.397260	-2.076415
132.C	-4.184161	0.636254	-3.290059
133.H	2.899641	2.096074	-0.403005
134.H	5.430751	2.035803	1.314317

References

[1] a) P. M. Boerrigter, G. T. Velde, E. J. Baerends, *International Journal of Quantum Chemistry* **1988**, *33*, 87-113; b) O. V. C. Fonseca Guerra, J. G. Snijders, G. te Velde, E. J. Baerends, in *Methods and Techniques for Computational Chemistry* (Ed.: G. C. E. Clementi), STEF, Cagliari, **1995**, pp. 305-395; c) T. Z. E.J. Baerends, J. Autschbach, D. Bashford, A. Bérces, F.M. Bickelhaupt, C. Bo, P.M. Boerrigter, L. Cavallo, D.P. Chong, L. Deng, R.M. Dickson, D.E. Ellis, M. van Faassen, L. Fan, T.H. Fischer, C. Fonseca Guerra, M. Franchini, A. Ghysels, A. Giammona, S.J.A. van Gisbergen, A.W. Götz, J.A. Groeneveld, O.V. Gritsenko, M. Grüning, S. Gusarov, F.E. Harris, P. van den Hoek, C.R. Jacob, H. Jacobsen, L. Jensen, J.W. Kaminski, G. van Kessel, F. Kootstra, A. Kovalenko, M.V. Krykunov, E. van Lenthe, D.A. McCormack, A. Michalak, M. Mitoraj, S.M. Morton, J. Neugebauer, V.P. Nicu, L. Noodleman, V.P. Osinga, S. Patchkovskii, M. Pavanello, P.H.T. Philipsen, D. Post, C.C. Pye, W. Ravenek, J.I. Rodríguez, P. Ros, P.R.T. Schipper, G. Schreckenbach, J.S. Seldenthuis, M. Seth, J.G. Snijders, M. Solà, M. Swart, D. Swerhone, G. te Velde, P. Vernooijs, L. Versluis, L. Visscher, O. Visser, F. Wang, T.A. Wesolowski, E.M. van Wezenbeek, G. Wiesenekker, S.K. Wolff, T.K. Woo, A.L. Yakovlev, SCM, Theoretical Chemistry, Vrije Universiteit, Amsterdam, Amsterdam, **2016**; d) C. Fonseca Guerra, J. G. Snijders, G. te Velde, E. J. Baerends, *Theoretical Chemistry Accounts* **1998**, *99*, 391-403; e) P. V. J. G. Snijders, E. J. Baerends, *At. Nucl. Data Tables* **1981**, *26*, 483-509; f) G. te Velde, F. M. Bickelhaupt, E. J. Baerends, C. Fonseca Guerra, S. J. A. Van Gisbergen, J. G. Snijders, T. Ziegler, *Journal of Computational Chemistry* **2001**, *22*, 931-967; g) G. T. Velde, E. J. Baerends, *Journal of Computational Physics* **1992**, *99*, 84-98; h) L. Versluis, T. Ziegler, *Journal of Chemical Physics* **1988**, *88*, 322-328.

[2] E. J. Baerends, D. E. Ellis, P. Ros, *Chemical Physics* **1973**, *2*, 41-51.

[3] N. C. Handy, A. J. Cohen, *Molecular Physics* **2001**, *99*, 403-412.

[4] C. Lee, W. Yang, R. G. Parr, *Physical Review B* **1988**, *37*, 785 -789.

[5] a) J. Baker, P. Pulay, *Journal of Chemical Physics* **2002**, *117*, 1441-1449; b) A. P. Bento, M. Sola, F. M. Bickelhaupt, *Journal of Chemical Theory and Computation* **2008**, *4*, 929-940; c) V. A. Guner, K. S. Khuong, K. N. Houk, A. Chuma, P. Pulay, *The Journal of Physical Chemistry A* **2004**, *108*, 2959-2965.

[6] a) A. Klamt, *Journal of Physical Chemistry* **1995**, *99*, 2224-2235; b) A. Klamt, V. Jonas, *Journal of Chemical Physics* **1996**, *105*, 9972-9981; c) A. Klamt, G. Schuurmann, *Journal of the Chemical Society-Perkin Transactions 2* **1993**, 799-805.

[7] C. C. Pye, T. Ziegler, *Theoretical Chemistry Accounts* **1999**, *101*, 396-408.
